# Ultrafast dense DNA functionalization of quantum dots and rods for scalable 2D array fabrication with nanoscale precision

**DOI:** 10.1126/sciadv.adh8508

**Published:** 2023-08-11

**Authors:** Chi Chen, Xin Luo, Alexander E. K. Kaplan, Moungi G. Bawendi, Robert J. Macfarlane, Mark Bathe

**Affiliations:** ^1^ Department of Biological Engineering, Massachusetts Institute of Technology, Cambridge, MA 02139, USA.; ^2^ Department of Materials Science and Engineering, Massachusetts Institute of Technology, Cambridge, MA 02139, USA.; ^3^ Department of Chemistry, Massachusetts Institute of Technology, Cambridge, MA 02139, USA.

## Abstract

Scalable fabrication of two-dimensional (2D) arrays of quantum dots (QDs) and quantum rods (QRs) with nanoscale precision is required for numerous device applications. However, self-assembly–based fabrication of such arrays using DNA origami typically suffers from low yield due to inefficient QD and QR DNA functionalization. In addition, it is challenging to organize solution-assembled DNA origami arrays on 2D device substrates while maintaining their structural fidelity. Here, we reduced manufacturing time from a few days to a few minutes by preparing high-density DNA-conjugated QDs/QRs from organic solution using a dehydration and rehydration process. We used a surface-assisted large-scale assembly (SALSA) method to construct 2D origami lattices directly on solid substrates to template QD and QR 2D arrays with orientational control, with overall loading yields exceeding 90%. Our fabrication approach enables the scalable, high fidelity manufacturing of 2D addressable QDs and QRs with nanoscale orientational and spacing control for functional 2D photonic devices.

## Teaser

Dehydration and surface-assisted assembly enable rapid, scalable quantum dot and quantum rod 2D arrays with nanoscale precision.

## INTRODUCTION

Quantum dots (QDs) and quantum rods (QRs) exhibit appealing features of bright and tunable narrowband photoluminescence (PL) emission that have attracted extensive interest in the wake of numerous successful state-of-the-art device applications (*1*). For example, QD/QR-based devices are key components of next-generation displays (*2*–*4*), particularly in the field of micro-light–emitting diodes (μ-LEDs), which offer advantages compared with organic LED and liquid-crystal displays in terms of brightness, color, minimum pixel size, and lifetime (*5*). QRs are particularly interesting because of their polarized light emission (*6*), which has the potential to improve the optical efficiency of display equipment (*4*, *7*). For example, it has been reported that the alignment of emitter dipoles along the long axis of rod can achieve as high as 40% out-coupling efficiency when incorporated into an LED structure (*8*, *9*), which is considerably greater than the typical out-coupling efficiency (<25%) of QD-based LEDs (*10*). However, high-quality polarized light sources with QRs require the alignment of QRs along their long axes at the nano-to-micro scale, which is still technically challenging to produce reliably (*4*, *7*, *11*). Previously reported alignment methods have controlled QR assembly and alignment using macroscale external forces (*12*–*16*) or polymer matrices (*11*, *17*–*19*), which offered some extent of global control over QR orientation but lacked the capability to address individual QRs to control interparticle distances and orientations. Evidence suggests that QDs/QRs lacking spatial control typically suffer from “self-quenching” when they are deposited as thin films (*20*, *21*), resulting from Förster resonant energy transfer (FRET) of excitons within their inhomogeneous size distribution (*2*). These considerations will be of paramount importance for advanced display applications such as virtual reality and augmented reality devices composed of μ-LEDs that have pixel sizes that are only a few microns or less (*22*). QDs are also key candidates for quantum computing, quantum sensing, and quantum metrology through integrated quantum photonics (*1*, *23*). For all of these applications, an important challenge to use QDs in these devices is to accurately place and align controlled numbers and arrangements of QDs within nanometer- to micron-scale photonic circuits (*24*, *25*). Thus, approaches to scalably produce QD and QR two-dimensional (2D) arrays with nanoscale precision are highly desirable.

DNA nanotechnology, and in particular the DNA origami method, offers unparalleled capability to program the positions and orientations of nanomaterials at the nano- to micro-scale with subnanometer precision and intrinsic scalability using solution-based, bottom-up self-assembly (*26*, *27*). DNA origami–based nanomaterial integration into photonic devices represents one of the most promising routes toward this goal (*28*–*30*). While DNA-based approaches are in many ways ideal to address the technical challenges of incorporating QDs/QRs into optical devices, two primary challenges must be addressed before such methods can be used. First, typical DNA grafting methods result in low conjugation yields of DNA ligands to the QD/QR surfaces and thus limited stability in aqueous buffer conditions required for DNA hybridization. Since high-quality colloidal semiconductor nanocrystals are synthesized in organic solvent with hydrophobic ligands (organic QDs and QRs), common strategies to conjugate DNA to QDs and QRs first seek to transfer organic QDs and QRs to aqueous medium, where water-soluble DNA can then be conjugated to aqueous QDs and QRs via interactions with their existing ligands or with the inorganic shell (*31*). Thiolated single-stranded DNA (ssDNA) is the most popular DNA derivative to attach to the QD and QR surface (*31*). However, existing approaches are time consuming, typically requiring several days due to the need for a separate phase-transfer step and a prolonged DNA conjugation process (*32*–*35*). Recently, Ye *et al.* (*36*) reported a one-step ligand-exchange method to produce DNA-conjugated QDs from organic solvent, which simplifies the functionalization process but still requires several hours to complete. Moreover, the products of these preceding thiolated DNA conjugation approaches suffer from limited numbers of functional ssDNAs per QD and QR (table S1) (*32*–*36*), which decreases their hybridization efficiency to complementary ssDNA and colloidal stability in high salt concentration buffers (*37*). This will in turn reduce the loading yield of QDs/QRs onto DNA origami structures. DNA with a phosphorothioate-modified backbone (ps-DNA) is another class of functional groups that exhibit affinity toward shells of QDs. For example, Yan *et al.* (*38*) developed a method to increase the colloidal stability of ps-DNA functionalized QDs by cogrowth of additional CdS or ZnS and DNA shells on the particles’ surface. Subsequently, this method was adopted by others to control QD arrangements on DNA origami (*39*). Similarly, Kelley and co-workers (*40*) reported a one-step synthesis method for CdTe QDs with ps-DNA strands embedded, incorporating one to five ssDNA strands available for hybridization. However, cogrowth procedures of phase transferred QDs with DNA and directly synthesizing QDs in aqueous solution often results in inhomogeneous QD sizes and the formation of electron or hole traps inside the interior of the lattice due to poor QD crystallinity, leading to broad PL emissions and reduced quantum yields (*41*). Therefore, a fast and facile method for preparation of QDs and QRs with high DNA ligand density directly from organic solvent would substantially lower the barrier for the integration of QDs/QRs with DNA nanostructures.

Another key challenge to manufacturing functional structures with DNA origami that can readily be incorporated into devices is to transfer solution-synthesized origami-nanoparticle complexes to device substrates with controlled positioning and alignment while preserving their structural fidelity and function in the dry state. We recently developed a class of rigid 2D wireframe six-helix bundle (6HB) DNA origami (*42*, *43*) that allows for the programming of arbitrary 2D geometries with high structural fidelity, planarity, and rigidity, which can serve as robust templates to organize QDs/QRs on solid substrates. However, direct transfer of larger-scale soft materials such as DNA superstructures of these wireframe DNA origamis (*44*) from solution to a surface often suffers from aggregation and overlapping structures during the deposition and drying process. One promising method to circumvent this problem is to use surface-assisted assembly, whereby building blocks bound to the surface of the substrate can diffuse freely in 2D and self-organize into well-defined patterns. Self-assembly of DNA tiles (*45*) and origami structures (*46*–*49*) on lipid bilayer surfaces have been reported to form various 2D lattices. However, without additional treatment, these assemblies may collapse upon drying due to the soft nature of the lipid substrate. More recently, long-range patterns of origamis on solid substrates have been demonstrated with the help of monovalent cations (Na^+^) to promote surface diffusion and self-organization (*50*–*55*). However, these examples only relied on either nonspecific blunt-end interactions (*50*, *51*, *55*) or merely surface crowding and shape matching to fit symmetrical origamis into 2D patterns (*52*–*54*). Lattice defects like grain boundary slipping are therefore frequently observed. Anisotropic spatial arrangements of DNA oligos of distinct sequences in otherwise symmetrical origami shapes are not aligned across origami units in the lattice because the inter-origami packing interactions are nonspecific. Hence, these 2D patterns are sub-optimal for controllably templating secondary, functional materials that require nanometer-scale spatial control.

**Fig. 1.**
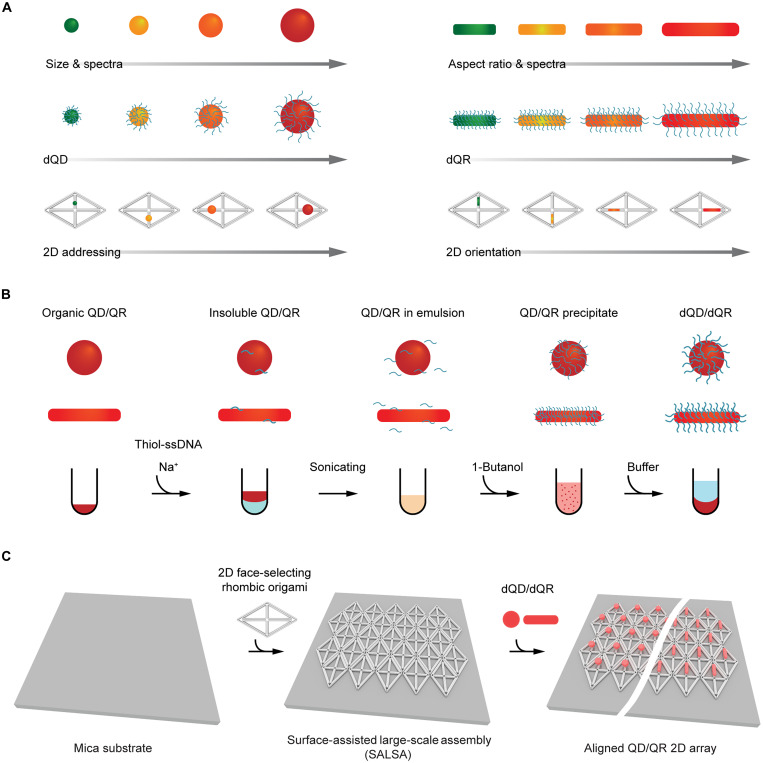
Strategy to fabricate scalable QD/QR 2D array with nanoscale precision using dehydration-assisted DNA conjugation and SALSA. (**A**) Schematic of capabilities of dehydration-assisted DNA conjugation to QDs/QRs (dQDs/dQRs). (**B**) Schematic of the workflow to prepare dQDs/dQRs. (**C**) Schematic of fabrication of scalable QD/QR 2D arrays with nanoscale spatial and orientational precision using SALSA and dQD/dQR.

Here, we developed an ultrafast sonication-mediated and dehydration-assisted functionalization method to conjugate a dense layer of DNA strands to QDs and QRs (dQDs/dQRs) from their original organic solvent to aqueous buffer, which substantially shortens the time required for their synthesis from a few days to a few minutes. This approach can be applied to QDs and QRs with various sizes, aspect ratios, spectra, and shell surfaces (Fig. 1A). The dQDs/dQRs have high DNA density that endows them with excellent stability in a variety of salted aqueous buffers, as well as outstanding binding affinity and fidelity to DNA origami structures. Specifically, our approach first incubates QDs or QRs dispersed in organic solvent with thiol-derivatized ssDNA and Na^+^, followed by sonication until emulsion formation. Then, 1-butanol is added to instantly dehydrate the mixture, which condenses ssDNA onto the surface of QDs and QRs for efficient conjugation. Last, aqueous buffer is added to rehydrate and recover the dQDs/dQRs produced by dehydration-assisted conjugation with high-density surface DNA (Fig. 1B).

We then seek to fabricate arrays of these densely DNA-functionalized QDs/QRs with wireframe origami templates. Building on prior work by Woo and Rothemund (*50*) and Aghebat Rafat *et al.* (*51*), we developed the surface-assisted large-scale assembly (SALSA) method to construct 2D origami lattices directly on a solid substrate to template QD and QR 2D arrays with a full control over internanoparticle spacing and orientation (Fig. 1C). Specifically, 2D origami with QD/QR binding overhangs were first assembled into 2D lattices directly on the mica surface by using matching lateral overhangs/vacancies on each origami edge. Monovalent cations, thermal annealing, and face-selecting overhangs were used to allow for surface diffusion, error correction, and proper landing side selection to achieve large, continuous lattice grains. QDs/QRs functionalized with high-density complementary DNA strands can then assemble onto the origami lattice with controlled orientations and spacings determined by the underlying origami arrangement.

## RESULTS

### Dehydration-assisted DNA conjugation to QDs/QRs

In a dehydration-assisted DNA conjugation process (see Methods), commercial QDs/QRs dispersed in an organic solvent are initially mixed with an aqueous solution containing an excess thiolated ssDNA and sodium salt, forming a phase separated liquid double layer (Fig. 1B). The mixture is then sonicated for several minutes until an emulsion is formed [fig. S1A(a)], and the organic QDs/QRs are transferred to the aqueous phase through inefficient ligand exchange with a few thiolated ssDNA. Upon the addition of a large volume of 1-butanol and brief vortexing, the liquid from the emulsion solution (including both water and organic solvent) is absorbed into the 1-butanol phase, leaving insoluble excess thiolated DNA condensed onto the dehydrated QDs/QRs [fig. S1A(b)]. This promotes efficient contact and DNA conjugation to the surface of QDs/QRs. This state is similar to what was described as a “solid solution” of gold nanoparticles and DNA in prior work by Deng *et al.* (*56*). An aqueous buffer is then added directly to the mixture to dissolve and recover the solid solution as densely DNA-functionalized QDs/QRs. Alternatively, the solid solution can be pelleted using a benchtop spinner [fig. S1A(c)] and the 1-butanol can be removed [fig. S1A(d)], followed by redispersal of the pellet in an aqueous buffer [fig. S1A(e)]. The process is not affected by initial organic solvents of QDs/QRs, i.e., chloroform, toluene, or hexane, which are common solvents for commercial QDs/QRs. Although sonicating organic QDs/QRs with aqueous thiolated DNA and sodium salt can transfer the QDs/QRs to the aqueous phase (fig. S1B), without subsequent 1-butanol dehydration, they are unstable in aqueous buffer and tend to form large QD/QR clusters (fig. S1, C to E), which is likely due to insufficient ligand exchange.

**Fig. 2.**
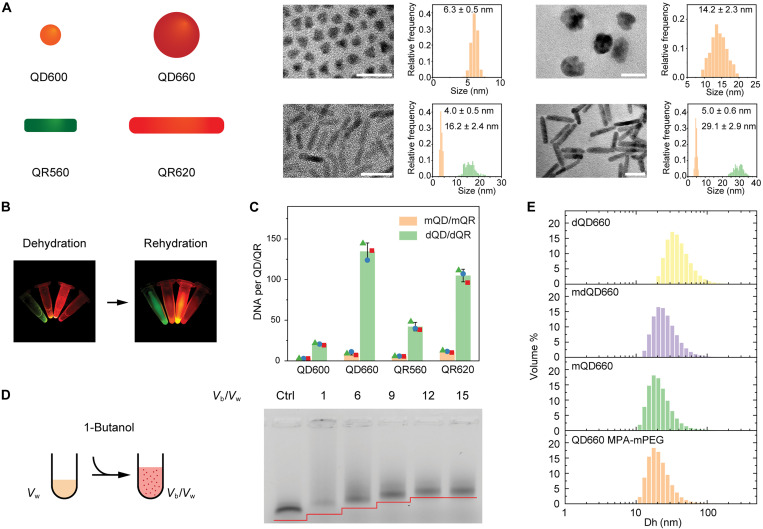
Dehydration-assisted DNA conjugation to QDs and QRs. (**A**) TEM images and size distributions of QD600, QD660, QR560, and QR620. (**B**) Digital photo of QD and QR (from left to right: QR560, QD600, QR620, and QD660) in the dehydration and rehydration process. (**C**) DNA loading density of various QDs and QRs with the conventional method (mQD/mQR, orange) versus dehydration-assisted conjugation (dQD/dQR, green) (green triangle, red square, and blue circle represent each of three replicates per group). (**D**) AGE image of dQD660 with various 1-butanol/water ratio. (**E**) Hydrodynamic diameter (Dh) of DNA-conjugated QDs using various methods (from bottom to top: QD660 MPA-mPEG, mQD660, mdQD660, and dQD660).

To generalize our dehydration-assisted approach of preparing QDs/QRs with high-density DNA ligands, we systematically investigated the impact of QD/QR surface area, DNA:QD/QR ratio, Na^+^ and phase transfer catalyst, dehydration volume ratio, and dehydration time on the DNA density per dQD/dQR. Commercial organic 6- and 14-nm CdSe/ZnS QDs with emission wavelengths of 600 (QD600) and 660 nm (QD660), respectively, and 4/16 nm (diameter/length) and 5/29 nm CdSe/CdS QRs with emission wavelengths of 560 (QR560) and 620 nm (QR620), respectively, were selected to prepare dQD600, dQD660, dQR560, and dQR620 using dehydration-assisted DNA conjugation (Fig. 2, A and B, and fig. S2). In contrast, mQD600, mQD660, mQR560, and mQR620 were prepared according to previous literature that first transferred organic QDs/QRs to aqueous medium by ligand exchange using 3-mercaptopropionic acid (MPA) and *O*-(2-mercaptoethyl)-*O*′-methyl-hexa(ethylene glycol) (mPEG) and then conjugated with thiolated DNA (mQDs/mQRs) (*32*–*35*). To quantify DNA density, 5′-thiolated DNA (21 nt) with an extra fluorescent modifier (FAM) at the 3′ terminus was used to prepare dQDs/dQRs and mQDs/mQRs. Using an agarose gel electrophoresis (AGE) assay, we observed that the electrophoretic mobility of dQD660 relative to mQD660 decreased due to the high-density DNA on surface, which was further demonstrated by the stronger fluorescence signal from the FAM channel (fig. S3). DNA concentration was determined by FAM fluorescence relative to a calibration curve (fig. S4). First, we investigated the effect of DNA:QD/QR ratio on the DNA density of thiolated ssDNA per QD/QR. For dQR620, we found that the DNA density reached saturation at a DNA:QR ratio of 500:1. For dQD600 and dQR560, we did not observe a difference in DNA density between a DNA:QD/QR ratio of 200:1 and 1000:1. For QD660, we did not observe a difference in DNA density between a DNA:QD ratio of 500:1 and 1000:1 (fig. S5). Therefore, we chose to use a DNA:QD/QR ratio of 200 for QD600 and QR560 and a ratio of 500 for QD660 and QR620, considering both conjugation efficiency and minimization of cost. The DNA numbers per dQD600, dQD660, dQR560, and dQR620 were 21, 135, 42, and 105, respectively (Fig. 2C). By comparison, the DNA numbers per mQD600, mQD660, mQR560, and mQR620 were 3, 9, 6, and 12, respectively (Fig. 2C). Considering the surface area of 125, 633, 229, and 496 nm^2^ for QD600, QD600, QR560, and QR620, the DNA densities were 0.17 to 0.21 per nm^2^ for dQDs/dQRs and 0.015 to 0.025 per nm^2^ for mQDs/mQRs (table S2). These results showed that our approach substantially increases DNA surface density up to 10-fold compared with the traditional method (*32*–*34*). Moreover, we carefully examined the effect of different ligands on the luminescent properties of QDs/QRs before and after conjugation. We found that ligand exchange generally reduced their quantum yields, likely due to the creation of additional PL quenching channels arising from surface defects and changes in ligands after phase transfer (fig. S6). However, our method produced high-density DNA-conjugated QDs/QRs directly from organic phase, avoiding the introduction of MPA and mPEG during the phase transfer process required by traditional methods. Hence, dQDs/dQRs generally presented higher quantum yields. The quantum yields of dQD600, dQR560, and dQR620 were measured to be 0.20 ± 0.02, 0.22 ± 0.04, and 0.56 ± 0.04, respectively, which are significantly higher than those of mQD600, mQR560, and mQR620 obtained using the traditional method (0.13 ± 0.02, 0.09 ± 0.03, and 0.28 ± 0.03, respectively) (fig. S6 and table S3). There was no significant difference between dQD660 (0.19 ± 0.03) and mQD660 (0.16 ± 0.02) (*P* values ≥ 0.05), which may be due to the greater ZnS shell thickness of QD660 that may decrease the effect of surface defects and ligands. In addition, we found two key factors to prepare high-density DNA functionalization of QDs and QRs, namely, Na^+^ and 1-butanol/water volume ratio. An AGE assay was used to characterize DNA loadings on QD660 based on increased gel retardation. We first prepared dQD660 using thiolated DNA (51 nt) with or without NaOH and the phase transfer catalysts trioctylphosphine oxide (TOPO) and tetrabutylammonium bromide (TBAB). We first tested NaOH instead of NaCl as a control, as NaOH has been shown to be necessary in the previous QD/QR phase transfer protocols (*30*, *57*, *58*). In these work, NaOH was reported to induce deprotonation of thiol groups and facilitate the phase transfer of QDs/QRs (*59*). The AGE gel image showed that the DNA density increased in the presence of NaOH, while the TBAB and TOPO had a negligible effect (fig. S7). We found that NaCl is also capable of facilitating our dehydration-assisted DNA conjugation process. Through AGE gel imaging and DNA density calculations, we found no significant difference in the resulting DNA loading density between using NaOH and NaCl (fig. S8 and table S4). This suggested that the protonation state of thiols is not critical for the success of our method. Moreover, we prepared dQD660 using different 1-butanol/water volume ratio and found that a 1-butanol/water volume ratio of 12:1 led to the highest DNA loading per QD660 (Fig. 2D). Meanwhile, various dehydration times were tested for dQD660 preparation. AGE imaging showed that dQD660 was formed immediately during dehydration. Prolonged aging in the dehydrated state was not needed (fig. S9), consistent with previous observations using the dehydration method to prepare gold nanoparticle based spherical nucleic acids (*56*). To further test dehydration-assisted approach, we prepared DNA (51 nt)–conjugated QD660 using the dehydration method from aqueous QD660 MPA-mPEG (mdQD660) (fig. S10). We used dynamic light scattering (DLS) to characterize DNA density since a high-density DNA on the surface will increase the hydrodynamic diameter (Dh) of QDs (*60*). DLS results showed that the average Dh of mQD660 (~20.9 nm) was similar to QD660 MPA-mPEG (~20.6 nm), while the mdQD660 and dQD660 shifted to ~25.3 and ~38.6 nm, respectively (Fig. 2E). The increased Dh of ~18 nm for dQD660 was consistent with the length of 51-nt DNA strands oriented perpendicularly to the particle surface, which will endow the particle with enhanced binding affinity to complementary DNA strands (*60*). The reason for the limited DNA density increases of mdQD660 can be explained by a competition between thiolated ssDNA and small-molecule thiol ligands on the aqueous QD660 (MPA and mPEG). Once the QD surface is occupied by thiol ligands during phase transfer, it will be difficult for thiolated ssDNA to replace them. Conversely, the initial hydrophobic ligands (octadecylamine or oleic acid) on organic QDs in the method presented here have lower affinity to the QD surface and can easily be replaced by thiolated DNA during the dehydration process. Thus, our approach enables the preparation of a library of dQDs/dQRs with 10-fold higher DNA density within a few minutes directly from QDs or QRs dispersed in organic solvent.

### Hybridization and loading efficiency of QD/QR-DNA origami assemblies

Mono-mercapto ligand capped QDs/QRs usually suffer from poor colloidal stability due to dynamic thiol-ZnS or thiol-CdS interactions (*35*). To evaluate the colloidal stability of dQDs, diluted (5 nM) mQD660, mQD660 with extra thiolated-DNA in solution, and dQD660 were incubated in 500 mM NaCl at room temperature. The mQD660 aggregated on the tube wall after 3 days of incubation, while the latter two only had minor adsorption on the tube (fig. S11). Thus, high-density DNA ligands on these QDs/QRs endowed them with exceptional colloidal stability in high-salt aqueous buffer even under diluted conditions.

**Fig. 3.**
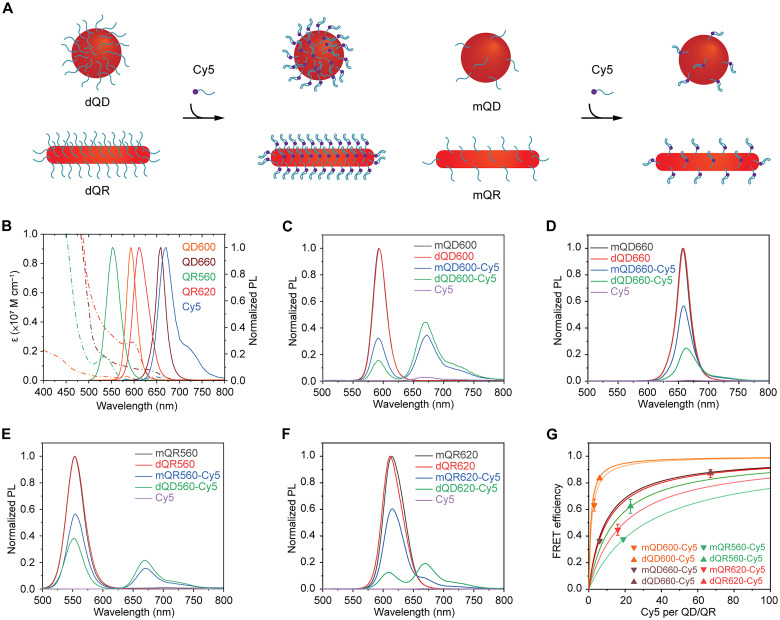
Hybridization availability of DNA functionalized QDs and QRs. (**A**) Schematic of hybridization availability of dQDs/dQRs and mQDs/mQRs. (**B**) Extinction coefficient (dash-dotted line) and PL spectra (solid line) of QD600, QD660, QR560, QR620, and Cy5. Representative PL spectra of (**C**) QD600-Cy5, (**D**) QD660-Cy5, (**E**) QR560-Cy5, and (**F**) QR620-Cy5 FRET pairs. (**G**) FRET efficiencies as a function of acceptors calculated theoretically [solid curves: *R* = 5.8 nm *R*_0_ = 5.7 nm (orange) or 5.3 nm (light orange); *R* = 9.7 nm *R*_0_ = 6.7 nm (wine); *R* = 7.3 nm *R*_0_ = 4.7 nm (green) or 4.1 nm (light green); and *R* = 11 nm *R*_0_ = 7.5 nm (red) or 6.7 nm (light red)] and from QD/QR emission intensities.

To evaluate the hybridization ability of dQDs/dQRs, dQD600, dQD660, dQR560, and dQR620 were incubated with a complementary ssDNA with a FAM (Cy5) at the 3′ terminus (Fig. 3A). For comparison, mQD600, mQD660, mQR560, and mQR620 were also incubated with a complementary ssDNA labeled with Cy5 at the same condition (Fig. 3A). The conjugated dye on the complementary ssDNA provided a distinct measurable signal in the absorbance and emission spectra of the QD/QR-dye hybrids (Fig. 3B), which we used to quantify FRET efficiency with steady-state measurements according to Eqs. 1 to 3 (Methods) and table S5. The fluorescence emission spectra of the QD/QR alone and in the presence of Cy5 showed that dQD/dQR-Cy5 FRET pairs had substantially higher QD/QR donor quenching efficiencies and Cy5 acceptor sensitized intensities, likely due to more Cy5 acceptors around the dQDs/dQRs that provided additional de-excitation pathways (Fig. 3, C to F) (*61*, *62*). Compared with the mQD/mQR-Cy5 FRET, FRET efficiency of dQD/dQR-Cy5 FRET pairs calculated from steady-state measurement increased from 63 ± 4% to 83 ± 1%, from 36 ± 1% to 88 ± 1%, from 38 ± 1% to 62 ± 5%, and from 45 ± 4% to 87 ± 3% (mean ± SD; *n* = 3) for QD600-Cy5, QD660-Cy5, QR560-Cy5, and QR620-Cy5, respectively, indicating an increasing number of dye acceptors due to the high-density of DNA functionalization on the QD/QR surface (Fig. 3G). We estimated the distance between the QD and Cy5 to be the sum of the QD radius and the ssDNA spacer length calculated from the bare worm-like chain model assuming a persistence length of 0.75 nm and a contour length of 0.56 nm per base for ssDNA (*63*, *64*). By comparing theoretical estimates from Eq. 4 (Methods) with experimentally measured QD-Cy5 FRET efficiencies, we further estimated that the number of Cy5 dyes increased from 3 to 6 between mQD600 and dQD600 and from 6 to 67 between mQD660 and dQD660 (Fig. 3G). It should be noted that the distance of Cy5 to mQDs/mQRs might be different from that of dQDs/dQRs due to the different conformations that the conjugated DNA might adopt (fig. S12A). Effects of this uncertainty on the FRET calculations are shown in fig. S12 (B and C). For QRs, this equidistant theoretical model is not applicable to QR-Cy5 FRET pairs due to varied donor-acceptor distance between the QR fluorescence center and Cy5 on the surface DNA. However, using an average donor-acceptor distance, we observed an increasing trend of inferred Cy5 number (Fig. 3G).

**Fig. 4.**
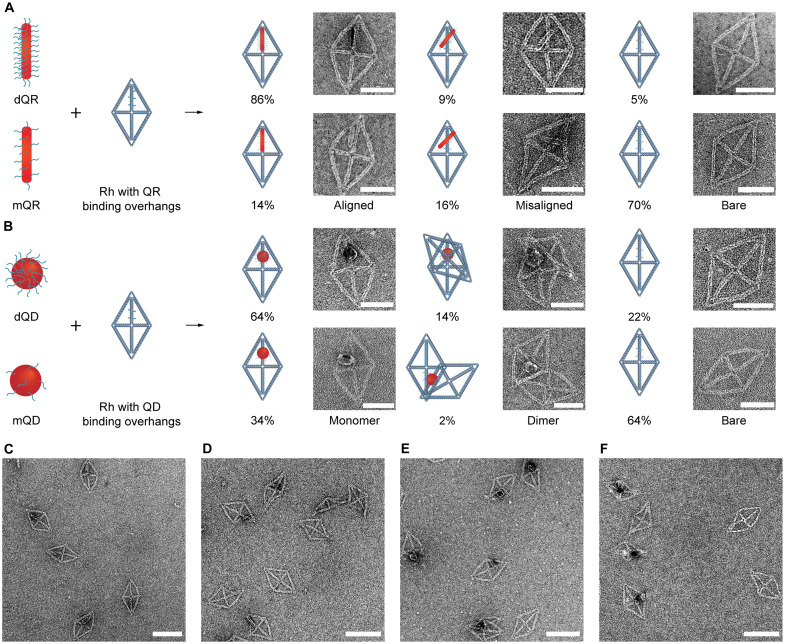
Loading efficiency of QDs/QRs to 2D Rh. Schematic and TEM images of (**A**) dQR620/mQR620-origami and (**B**) dQD660/mQD660-origami assemblies. The percentage of aligned (within 30°) and misaligned Rh-QR assemblies and monomer and dimer of Rh-QD assemblies were calculated from TEM images. Representative TEM images of (**C**) Rh-dQR, (**D**) Rh-mQR, (**E**) Rh-dQD, and (**F**) Rh-mQD. Scale bars, 50 nm [(A) and (B)] and 100 nm [(C) to (F)].

Having demonstrated the highly efficient hybridization ability of dQDs and dQRs, we next sought to test the loading efficiency of dQDs/dQRs onto DNA origami structures. A rigid 6HB wireframe rhombic DNA origami (Rh) was designed using ATHENA (*65*), an open-source graphical user interface software for automated sequence design of 2D and 3D wireframe scaffolded DNA origami. Negative-stain transmission electron microscopy (TEM) imaging of the Rh and Rh-QD660/QR620 assemblies validated the assembly of the target DNA origami objects and different loading yields of Rh-QD660/QR620 assemblies using dQDs/dQRs and mQDs/mQRs (figs. S13 to S17). We found that the loading yields increased from 30 to 95% and from 36 to 78% for Rh-QR620 assemblies and Rh-QD660 assemblies, respectively, by using dQDs/dQRs instead of mQDs/mQRs (Fig. 4). Moreover, dQR620 could be aligned (within 30°) along 6HB edges of Rh with high fidelity (86%), which is a substantial improvement compared with mQR620 (14%) (Fig. 4A and fig. S18). We also noticed that a high proportion (14%) of Rh-dQD660 dimer (two DNA origami objects binding to a single QD) still formed even when Rh was incubated with 10-fold excess dQD660 (Fig. 4B and fig. S18), which may be explained by the highly efficient hybridization ability of dQD660, because the high-density ssDNA binding sites on dQD660 can still bind other DNA origami even after formation of QD-DNA origami assemblies. The quantum yields of Rh-dQD660 and Rh-dQR620 were 0.17 ± 0.02 and 0.47 ± 0.02, respectively, which were slightly lower than dQD660 (0.19 ± 0.03) and dQR620 (0.56 ± 0.04).

### SALSA and aligned QD/QR 2D arrays

**Fig. 5.**
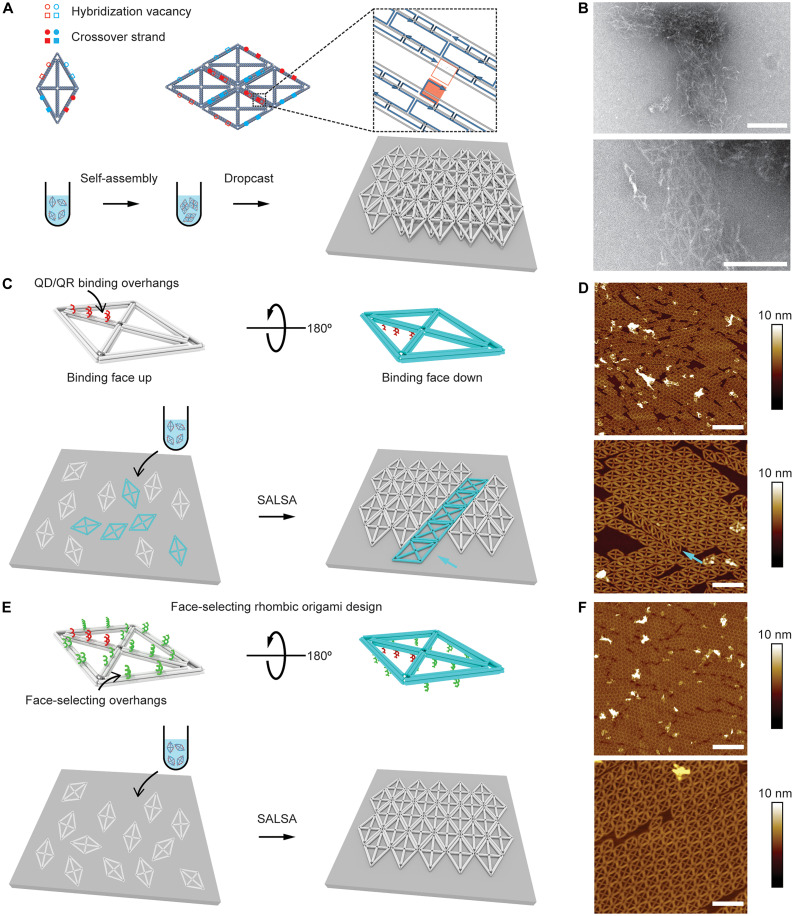
Crossover design and SALSA of rhombic origami. (**A**) Schematic of crossover design on a Rh for 2D lattice superstructures. Two 8-nt extensions are introduced to two neighboring edges respectively (solid squares and circles) with unique DNA sequences that hybridize to the two vacancies introduced in their parallel edges (hollow squares and circles). (**B**) TEM images showing aggregation and layered species of 2D origami lattice sample prepared via dropcast post assembly in solution. (**C**) Schematic and (**D**) dry AFM images of SALSA without face-selecting Rh. Lateral crossover strands are omitted from all illustrations. Origamis can land on the mica substrate facing up (white) or down (cyan). Each species assembles into separate lattices. Origami arrays landing on different sides are indicated with an arrow. (**E**) Schematic and (**F**) dry AFM images of SALSA with the face-selecting Rh. Face-selecting overhangs are introduced to the side of the binding strands to avoid binding strand facing down on mica. Face-selecting Rh only assembles into 2D lattices facing up. Scale bars, 600 nm [(D) and (F), top], 200 nm (B), and 200 nm [(D) and (F), bottom].

Having demonstrated excellent colloidal stability and hybridization ability of dQDs/dQRs, we next sought to develop a strategy to fabricate QD/QR 2D arrays. Here, we used Rh to demonstrate a SALSA strategy to fabricate DNA origami lattices directly on a solid substrate with control over relative origami orientation. The key to achieve relative orientational control of origami tiles within a 2D lattices is to introduce anisotropic lateral interactions between neighboring origamis (*66*–*68*). We realized this by designing DNA overhangs that can hybridize to a specific vacancy on an adjacent origami. Specifically, two neighboring edges of the origami are each designed with two crossover strands (solid circles and squares) with unique sequences that are complementary to their parallel counterparts with two hybridization vacancies (hollow circles and squares) (Fig. 5A). Although there are two modes of tiling for a rhombic geometry, hexagonal and orthorhombic (fig. S19), the 2D lattice will only be thermodynamically stable when the designed crossovers hybridize to their corresponding vacancies, directing the formation of the hexagonal lattice specifically. The assembly of this extended hexagonal lattice can be carried out in solution through thermal annealing (Fig. 5A), which, however, often results in layered structures or random aggregations during the sample deposition (here by drop cast) and drying steps (Fig. 5B). Hence, we used a surface-assisted method to directly assemble 2D lattices of our rhombic origami tiles with lateral crossovers on a mica surface. Monovalent cation (sodium) was used to tune the electrostatic interaction between the origami and the negatively charged substrate to allow on-surface diffusion of origami monomers and their coalescence when the lateral antiparallel crossovers match to generate a hexagonal lattice. Without the lateral crossover overhangs and vacancies, no lattice was formed (fig. S20). The intertile binding affinity can be tuned by the overhang length. We found that a 5-nt overhang only yielded small origami arrays (fig. S20), whereas an 8-nt overhang promoted the formation of micron-sized lattices (fig. S21).

We discovered three synergistic effects that are key to the formation of large origami lattices: (i) Na^+^ mediated SALSA where the monovalent cation promotes the diffusion and assembly of origami tiles; (ii) thermal annealing to break misassembled origami tiles for error correction; and (iii) controlling the same side of the origami landing on the substrate. Without or with a low concentration (<100 mM) of Na^+^, the affinity of the origami to the mica surface was so high that the origami tiles could not diffuse to form ordered lattices once they attached to the surface (fig. S22). Without sufficient thermal annealing, only small 2D arrays were observed due to misassembled origami tiles (fig. S23). Heating at a higher temperature (>60°C) will start to denature DNA origami and lower the surface coverage (fig. S23). Longer annealing times also help to produce large 2D origami lattices to some extent (fig. S24). We found that the conditions described in Methods yielded the best 2D lattices observed thus far.

Another key factor to fabricating large 2D origami lattices was to ensure that all origami tiles landed on the substrate in the same orientation. As shown in Fig. 5C, five QR binding strands (red) were introduced along the long axis of the wireframe rhombic origami, whose side was denoted as the top or the binding face. When depositing solution-based 2D origami structures onto a substrate, both the top (gray) and the bottom (cyan) can land on the surface, exposing or hiding the binding strand for the QR, respectively (Fig. 5C). Apart from the fact that the binding strands need to be exposed for subsequent functional material binding to the origami, origami tiles with different faces landing on the surface cannot form 2D lattices together due to their fully anisotropic crossover design, limiting the growth of the 2D lattices to relatively smaller sizes (Fig. 5D and fig. S25). We tackled this challenge by introducing 31 additional face-selecting 20-nt ssDNA overhangs (20 thymidine, green) to the binding face (Fig. 5E), which acted as entropic brushes that interfered with origami binding to the substrate. It is worth noting that the ssDNA entropic brush method was previously believed to be ineffective to bias selected origami face binding to mica due to the strong DNA-mica affinity (*29*). However, we discovered that with Na^+^ mediating the binding affinity and possibly the elevated temperature promoting dynamics, this method was able to bias the nonbinding face of the origami landing on the mica surface (Fig. 5F and fig. S21). As a result, larger 2D lattices of origami tiles with all binding faces oriented upward could be fabricated on the surface directly through SALSA (Fig. 5F).

**Fig. 6.**
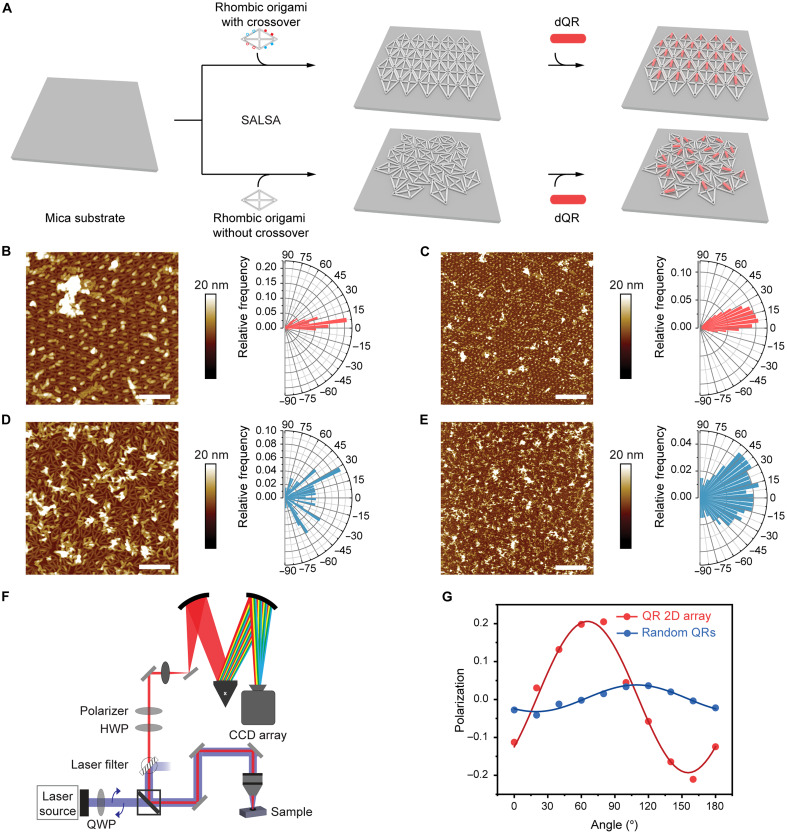
Orientation and polarization of 2D QR arrays. (**A**) Schematic of fabrication of aligned QR 2D array using Rh with and without crossovers. A 1-μm^2^ (**B**) or 9-μm^2^ (**C**) area AFM image (left) of QR 2D arrays using Rh with crossovers and their QR orientation distributions (right). A 1-μm^2^ (**D**) or 9-μm^2^ (**E**) area AFM image (left) of QR 2D array using Rh without crossovers and their QR orientation distribution (right). (**F**) Schematic of the polarization measurement setup. A quarter-wave plate (QWP) makes the 405-nm laser light circularly polarized which excites the sample. After removing laser light via filtering, the QR emission is analyzed with a half-wave plate (HWP) and linear polarizer, and the resulting emission spectrum is measured with a monochromator grating and charge-coupled device (CCD) camera. (**G**) The polarization (dot) and fitting curve (solid line, sinusoidal function) of the QR 2D array (red) and random QRs (blue) as a function of the polarizer angle. Scale bars, 200 nm [(B) and (D)] and 600 nm [(C) and (E)].

The preceding demonstration of 2D origami lattices provides a template for programmed QD/QR 2D arrays. To test our ability to control the positioning and alignment of QD/QR 2D arrays, we assembled dQD600 and dQR620 to preformed 2D origami lattices on surface (Fig. 6A and fig. S26). Atomic force microscopy (AFM) images showed excellent overall loading efficiency (>90%) of QDs/QRs onto SALSA origami lattices, presumably due to the highly efficient hybridization of dQDs/dQRs (Fig. 6, B and C, and fig. S26). The height profile of AFM images showed successful interparticle distance control (fig. S26). In contrast, we also assembled dQRs to SALSA lattices without face selecting overhangs (fig. S27). We observed a distinct binding behavior where for some lattice grains dQR bound to most origami units while no binding was observed for some other grains. We believe that the origami lattice grains with no dQR binding are the ones landed with the binding face down and thus hiding their binding strands (fig. S27). This result also further confirmed our effective face selection strategy by introducing ssDNA as steric brushes. To further enhance the alignment of dQR arrays, we compared two modes of hybridizing dQRs to 2D DNA origami lattices: a shear-like geometry and a zipper-like geometry (fig. S28). Orientation analysis from the AFM images showed that 67 ± 5% (mean ± SD; *n* = 3) and up to 73% of QRs were distributed within 30° in a 1-μm^2^ area using the zipper-like hybridization mode (Fig. 6B, and figs. S29 and S30); whereas in the case of the shear-like hybridization mode, only 58 ± 3% and up to 62% of QRs were distributed within 30° in a 1-μm^2^ area (fig. S31). This result shows that the zipper-like geometry can form rigid binding by reducing the distance between QRs and DNA nanostructures, which is consistent with results from previous reports (*69*–*71*). For a larger area of 9 μm^2^, the percentage of QRs aligned within 30° reduced to 56 and 50% for zipper-like geometry (Fig. 6C) and shear-like geometry (fig. S31) respectively, due to limited origami lattice sizes. As a negative control experiment, we performed SALSA using a 2D origami template without crossover overhangs (Fig. 6A). In this case, origami templates were randomly arranged on mica without lattices, hence orientation analysis of templated QRs showed much broader angle distributions (Fig. 6, D and E, and fig S29). To further characterize the QRs on the SALSA template, we measured the polarization dependence of the exciton emission (Fig. 6F). The degree of polarization (Eq. 5) for the 2D origami lattices templated QRs was measured to be 0.19 ± 0.03, compared with 0.04 ± 0.02 for a control sample of QRs without 2D origami lattices template (Fig. 6G and fig. S32). This higher degree of polarization indicated a greater spatial alignment of QRs, as the emission angular polarization depends on QR dipolar and spatial orientation (*72*, *73*). We assigned the nonzero degree of polarization for the negative control to a small local orientational alignment on the surface due to the random packing of QRs. The degree of polarization value reported here is comparable to what was achieved with a classical Langmuir-Blodgett method (~0.21) (*16*). We also theorize that the degree of polarization of our origami templated QR array can be further improved by optimizing the QR binding step to reduce QR aggregations (unaligned) on the origami lattice. In addition to the orientation control, our strategy is capable of prescribing the interparticle spacing of QRs with nanometer scale precision within an array, which cannot be realized by existing alignment methods.

## DISCUSSION

We demonstrated an ultrafast strategy to prepare high-density DNA-conjugated QDs/QRs directly from organic solution using a dehydration and rehydration process, which reduced manufacturing time from a few days to a few minutes. This method was examined for various QDs/QRs and dehydration conditions. We found experimentally that Na^+^ salt, dehydration volume ratio, and initial surface ligands were important parameters to consider for high-density DNA conjugation. We showed that these dQDs/dQRs had high hybridization efficiency and colloidal stability in salted aqueous buffer and could be assembled on DNA origami with high fidelity. Moreover, we developed the SALSA method to construct 2D origami lattices directly on a solid substrate to template QD and QR 2D arrays. Sodium ion concentration, thermal annealing, and a face-selecting strategy were essential to the fabrication of micron-sized origami lattice templates with uniformly controlled spacing and orientation for the binding and alignment of nanoparticles. Our dehydration-assisted approach tremendously simplifies and shortens the manufacturing time required for DNA-functionalized QDs/QRs, as well as increases the DNA density per QDs/QRs up to 10 times, compared with previous methods. SALSA circumvents the problematic issue of transferring solution-assembled structures onto solid substrates and further maintains lattice structures together with the templated nanomaterial arrangement and function after drying. The combination of these advances offers a generalized approach to fabricating 2D origami lattice–templated QD/QR arrays with precise spacing and orientation control.

In the current implementation, 2D origami lattices were limited in size (~1 μm^2^) due to uncontrolled nucleation of origami lattices on the surface with random initial orientations and positions. Future work might extend our approach using lithographically defined substrates to manipulate the initial nucleation steps of SALSA. The flexible and accurate angle control of the 6HBs in wireframe origami structures will also enable us to precisely tune the orientation of anisotropic functional materials like nanorods and explore their arrangement-dependent properties. Together, the use of dehydration-assisted DNA conjugation and SALSA is expected to facilitate the translation of nanoscale DNA origami design strategies into surface nanofabrication, where scalable production and nanoscale precision are essential to achieve target device performance.

## MATERIALS AND METHODS

### General materials

CdSe/ZnS core/shell QDs [catalog numbers: 900218 (QD600) and 900249 (QD660)], CdSe/CdS core-shell type QRs [catalog numbers: 900512 (QR560) and 900514 (QR620)], MPA (≥99%, catalog number: M5801), mPEG [≥95% (oligomer purity), catalog number: 672572), TOPO (ReagentPlus, 99%, catalog number: 223301), TBAB (ACS reagent, ≥98.0%, catalog number: 426288), and tris(2-carboxyethyl) phosphine hydrochloride solution (TCEP) [(pH 7.0) catalog number: 646547] were purchased from Sigma-Aldrich. Basic Agarose (catalog number: IB70070) was purchased from IBI Scientific. TBE 10× buffer [(pH 8.3) catalog number: 1610733] was purchased from Bio-Rad Laboratories Inc. TAE 10× buffer [(pH 8.3 ± 0.1) ribonuclease/deoxyribonuclease and protease free, catalog number: 46-010-CM] and phosphate-buffered saline (PBS) 1× buffer [(pH 7.4 ± 0.1) without calcium and magnesium, catalog number: 21-040-CM] were purchased from Corning. All DNA oligonucleotides were purchased from Integrated DNA Technologies (IDT; Coralville, IA) with standard desalting or high-performance liquid chromatography (for dye-modified DNA oligonucleotides) as purification method. Thiol-modified oligos ordered from IDT are shipped in their oxidized form, with the sulfur atoms protected by an S─S bond (Mod Code: /5ThioMC6-D/ or /3ThioMC3-D/), and were reduced using TCEP in 100× excess. All DNA oligonucleotides were received as dry pellets. Sodium chloride (5 M, catalog number: AM9760G), MgCl_2_ (1 M, catalog number: AM9530G), and tris [1 M (pH 8.0), catalog number: AM9855G] were purchased from Life Technologies Corporation DBA Invitrogen. Mica discs (V1 quality, highest grade, 0.21-mm thickness, catalog number: 50-12) were purchased from Ted Pella Inc. and used for all AFM imaging.

### Dehydration-assisted high-density DNA-conjugated QDs/QRs (dQDs/dQRs)

dQDs/dQRs were prepared using dehydration-assisted directly phase transfer from commercial organic QDs/QRs. Briefly, 5 μl of QD (5 mg/ml; QD600, QD660, QR560, and QR620 in toluene or hexane or chloroform) was incubated with thiolated ssDNA (21 nt) (sequence: 5′-thiol-AAA AAA AAA CCC AGG TTC TCT-3′) at a desired molar ratio (200:1 for QD600; 500:1 for QD660; 200:1 for QR560; 500:1 for QR620) in the presence of 100 mM NaOH or NaCl to reach a final volume of 50 μl. Such solution was sonicated at 37 Hz around 5 min and then immediately combined with 600 μl of 1-butanol followed by a quick vortex for several seconds. Subsequently, 200 μl of 0.5× TBE buffer was added to the above solution followed by another quick vortex and a brief centrifugation at 2000*g* for several seconds to facilitate a liquid phase separation. DNA-functionalized QDs/QRs were then recovered as a sublayer of the resulting two immiscible liquids. To remove excess ssDNA, DNA-functionalized QDs/QRs were purified and concentrated using an ultracentrifugal filter (Amicon 100 kDa) five times at 8000*g* for 3 min for each centrifugation step. The whole process was carried out under ambient conditions, assisted only by sonication, vortex mixing, and centrifugation-facilitated phase separation.

### DNA-conjugated aqueous QDs/QRs (mQDs/mQRs)

Aqueous QDs were prepared as described previously, with minor modifications (*30*, *58*). Briefly, 80 μl of QD (5 mg/ml; QD600, QD660, QR560, and QR620 in chloroform) was incubated with 160 μl of TOPO (1 g/10 ml in chloroform) and 160 μl of chloroform at 25°C and shaken at 1200 rpm using a thermal mixer. After 30 min, 20 μl of TBAB (0.3 M in chloroform) was added to this mixture. After an additional 30 min of incubation and shaking, 400 μl of MPA in NaOH (11 mM in 0.2 M aqueous NaOH) was added. The mixture was briefly vortexed and centrifuged at 2000*g* for several seconds, and the aqueous layer was recovered. The vortexing and centrifugation steps were repeated until all aqueous layers were collected. MPA-QDs were purified to remove excess MPA and concentrated using an ultracentrifugal filter (Amicon 30 kDa) six times at 8000*g* for 5 min for each centrifugation step. After purification, the MPA-QDs were diluted to 500 μl with nuclease-free water and incubated with 20 μl of mPEG for 4 days at room temperature. The MPA/mPEG-QDs were purified and concentrated using an ultracentrifugal filter (Amicon 30 kDa) six times at 8000*g* for 5 min and then buffer-exchanged into 10 mM tris using a NAP-5 desalting column (GE Healthcare). Aqueous QDs were incubated with thiolated-ssDNA at desired molar concentration for conjugation (200:1 for QD600; 500:1 for QD660; 200:1 for QR560; 500:1 for QR620). Last, purified DNA-conjugated QDs/QRs concentrations were determined by measuring the absorbance of samples at 350 nm.

### DNA-conjugated aqueous QDs using dehydration and rehydration process (mdQDs)

Aqueous QD660 was prepared as described above. mdQD660 was prepared using dehydration-assisted DNA condense from aqueous QD660. Briefly, 5 μl of QD660 (100 nM) was incubated with thiolated ssDNA (51 nt) (sequence: 5′-thiol-AAA AAA AAA AAA AAA AAA AAA AAA AAA AAA TTG TGA CAG CTG GAT CGT TAC-3′) at a desired molar ratio (500:1) in the presence of 100 mM NaOH to reach a final volume of 50 μl. Such solution was sonicated at 37 Hz around 5 min and then immediately combined with 600 μl of 1-butanol followed by a quick vortex for several seconds. Subsequently, 200 μl of 0.5× TBE buffer was added to the above solution followed by another quick vortex and a brief centrifugation at 2000*g* for several seconds to facilitate a liquid phase separation. DNA-functionalized QD660 were then recovered as a sublayer of the resulting two immiscible liquids. To remove excess ssDNA, DNA-functionalized QD660 were purified and concentrated using an ultracentrifugal filter (Amicon 100 kDa) five times at 8000*g* for 3 min for each centrifugation step.

### Effect of NaOH, TOPO, and TBAB

To investigate the impact of NaOH, TOPO, and TBAB on DNA density per QD, dQD660 was incubated with thiolated DNA (51 nt) (sequence: 5′-thiol-AAA AAA AAA AAA AAA AAA AAA AAA AAA AAA TTG TGA CAG CTG GAT CGT TAC-3′) (case 1), or combined with NaOH (case 2), or NaOH and TOPO (case 3), or NaOH and TBAB (case 4), or NaOH, TOPO, and TBAB (case 5) before sonication. In a typical experiment, for above case 1, 20 μl of thiolated ssDNA was added to 5 μl of octadecylamine capped QD660 at a molar ratio of 500:1 to reach a final volume of 50 μl. For above case 2, 20 μl of thiolated ssDNA was added to 5 μl of octadecylamine capped QD660 at a molar ratio of 500:1 in the presence of 100 mM NaOH to reach a final volume of 50 μl. For above case 3, 5 μl of octadecylamine capped QD660 was incubated with 5 μl of TOPO (1 g/10 ml) for 30 min, and 20 μl of thiolated ssDNA was added to above mixture at a molar ratio of 500:1 in the presence of 100 mM NaOH to reach a final volume of 50 μl. For above case 4, 5 μl of octadecylamine capped QD660 was incubated with 2 μl of TBAB (0.3 M) for 30 min, and 20 μl of thiolated ssDNA was added to above mixture at a molar ratio of 500:1 in the presence of 100 mM NaOH to reach a final volume of 50 μl. For above case 5, 5 μl of octadecylamine capped QD660 was incubated with 5 μl of TOPO (1 g/10 ml) and 2 μl of TBAB (0.3 M) for 30 min, and 20 μl of thiolated ssDNA was added to above mixture at a molar ratio of 500:1 in the presence of 100 mM NaOH to reach a final volume of 50 μl. Such solution was sonicated around 5 min and then was immediately combined with 600 μl of 1-butanol followed by a quick vortex for several seconds. Subsequently, 200 μl of 0.5× TBE buffer was added to the above solution followed by another quick vortex and a brief centrifugation at 2000*g* for several seconds to facilitate a liquid phase separation. DNA-functionalized QD660 were then recovered as a sublayer of the resulting two immiscible liquids. DNA-functionalized QD660 sample without purification (15 μl) was combined with 3 μl of 6× loading buffer (New England Biolabs) and loaded to a 1% agarose gel with 0.5× TBE. Each gel was run at 65 V for 60 min in 0.5×TBE at 4°C. Gels were then visualized under blue light transilluminator.

### Effect of dehydration volume ratio

To investigate the impact of dehydration volume ratio on DNA density per QD, different 1-butanol/water volume ratio was used to prepare the dQD660 during dehydration process. In a typical experiment, thiolated ssDNA (51 nt) (sequence: 5′-thiol-AAA AAA AAA AAA AAA AAA AAA AAA AAA AAA TTG TGA CAG CTG GAT CGT TAC-3′) was added to octadecylamine capped QD660 at a molar ratio of 500:1 in the presence of 100 mM NaOH to reach a final volume of 50 μl. Such solution was sonicated around 5 min and then was immediately combined with 50, 300, 450, 600, or 750 μl of 1-butanol followed by a quick vortex for several seconds. Subsequently, 20, 100, 150, 200, or 250 μl of 0.5×TBE buffer was added to the above solution followed by another quick vortex and a brief centrifugation at 2000*g* for several seconds to facilitate a liquid phase separation. DNA-functionalized QD660 were then recovered as a sublayer of the resulting two immiscible liquids. DNA-functionalized QD660 sample without purification (15 μl) was combined with 3 μl of 6× loading buffer (New England Biolabs) and loaded to a 1% agarose gel with 0.5× TBE. Each gel was run at 65 V for 60 min in 0.5× TBE at 4°C. Gels were then visualized under blue light transilluminator.

### Effect of dehydration time

Various dehydration time was tested for dQD660 preparation. In a typical experiment, thiolated ssDNA (51 nt) (sequence: 5′-thiol-AAA AAA AAA AAA AAA AAA AAA AAA AAA AAA TTG TGA CAG CTG GAT CGT TAC-3′) was added to octadecylamine capped QD660 at a molar ratio of 500:1 in the presence of 100 mM NaOH to reach a final volume of 50 μl. Such solution was sonicated around 5 min and then was immediately combined with 600 μl of 1-butanol followed by a quick vortex for several seconds. After 0, 30, 60, or 90 min of incubation and shaking, 200 μl of 0.5×TBE buffer was added to the above solution followed by another quick vortex and a brief centrifugation at 2000*g* for several seconds to facilitate a liquid phase separation. DNA-functionalized QD660 were then recovered as a sublayer of the resulting two immiscible liquids. DNA-functionalized QD660 sample without purification (15 μl) was combined with 3 μl of 6× loading buffer (New England Biolabs) and loaded to a 1% agarose gel with 0.5× TBE. Each gel was run at 65 V for 60 min in 0.5× TBE at 4°C. Gels were then visualized under blue light transilluminator.

### Quantify the DNA density per QD/QR

To quantify DNA density per QD/QR, 5′-thiolated DNA with an extra FAM at the 3′ terminus (21 nt) (sequence:5′-thiol-AAA AAA AAA CCC AGG TTC TCT-FAM-3′) was used to prepare DNA-conjugated QD/QR. The concentrations of QDs/QRs were obtained by ultraviolet-visible (UV-vis) extinction spectroscopy with diameter-dependent extinction coefficients calculated from an empirical equation (*74*–*76*). The actual sizes of the QDs and QRs were determined by TEM. Correspondingly, the following extinction coefficients at 350 nm were used for determining the molar concentrations of 6-nm QD, 14-nm QD, 4/16-nm (diameter/length) QR, and 5/29-nm QR: 3.0 × 10^6^ M^−1^ cm^−1^, 2.9 × 10^7^ M^−1^ cm^−1^, 2.3 × 10^7^ M^−1^ cm^−1^, and 6.4 × 10^7^ M^−1^ cm^−1^. DNA concentration was determined by FAM fluorescence calibration curve, where fluorescence was excited at 485 nm with emission recorded from 500 to 700 nm. For comparison, DNA-functionalized QDs/QRs (mQDs/mQRs) were also prepared by a method as described above.

### Hybridization ability of mQDs/mQRs and dQDs/dQRs

To fabricate mQD600-Cy5, dQD600-Cy5, mQD660-Cy5, dQD660-Cy5, mQR560-Cy5, dQR560-Cy5, mQR620-Cy5, and dQR620-Cy5 FRET pairs, mQD600, dQD600, mQD660, dQD660 mQR560, dQR560, mQR620, and dQR620 were incubated with twofold excess complementary DNA with a Cy5 modifier at the 3′ terminus (sequence: 5′-AGA GAA CCT GGG-Cy5–3′) in PBS buffer. After 2-hour incubation, the fluorescence emission spectra of QDs/QRs alone and in the presence of Cy5 were recorded. To fabricate the QD/QR-DNA origami assemblies, wireframe rhombic origami with binding overhangs was incubated with mQD660, dQD660, mQR620, and dQR620 at room temperature overnight, respectively (molar ratio was 1:4 and 1:10 for QRs and QDs, respectively, in 1× TAE with 20 mM MgCl_2_).

### DNA origami folding

The 6HB wireframe rhombic origami was folded using a reported method (*44*) in a tris buffer containing 40 mM tris and 12.5 mM MgCl_2_ with a pH adjusted to 8.3 ± 0.2 (1× TMg). One hundred microliters of 100 nM DNA scaffold (M13mp18/p7249, *Tilibit nanosystems*) was mixed with 20 equivalent all corresponding staple strands, and the final buffer condition was adjusted to in 1× TMg. The final concentration of the scaffold was about 20 nM. The mixed solution was annealed in a polymerase chain reaction thermocycler: 95°C for 5 min, 85°C down to 76°C for 5 min/°C, 75°C down to 30°C for 13.75 min /0.5°C, 29°C down to 25°C for 10 min/°C, and then held at room temperature. The crude origami sample was directly used for SALSA without further purification. All DNA sequences are summarized in tables S6 and S7.

### Surface-assisted large-scale assembly

In a typical SALSA process, the as-synthesized origami was mixed with a concentrated NaCl solution (5 M) for a 1.5-ml solution with a final origami concentration of 500 pM and a Na^+^ concentration of 0.5 M. This mixture was added to a well on a 24-well microplate, and a freshly cleaved mica disc (*D* = 12 mm) was placed on top of the liquid surface in the well, floating with the cleaved side in contact with the solution surface. Then, the microplate was sealed and placed on a hotplate shaker (BioShake iQ, QInstruments) for 12 cycles of heating at 60°, 55°, and 50°C for 1 hour each (36 hours in total) with 200 rpm shaking and then let the setup naturally cool down to room temperature. Note that heating temperatures above were instrument settings, and actual sample temperatures were measured to be 50°, 47°, and 44°C, respectively. The mica disc was then taken out of the microplate well and carefully rinsed with 100 μl of 1× TMg buffer (with 0.5 M Na^+^) 10 times, with 1× TMg buffer (without Na^+^) 6 times, and with 1× TNi (40 mM tris and 12.5 mM MgCl_2_ with a pH adjusted to 8.3 ± 0.2) 3 times before incubating with 50 μl 1× TNi on the disc for 5 to 10 min. After the incubation, the disc was rinsed with 100 μl of Milli-Q water three times and dried with compressed air. Next, the mica disc was kept under vacuum for at least 1 hour before AFM imaging. For QD/QR binding experiments, the SALSA sample on the mica disc after annealing was only rinsed 12 times with 100 μl of 1× TMg buffer (with 0.5 M Na^+^) and kept wet before the next step. Iterations of the synthesis method for optimization were noted with the results in the Supplementary Materials.

### QD/QR binding to SALSA 2D origami lattice and orientation analysis

After rinsing, SALSA origami lattices on a mica disc were placed on the liquid surface of 1 ml of 1× TMg buffer (with 0.5 M Na^+^) containing 1 nM dQDs/dQRs in a well of a 24-well microplate and incubated for 4 hours with 200 rpm shaking. The mica disc was then taken out of the microplate well and carefully rinsed with 100 μl of 1× TMg buffer (with 0.5 M Na^+^) 10 times, with 1× TMg buffer (without Na^+^) 6 times, and with 1× TNi (40 mM tris and 12.5 mM MgCl_2_ with a pH adjusted to 8.3 ± 0.2) 3 times before incubating with 50 μl of 1× TNi on the disc for 5 to 10 min. After the incubation, the disc was rinsed with 100 μl of Milli-Q water three times and dried with compressed air. dQDs/dQRs were functionalized with a 5′-thiol–modified DNA (sequence: 5′-thiol-AAA AAA AAA CCC AGG TTC TCT-3′) for a “shear-like” hybridization or a 3′-thiolated DNA (sequence: 5′-CCC AGG TTC TCT AAA AAA AAA-thiol-3′) for a “zipper-like” hybridization. For orientation analysis of QR 2D arrays, only the angles of QR monomers in the AFM images were measured and counted in the analysis. OrientationJ (*77*, *78*), a plugin for the Fiji software package, was used to measure the angles of the QRs on origami lattice. Specifically, an AFM image was first processed using Fiji to extract shapes of the QRs by applying thresholds. Then, QR aggregations were removed either manually or by automated particle size analysis. Last, the angles of the remaining QRs were acquired by OrientationJ with either the *Distribution* function (9-μm^2^ images) or the *Measure* function (1-μm^2^ images) with the horizontal *x* axis as 0°.

### Microscopy and spectroscopic characterization

TEM characterization was carried out using a Thermo Fisher FEI Tecnai Spirit Transmission Electron Microscopy operating at 120 kV. For QDs with organic ligands, 10 μl of QDs (50 μg/ml) was drop casted on 400-mesh carbon film square grids (Thermo Fisher Scientific, catalog number: 5024891). For DNA origami and QD/QR-origami assemblies, 10 μl of wireframe DNA origami objects with or without attached QDs/QRs (5 nM) was adsorbed on glow-discharged 400-mesh carbon film square grids and stained by 2% aqueous uranyl formate solution containing 25 mM NaOH.

AFM measurements were performed under air condition in either on an Icon Atomic Force Microscope (Bruker) in ScanAsyst mode using a ScanAsyst-Air silicon tip on nitride lever (tip radius = 2 nm, *k* = 0.4 N/m, *f*_o_ = 70 kHz; Bruker) or on an Asylum Research Jupiter XR AFM (Oxford Instruments) in tapping mode using an ARROW-UHF ultrahigh-frequency probe (tip radius < 10 nm, *f*_o_ = 2000 kHz; NanoWord).

Absorbance spectra were measured using an Evolution 260 Bio UV-vis spectrophotometer (Thermo Fisher Scientific), and steady-state emission spectra (λ_ex_ = 450 nm) were measured using a multimode microplate reader (Tecan Spark). Quantum yields of QDs/QRs were determined using the relative quantum yield determination method with rhodamine 101 in spectroscopic-grade ethanol as standard (λ_ex_ = 480 nm, Φ_s_ = 0.92) (*79*).

### FRET calculations

The overlap integral (*J*) and Förster distance (*R*_0_) were calculated using Eqs. 1 and 2 (*80*).

$$J=\int {\bar{I}}_{D}(\lambda )\varepsilon_{A}(\lambda )\lambda^{4}d\lambda$$ (1)

where ${\bar{I}}_{D}(\lambda )$ is the area-normalized emission spectrum of the donor, ɛ_A_(λ) is the molar absorptivity spectrum of the acceptor in M^−1^ cm^−1^, and λ is the wavelength in nm (*80*).

$$R_{0}=0.0211{\left[\kappa^{2}{\text{Φ}}_{D}n^{-4}J(\lambda )\right]}^{\frac{1}{6}}\;(\text{in nm})$$ (2)

where κ^2^ is the orientation factor (assumed to be 2/3), Φ_D_ is the quantum yield of the donor, and *n *= 1.35 is the refractive index of the medium. The molar extinction coefficients for Cy5 were obtained from the suppliers.

FRET efficiencies were calculated using Eq. 3 (*80*),

$$E_{\mathrm{FRET}}=1-\frac{I_{DA}}{I_{D}}$$ (3)

where *I*_DA_ is the emission intensity of the QD-dye FRET pairs and *I*_D_ is the emission intensity of QD alone.

In the case of FRET from one QD to *n* equidistant dyes, the FRET efficiency can be calculated using Eq. 4 (*62*),

$$E_{\mathrm{FRET}}=\frac{n{R_{0}}^{6}}{n{R_{0}}^{6}+R^{6}}$$ (4)

### Polarization measurements and calculations

For polarization measurements, the prepared sample on a mica substrate is excited with a Coherent 405 nm continuous wave laser via a Nikon 0.80 numerical aperture 20× objective. For measuring emission polarization, the incoming laser light (which is naturally strongly linearly polarized) is circularly polarized with a quarter-wave plate (AQWP05M-600) to ensure uniform excitation. The emission is filtered with a Semrock BrightLine 409-nm longpass filter to remove the laser light, and then sample emission passes through a half-wave plate (HWP) (AHWP05M-600) and a wire-grid polarizer (WP25M-VIS). For acquiring energy spectra, the light was directed into a Princeton Instruments Acton spectrometer and a Princeton Instruments ProEM 512 × 512 charge-coupled device array. As the HWP is rotated, the change intensity is recorded by integrating the entirety of the spectral range (roughly 533 to 700 nm), as it passes through the parallel (perpendicular) polarizer, and the degree of polarization at each detection angle is calculated using Eq. 5 (*6*)

$$p=\frac{I_{\|}-I_{⊥}}{I_{\|}+I_{⊥}}$$ (5)

where *I*_||_ and *I*_⊥_ are the parallel and perpendicular intensities, respectively.

## Supplementary Materials

### This PDF file includes:

Figs. S1 to S32 Tables S1 to S7 References
