# Supplementary material for: Ultrafast dense DNA functionalization of quantum dots and rods for scalable 2D array fabrication with nanoscale precision
Source: Sci Adv. 2023 Aug 11;9(32):eadh8508. doi: 10.1126/sciadv.adh8508 (PMC10421044; doi:10.1126/sciadv.adh8508)
Supplement: Supplementary file 1 — Figs. S1 to S32 Tables S1 to S7 References [file sciadv.adh8508_sm.pdf]

Science Advances
AAAS

# Supplementary Materials for

## Ultrafast dense DNA functionalization of quantum dots and rods for scalable 2D array fabrication with nanoscale precision

Chi Chen *et al.*

Corresponding author: Mark Bathe, mark.bathe@mit.edu

*Sci. Adv.* **9**, eadh8508 (2023)
DOI: 10.1126/sciadv.adh8508

**This PDF file includes:**

Figs. S1 to S32
Tables S1 to S7
References

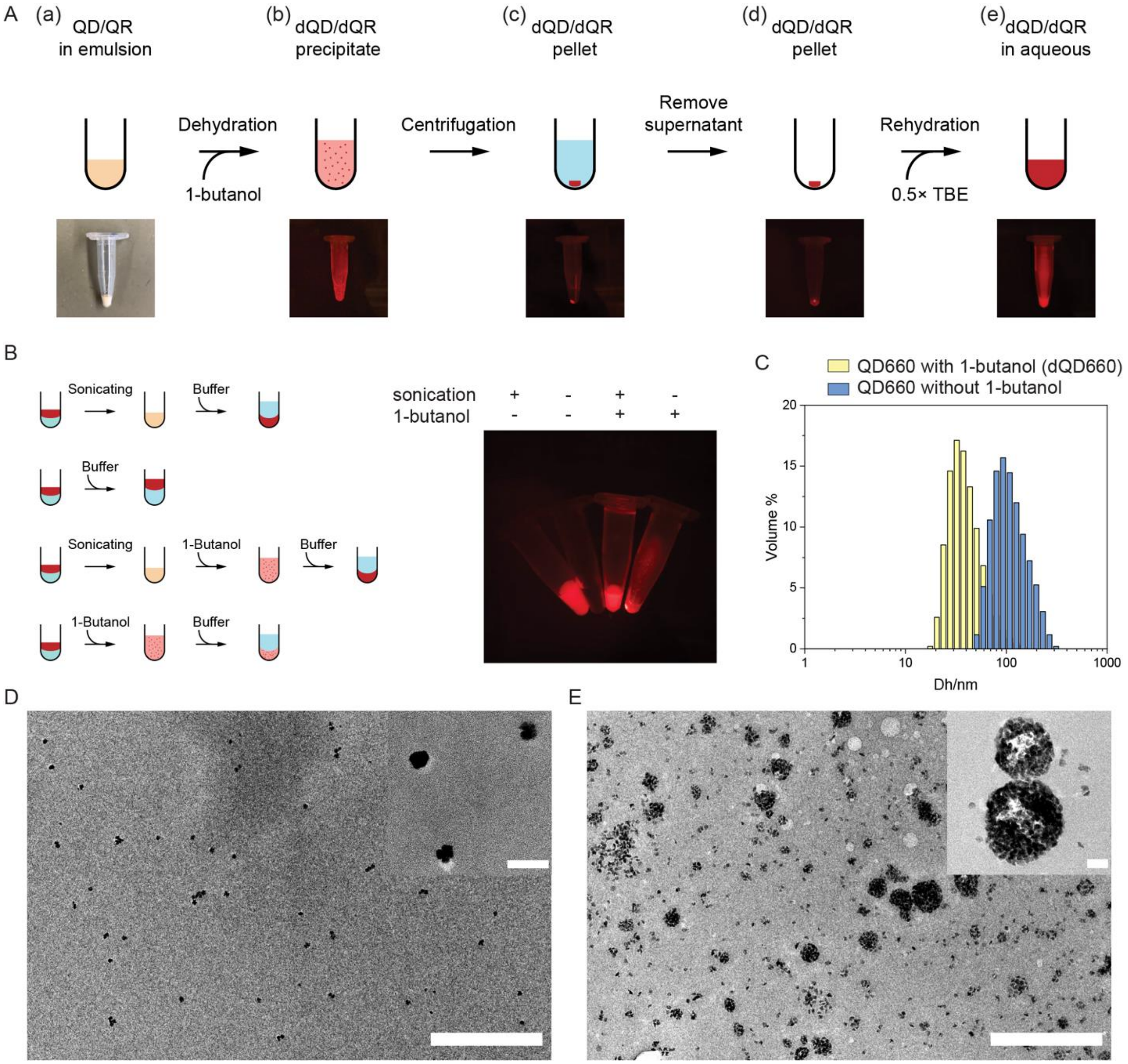


**Fig. S1.**

**Dehydration & rehydration process.** (**A**) Schematic and digital photo (under blue light) of the dehydration & rehydration process. (**B**) Schematic and digital photo (under blue light) for DNA conjugation to QD660 with and without sonication and 1-butanol dehydration (Schemes from top to bottom correspond to samples from left to right). (**C**) Hydrodynamic diameter (Dh) of as prepared QD660 with and without 1-butanol dehydration (both with sonication). Results showed that without 1-butanol dehydration, QDs formed aggregates consisting of more than 100 QDs in size assuming spherical aggregates. This aggregate size was confirmed by TEM images of QD660 with (**D**) and without (**E**) 1-butanol dehydration (both with sonication). Scale bars: 500 nm for (D) and (E); 50 nm for insets (D) and (E). The aggregation of QDs prevented accurate quantification of the DNA loading number on these assemblies due to changes in particle/dye absorption and/or fluorescence.

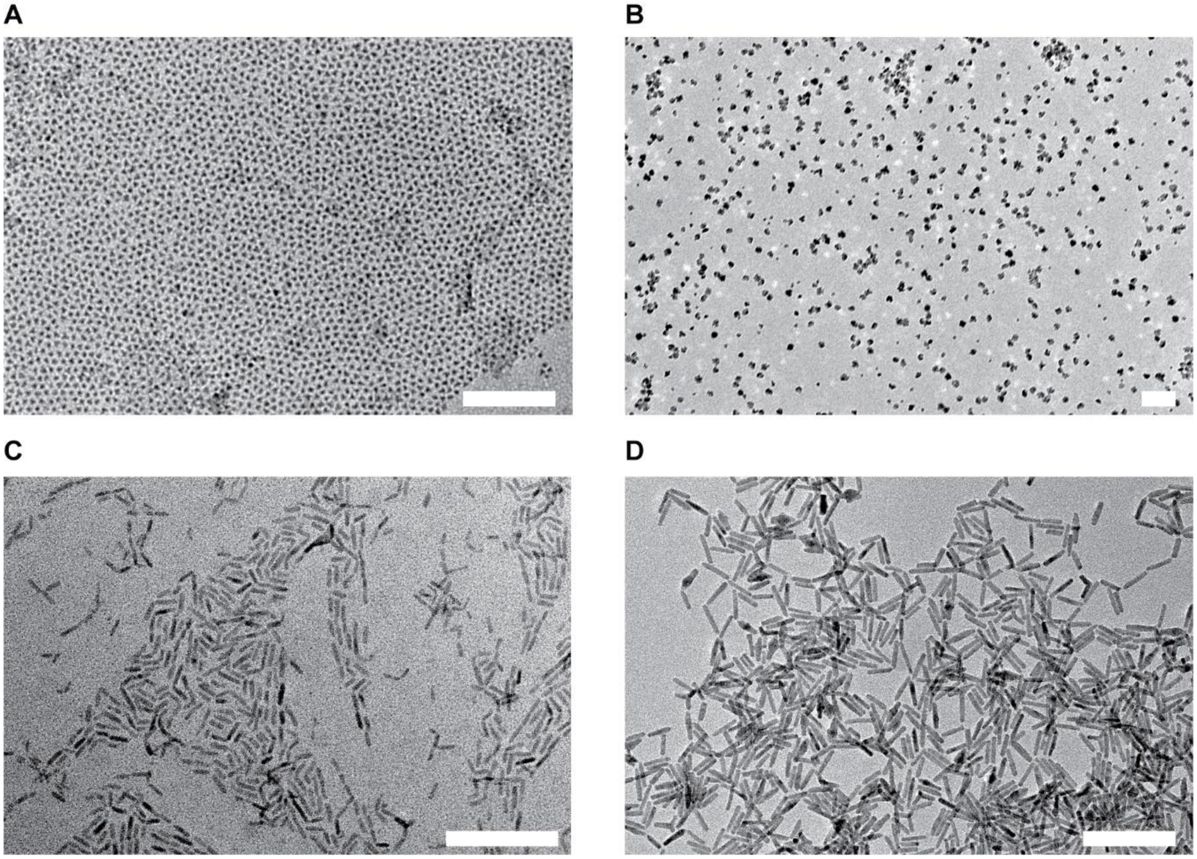


**Fig. S2.**

**TEM images of QDs and QRs in wide-field view.** TEM images of (**A**) QD600, (**B**) QD660, (**C**) QR560, and (**D**) QR620 in wide-field view (Scale bars: 100 nm).

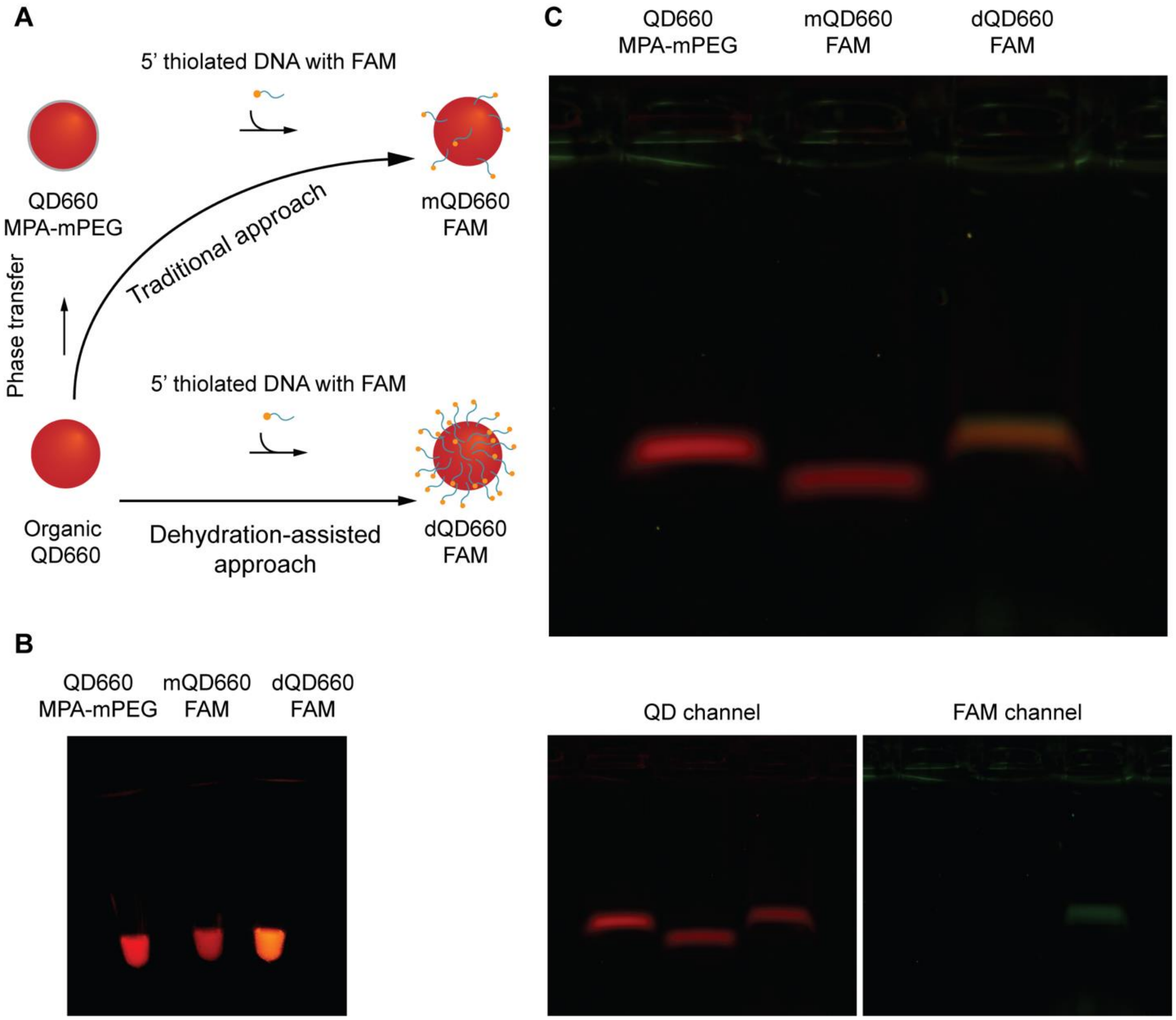


**Fig. S3.**

**Determining the DNA loading density using thiolated DNA with FAM.** (**A**) Schematic for preparation of mQD660 FAM and dQD660 FAM. (**B**) Digital photo of QD660 MPA-mPEG, mQD660 FAM, and dQD660 FAM in tube. Fluorescence images taken by digital camera (iPhone X) under blue light excitation. (**C**) AGE (1%) images of QD660 MPA-mPEG, mQD660 FAM, and dQD660 FAM. Red: QD channel, Green: FAM channel.

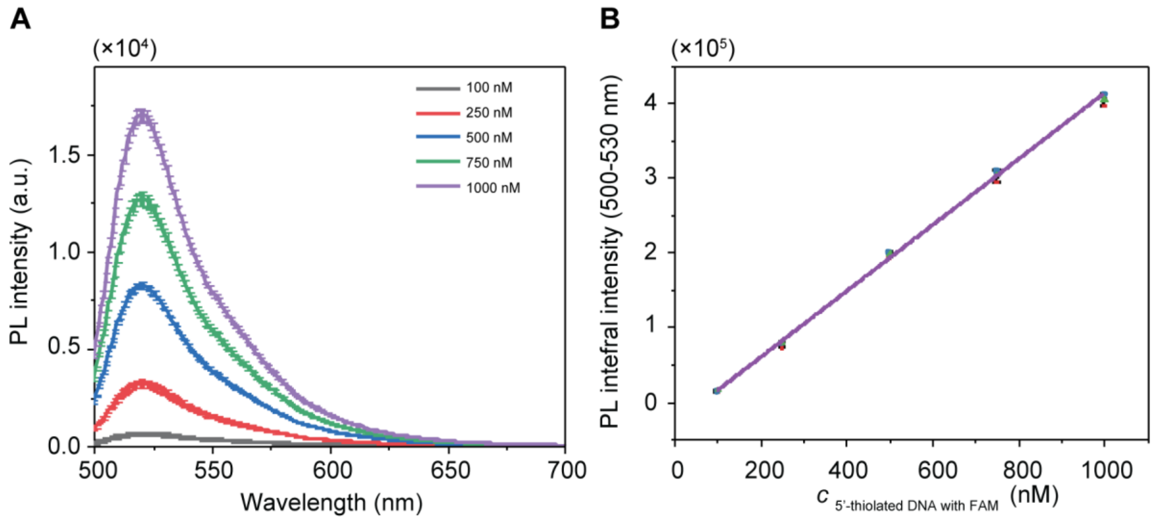


**Fig. S4.**

**Determining the DNA loading density using fluorescence calibration curve.** (**A**) Photoluminescence (PL) spectra of thiolated DNA labeled with FAM in various concentration. (**B**) FAM fluorescence calibration curve calculated from b) (x=(y+29450)/442, $R^2$=0.99916). FAM was excited at 485 nm.

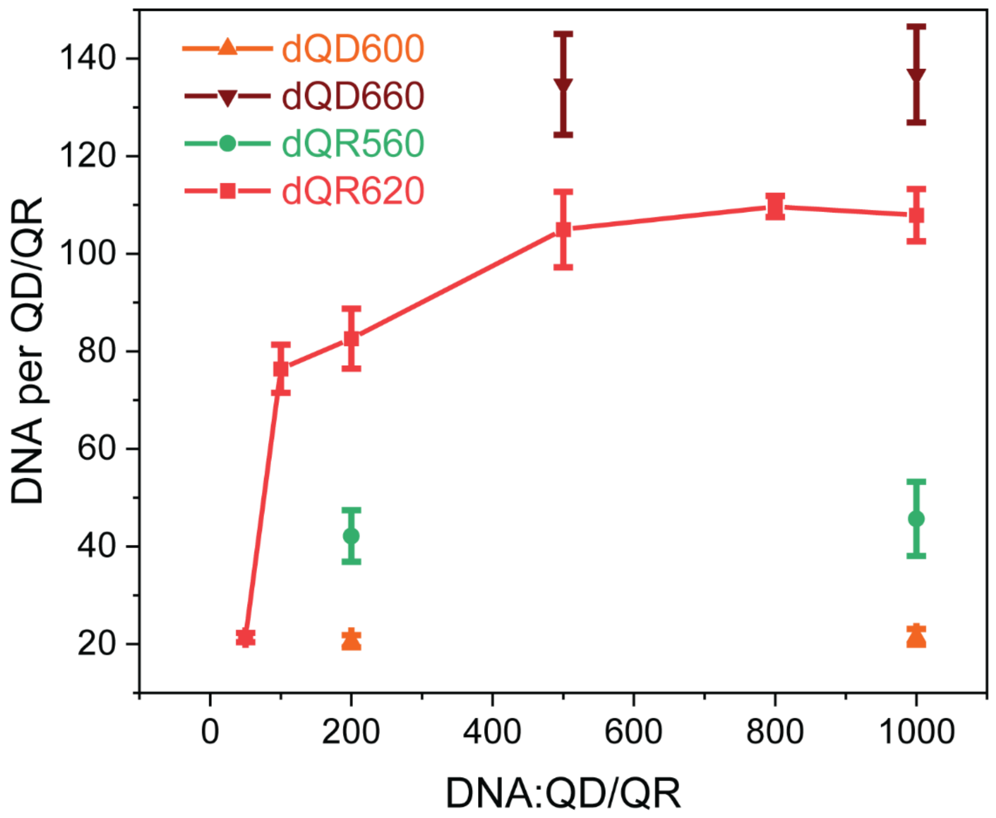


**Fig. S5.**

**DNA:QD/QR ratio effect on DNA loading density.** DNA per QD/QR using different DNA:QD/QR ratios (50, 100, 200, 500, 800, and 1000 for QR620, 200 and 1000 for QR560 and QD600, 500 and 1000 for QD660) for conjugation. Error bars represent standard deviations of the mean ($n$ = 3 replicates per group).

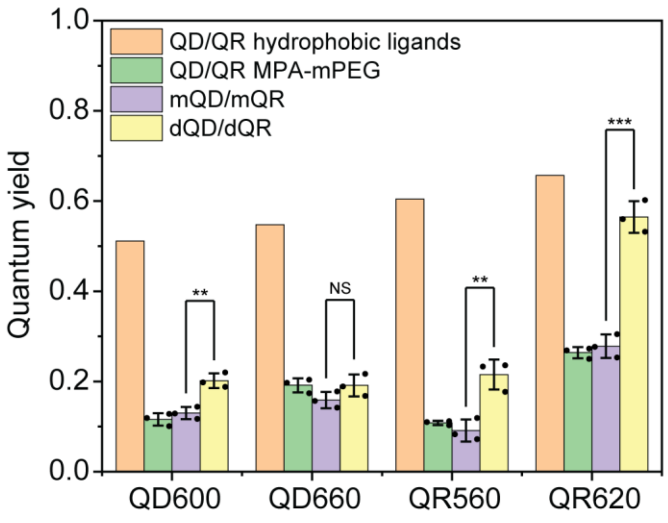


**Fig. S6.**

**Effects of distinct ligands and methods on quantum yield.** Quantum yield of QDs/QRs before and after conjugation using different methods. Error bars represent standard deviations of the mean ($n$ = 3 replicates per group). $P$-values are from Student's t-test (**$P < 0.01$; ***$P < 0.001$; NS $P \geq 0.05$).

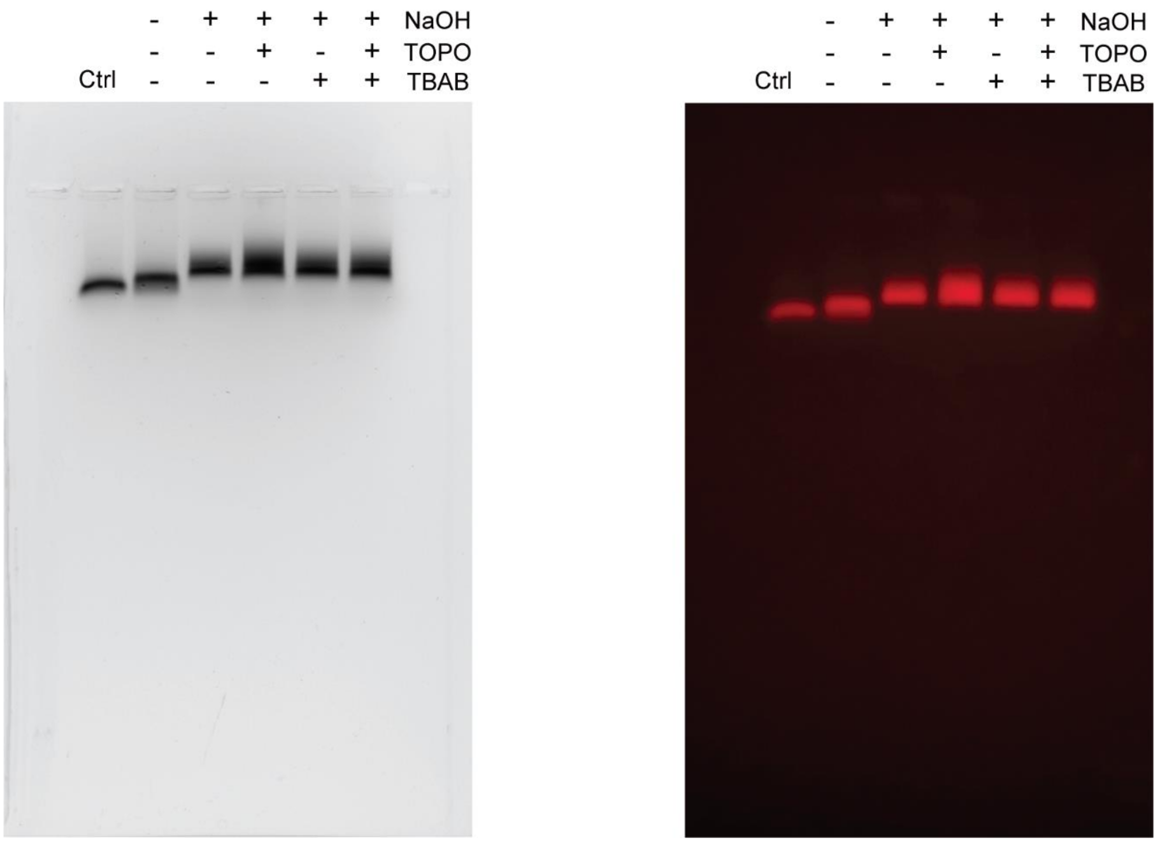


**Fig. S7.**

**NaOH, TOPO, and TBAB effect on DNA loading density.** AGE (1%) images of dQD660 prepared with or without NaOH, TOPO, and TBAB (Ctrl: mQD660). Fluorescence images taken by gel imaging system (left) and digital camera (iPhone X) under blue light excitation (right).

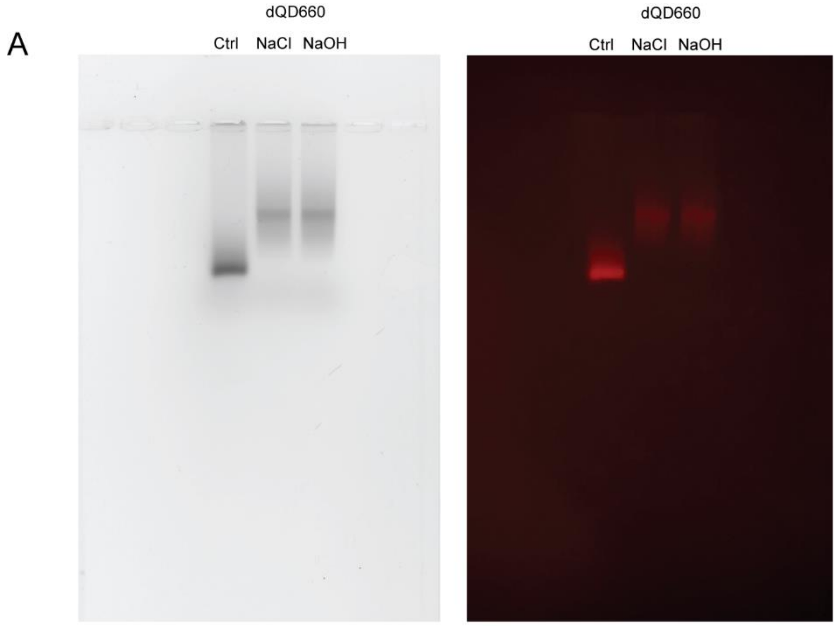


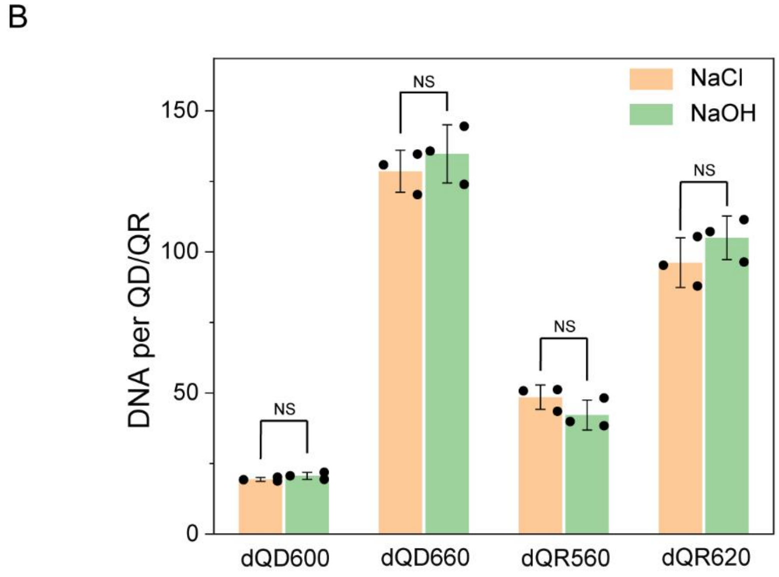


**Fig. S8.**

**Dehydration-assisted DNA conjugation using NaCl or NaOH.** (**A**). AGE (1%) images of dQD660 prepared using NaCl or NaOH (Ctrl: mQD660). Fluorescence images taken by gel imaging system (left) and digital camera (iPhone X) under blue light excitation (right). (**B**). DNA density per dQD/dQR using NaCl or NaOH. Error bars represent standard deviations of the mean ($n$ = 3 replicates per group). $P$-values are from Student's t-test (NS $P \geq 0.05$).

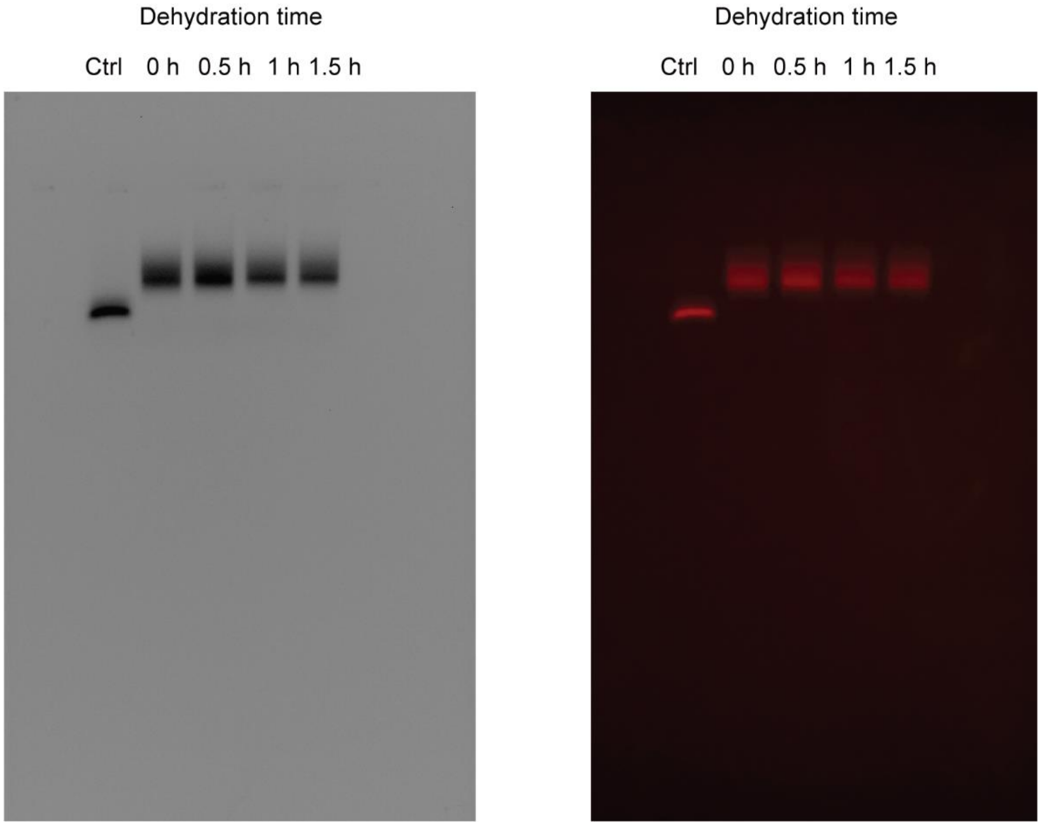


**Fig. S9.**

**Dehydration time effect on DNA loading density.** AGE (1%) images of dQD660 prepared with various dehydration time (Ctrl: mQD660). Fluorescence images taken by gel imaging system (left) and digital camera (iPhone X) under blue light excitation (right).

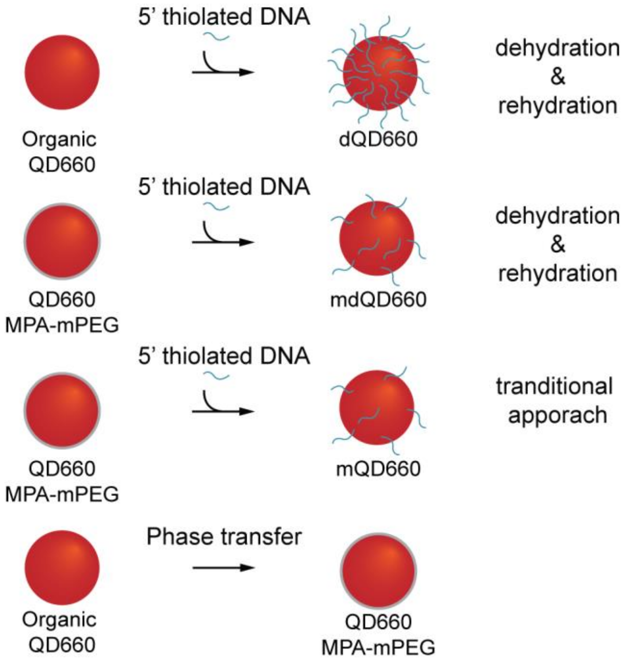


**Fig. S10.**

**Schematic for various DNA-conjugated QDs.** Schematic for preparation of various DNA-conjugated QDs (From bottom to top: QD660 MPA-mPEG, mQD660, mdQD660, and dQD660).

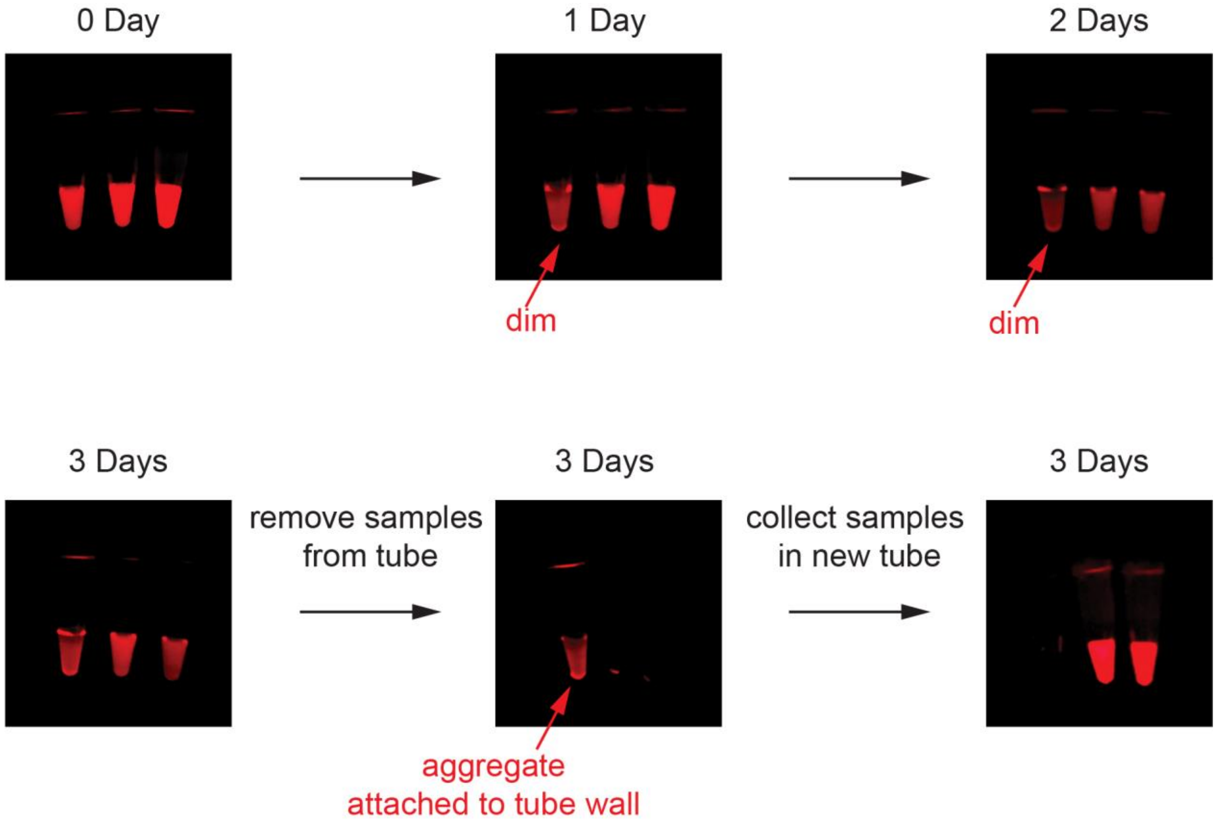


**Fig. S11.**

**Colloidal stability of diluted QDs in high salt concentration.** Three days colloidal stability of 5 nM of mQD660, mQD660 with extra free thiolated DNA in solution, and dQD660 (from left to right) in 0.5× TBE buffer with 500 mM NaCl. mQDs become dim immediately and aggregate on the tube after three days. Fluorescence images taken by digital camera (iPhone X) under blue light excitation.

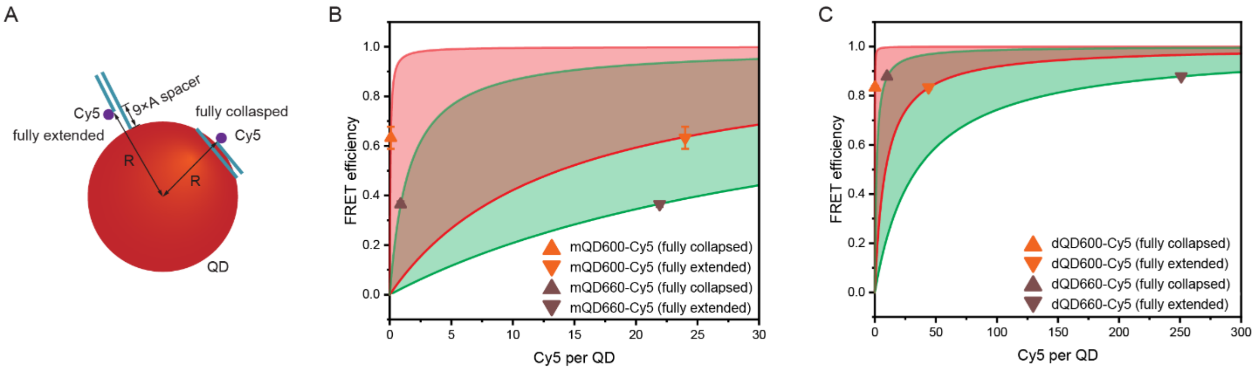


**Fig. S12.**

**Influence of DNA conformation on FRET calculation. (A)** Possible conformations of DNA on the QD and corresponding donor-acceptor distances. When DNA is conjugated to a surface with high density (dQDs/dQRs), it may tend to fully extend and orient perpendicularly to the particle surface based on entropic forces and electrostatic repulsion (*81*). In this case, the Cy5 distance is estimated to be the full length of the extended single-stranded DNA spacer (assuming contour length corresponding to 0.56 nm per base). In the other extreme, when the loading density is low (mQDs/mQRs), depending on the molecule-surface affinity, the macromolecule might adopt a variety of collapsed conformations (attached to the surface) (*82*). **(B)** FRET efficiencies calculated theoretically for different acceptors assuming the fully collapsed DNA conformation model (solid curve: $R$=3.2 nm $R_0$=5.3 nm (light red) and $R$=7.1 nm $R_0$=6.6 nm (light green) and fully extended DNA conformation model (solid curve: $R$=8.2 nm $R_0$=5.3 nm (red) and $R$=12.1 nm $R_0$=6.6 nm (green)), and using mQD emission intensities (data points). **(C)** FRET efficiencies for different acceptors calculated theoretically assuming the fully collapsed DNA conformation model (solid curve: $R$=3.2 nm $R_0$=5.7 nm (light red) and $R$=7.1 nm $R_0$=6.7 nm (light green), the fully extended DNA conformation model (solid curve: $R$=8.2 nm $R_0$=5.7 nm (red) and $R$=12.1 nm $R_0$=6.7 nm (green)), and from the dQD emission intensities (data points).

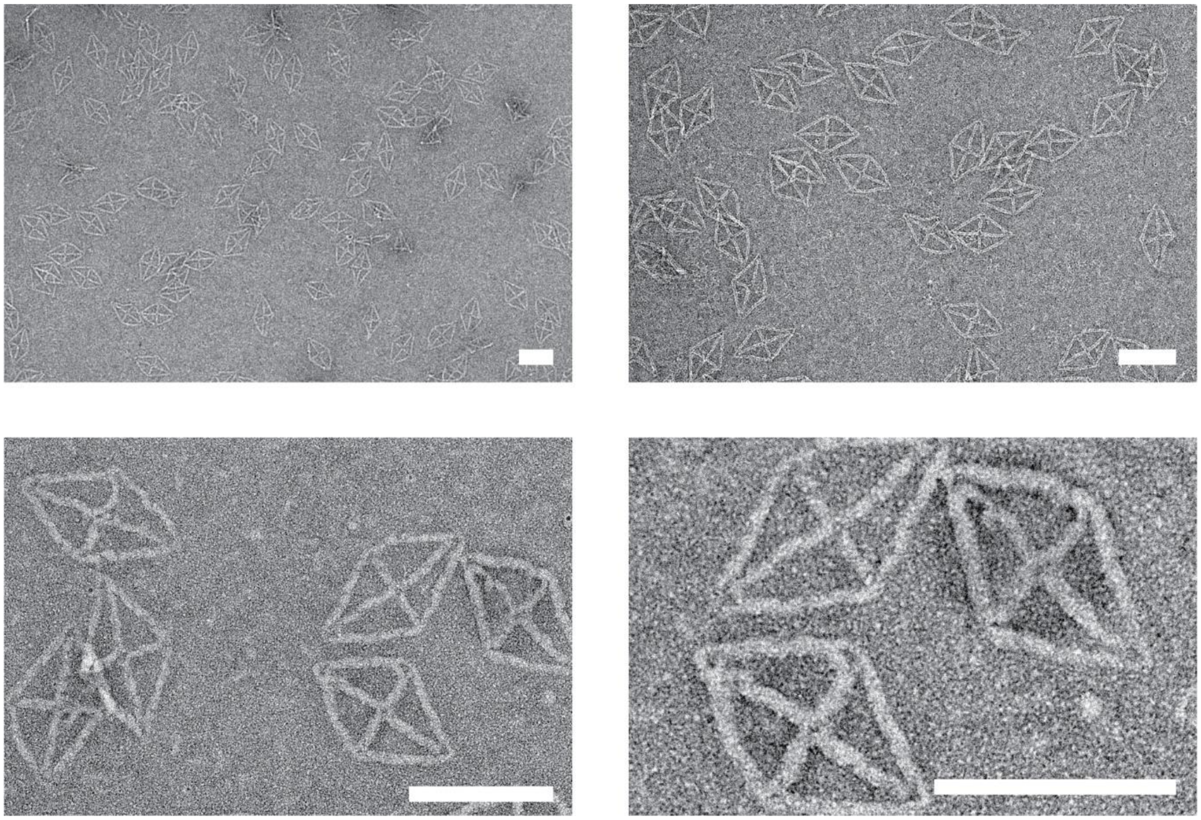

**Fig. S13.**

**TEM images of 2D rhombic DNA origami.** TEM images of 2D rhombic DNA origami at several magnifications. (Scale bars: 100 nm)

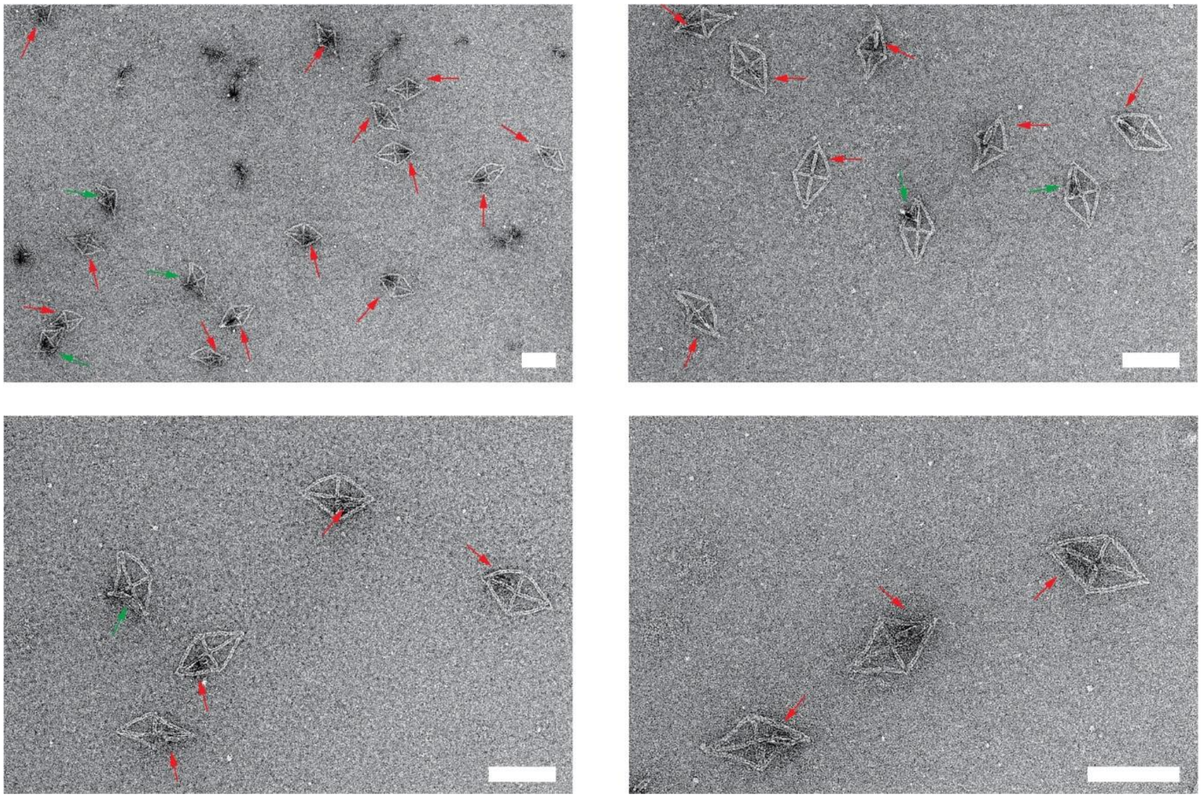

**Fig. S14.**

**TEM images of Rh-dQR620 assemblies.** TEM images of Rh-dQR620 assemblies at several magnifications. (Red arrow: aligned assemblies, green arrow: misaligned assemblies. Scale bars: 100 nm)

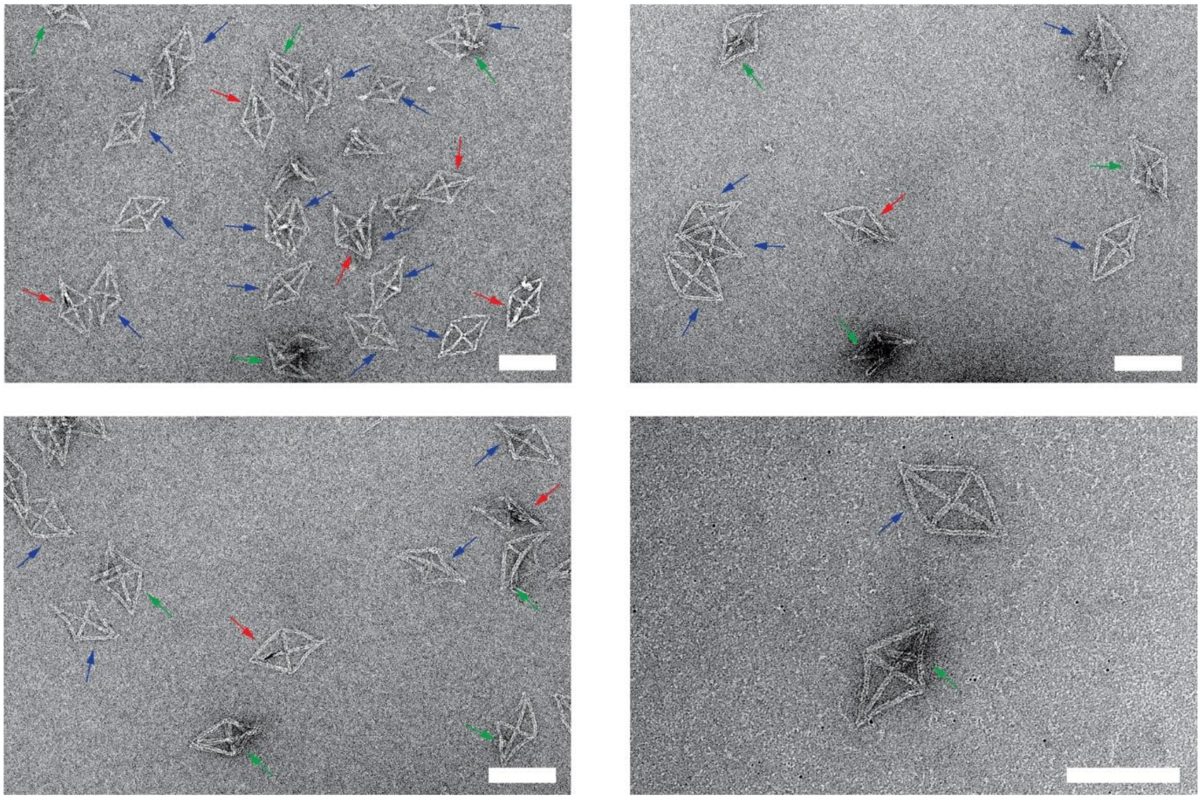

**Fig. S15.**

**TEM images of Rh-mQR620 assemblies.** TEM images of Rh-mQR620 assemblies at several magnifications. (Red arrow: monomer assemblies, green arrow: dimer assemblies, blue arrow: bare origami. Scale bars: 100 nm)

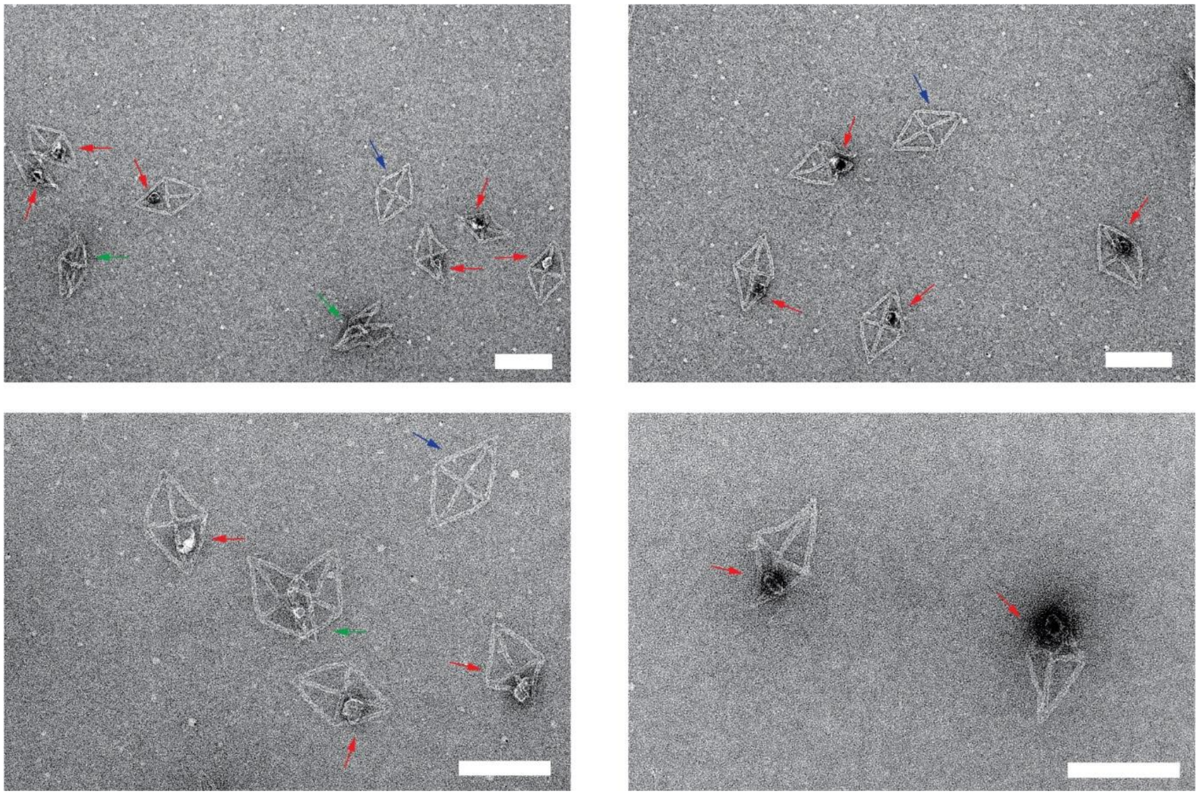

**Fig. S16.**

**TEM images of Rh-dQD660 assemblies.** TEM images of Rh-dQD660 assemblies at several magnifications. (Red arrow: monomer assemblies, green arrow: dimer assemblies, blue arrow: bare origami. Scale bars: 100 nm)

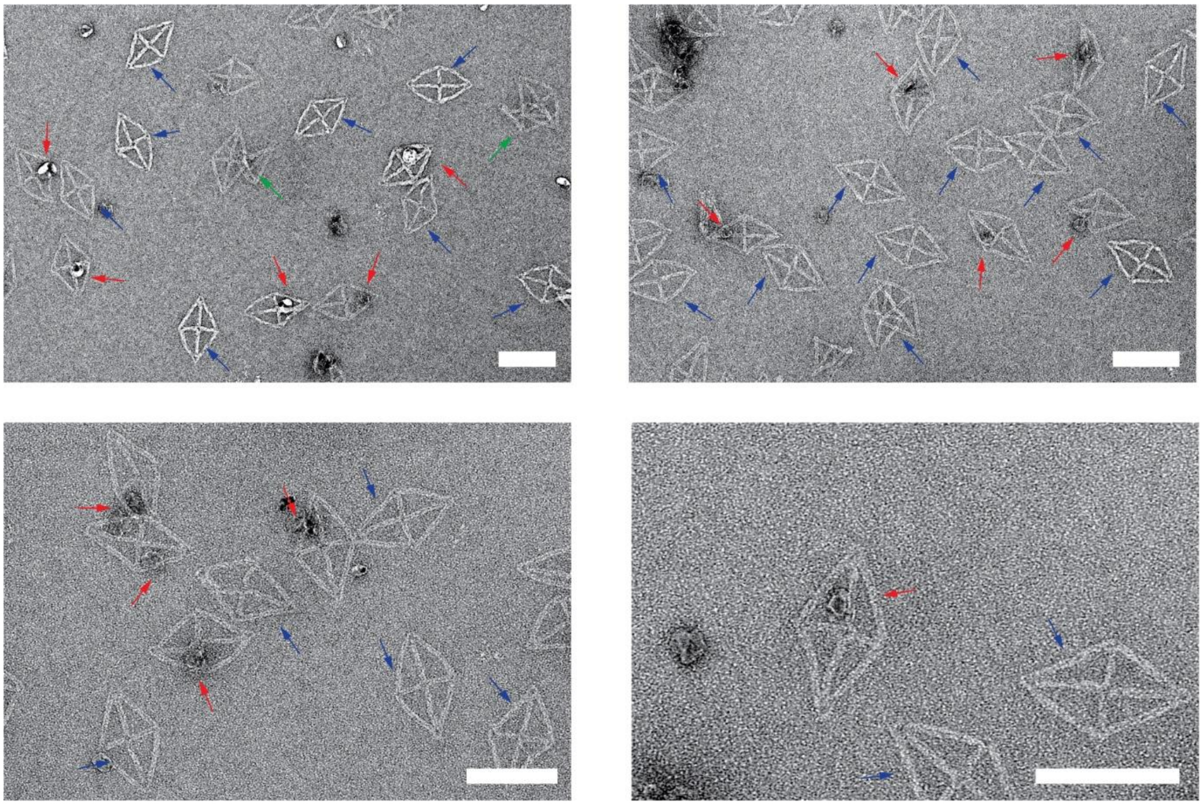

**Fig. S17.**

**TEM images of Rh-mQD660 assemblies.** TEM images of Rh-mQD660 assemblies at several magnifications. (Red arrow: monomer assemblies, green arrow: dimer assemblies, blue arrow: bare origami. Scale bars: 100 nm)

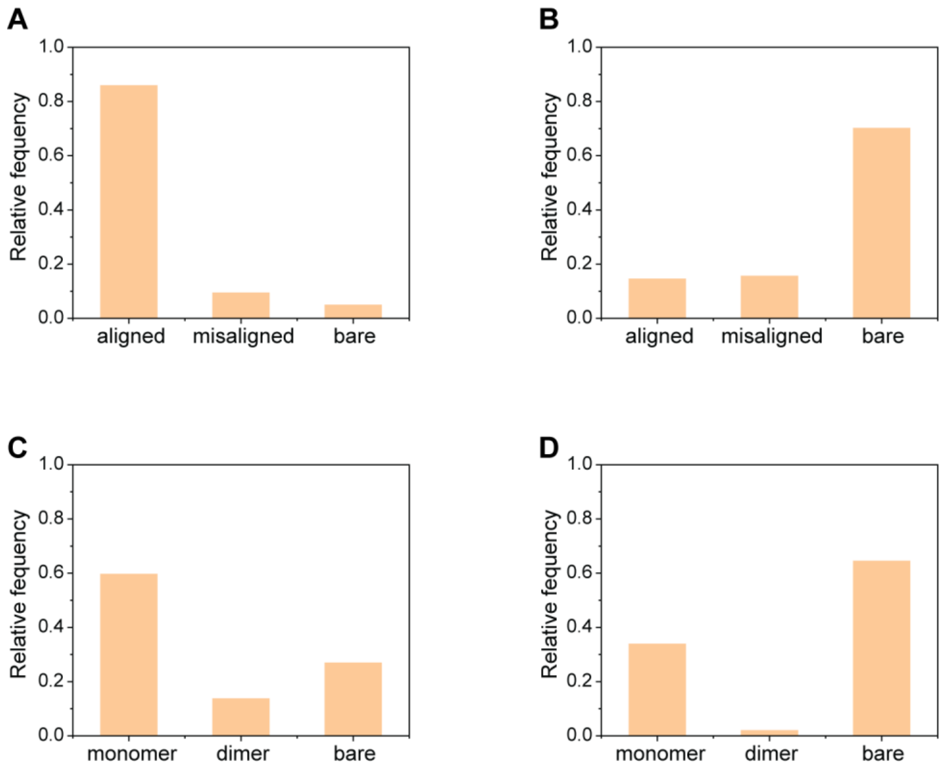


**Fig. S18.**

**The yield of Rh-QR and Rh-QD assemblies.** The yield of Rh-QR and Rh-QD assemblies using (**A**) dQR620, (**B**) mQR620, (**C**) dQD660, and (**D**) mQD660. Yields were calculated from TEM images (267 assemblies for (A), 374 assemblies for (B), 272 assemblies for (C), and 376 assemblies for (D)).

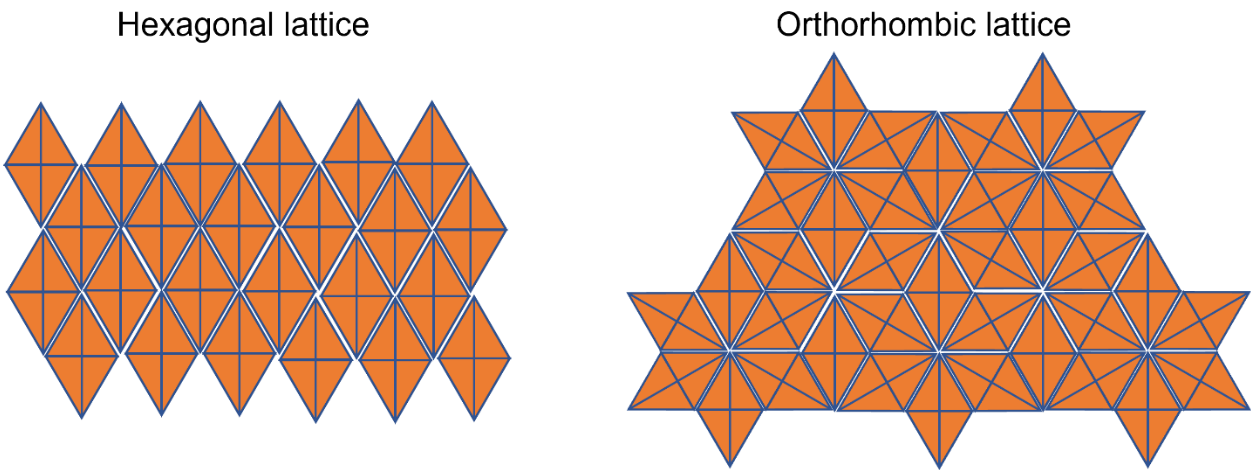


**Fig. S19.**
**The two-tiling lattice of a rhombic shape.** Left: hexagonal lattice; right: orthorhombic lattice.

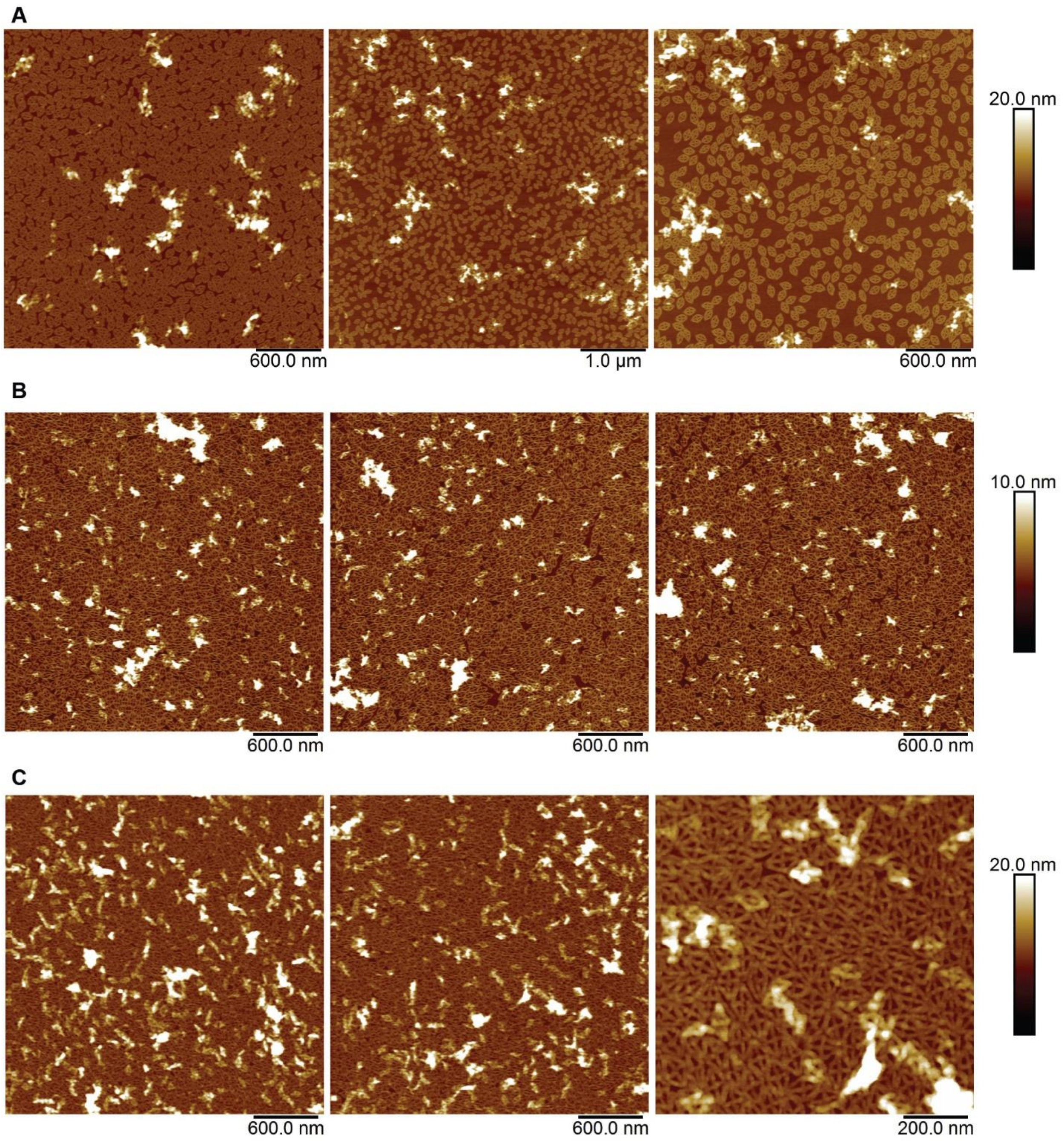


**Fig. S20.**

**SALSA with no crossover or 5 nt overhangs.** (**A**) AFM images of SALSA with rhombic origami bearing no crossover overhangs, with face-selecting overhangs. No origami lattice was formed. (**B-C**) AFM images of SALSA with rhombic origami bearing 5 nt crossover overhangs. 1 nM origami, 50 mM $Na^+$, 40-35-30°C-RT anneal (B); 1 nM origami, 80 mM $Na^+$, 45-40-35°C-3 cycles-RT anneal (C), without face-selecting overhangs. Only small grains of lattice structures were observed.

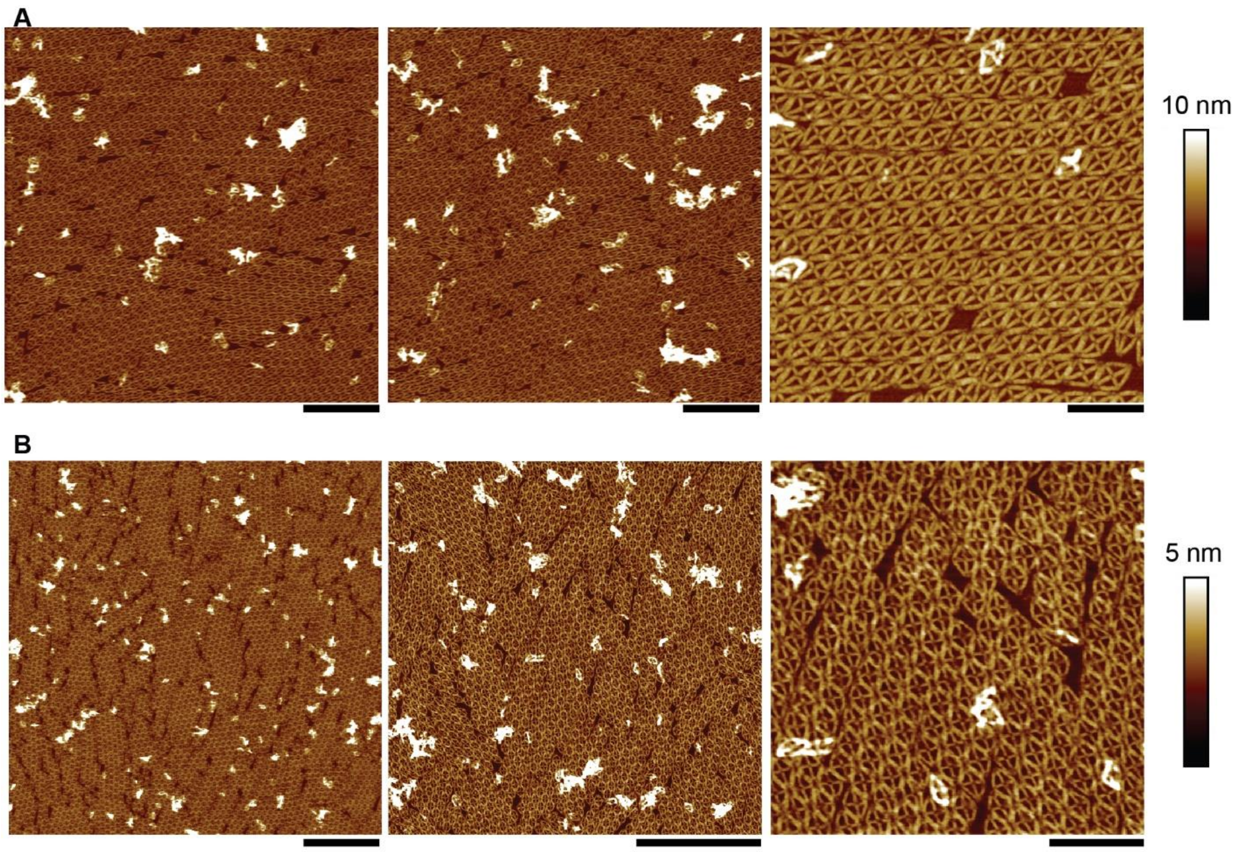


**Fig. S21.**

**Additional AFM images of SALSA with 8 nt overhangs.** 500 pM origami, 500 mM $Na^+$, 60-55-50°C-12 cycles- RT anneal (36 hr), with face-selecting overhangs. (**A**) and (**B**) are two independent experiments. Scale bars: 600 nm for (A), 1 µm for (B) (left and middle), and 250 nm for (B) (right).

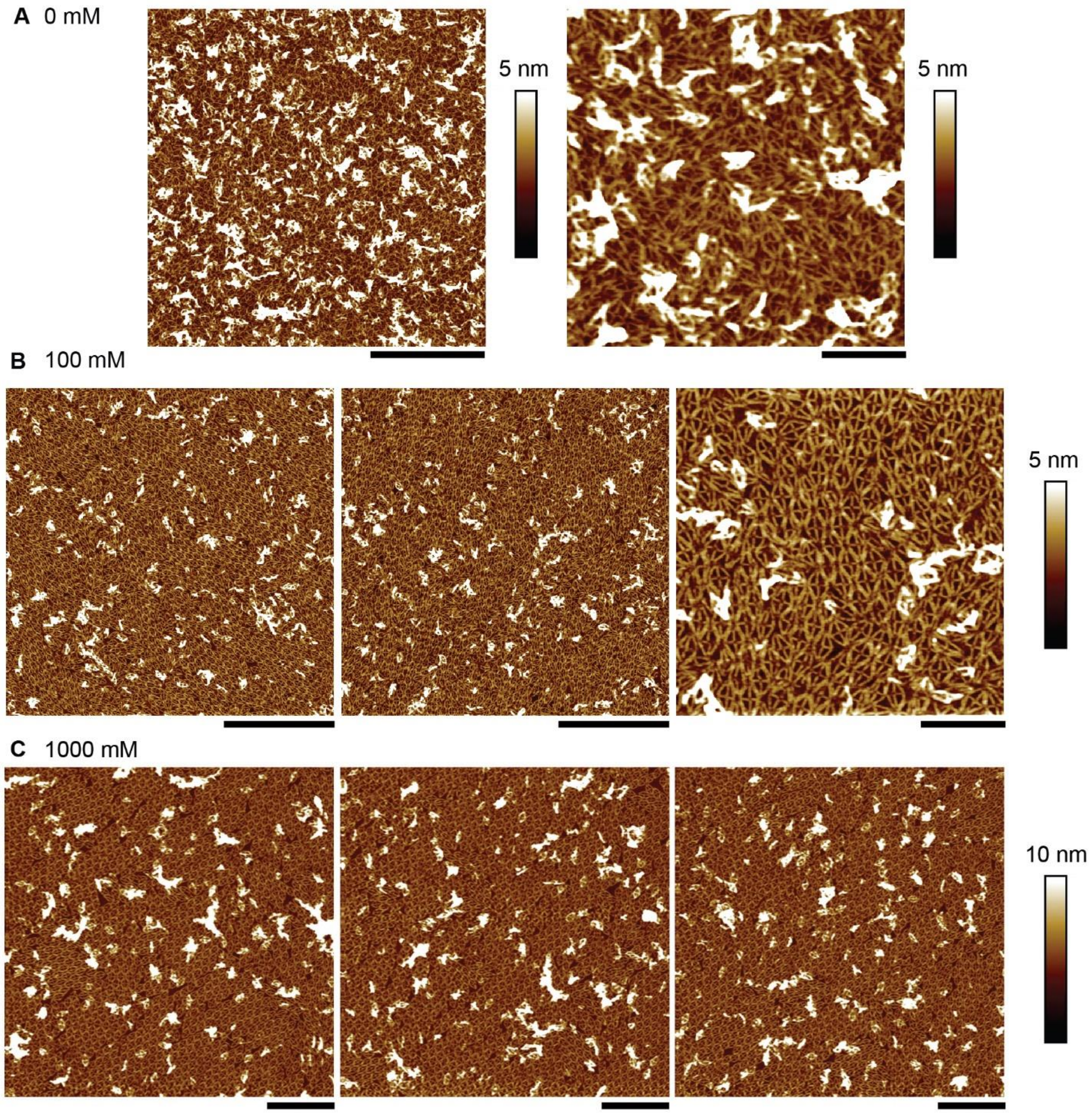


**Fig. S22.**

**SALSA with different $Na^+$ concentration.** (**A**) AFM images of SALSA with no $Na^+$. (**B**) AFM images of SALSA with 100 mM $Na^+$. (**C**) AFM images of SALSA with 1000mM $Na^+$. All experiments with 200 pM origami, 60-55-50°C-12 cycles-RT anneal (36 hr), with face-selecting overhangs. Scale bars: 1 µm for (A) (left) and (B) (left and middle), 250 nm for (A) (right) and (B) (right), and 600 nm for (C).

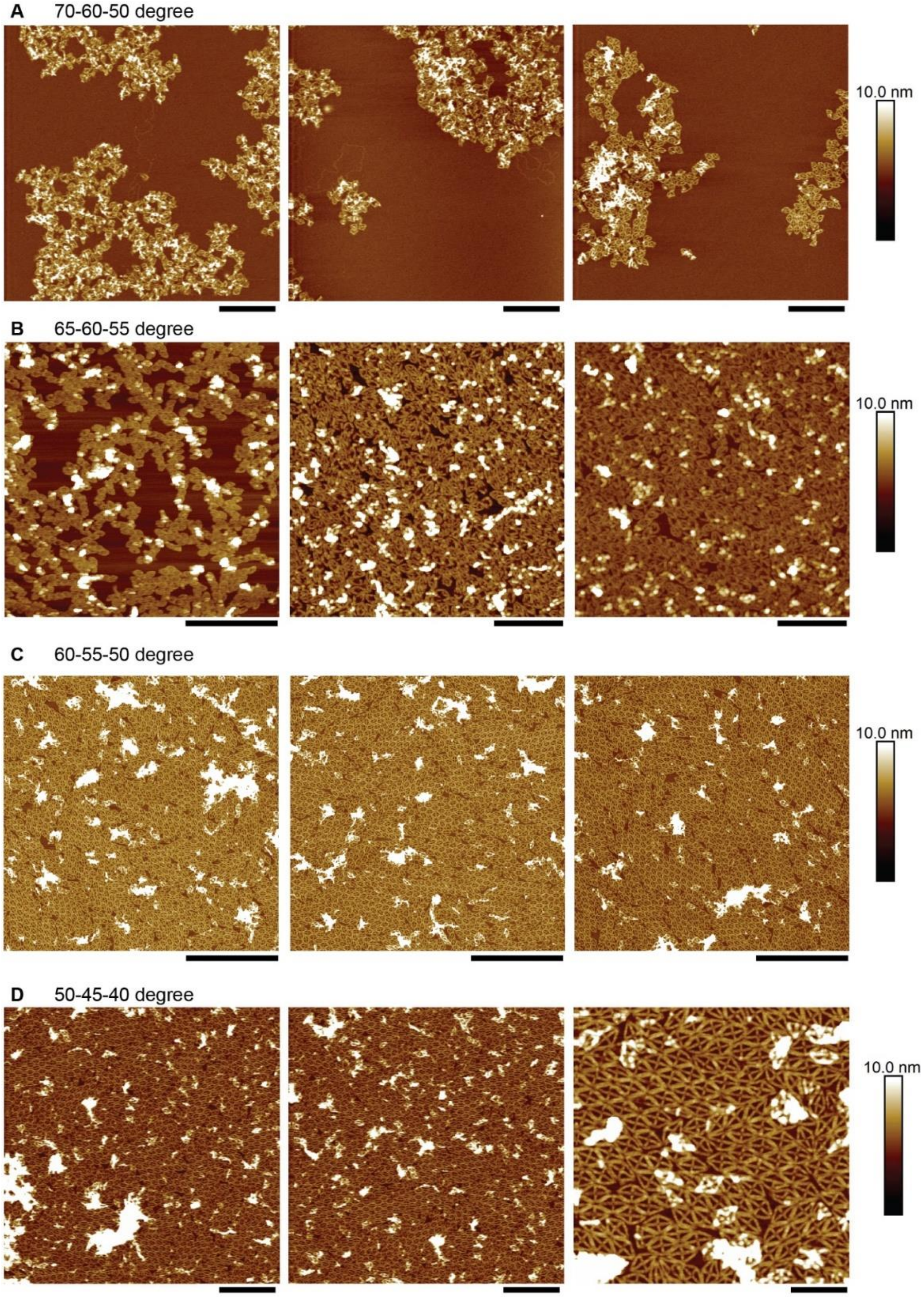


**Fig. S23.**

**SALSA with different annealing temperature.** (**A**) 70-60-50°C-5 cycles-RT anneal (15 hrs). (**B**) 65-60-55°C-5 cycles-RT anneal (15 hrs). (**C**) 60-55-50°C-5 cycles-RT anneal (15 hrs) **(D)** 50-45-40°C-5 cycles-RT anneal (15 hrs). All without face-selecting overhangs. Scale bars: 600 nm for (A) and (D) (left and middle), 1 µm for (B) (left) and (C) (left), 500 nm for (B) (middle and right), and 200 nm for (D) (right).

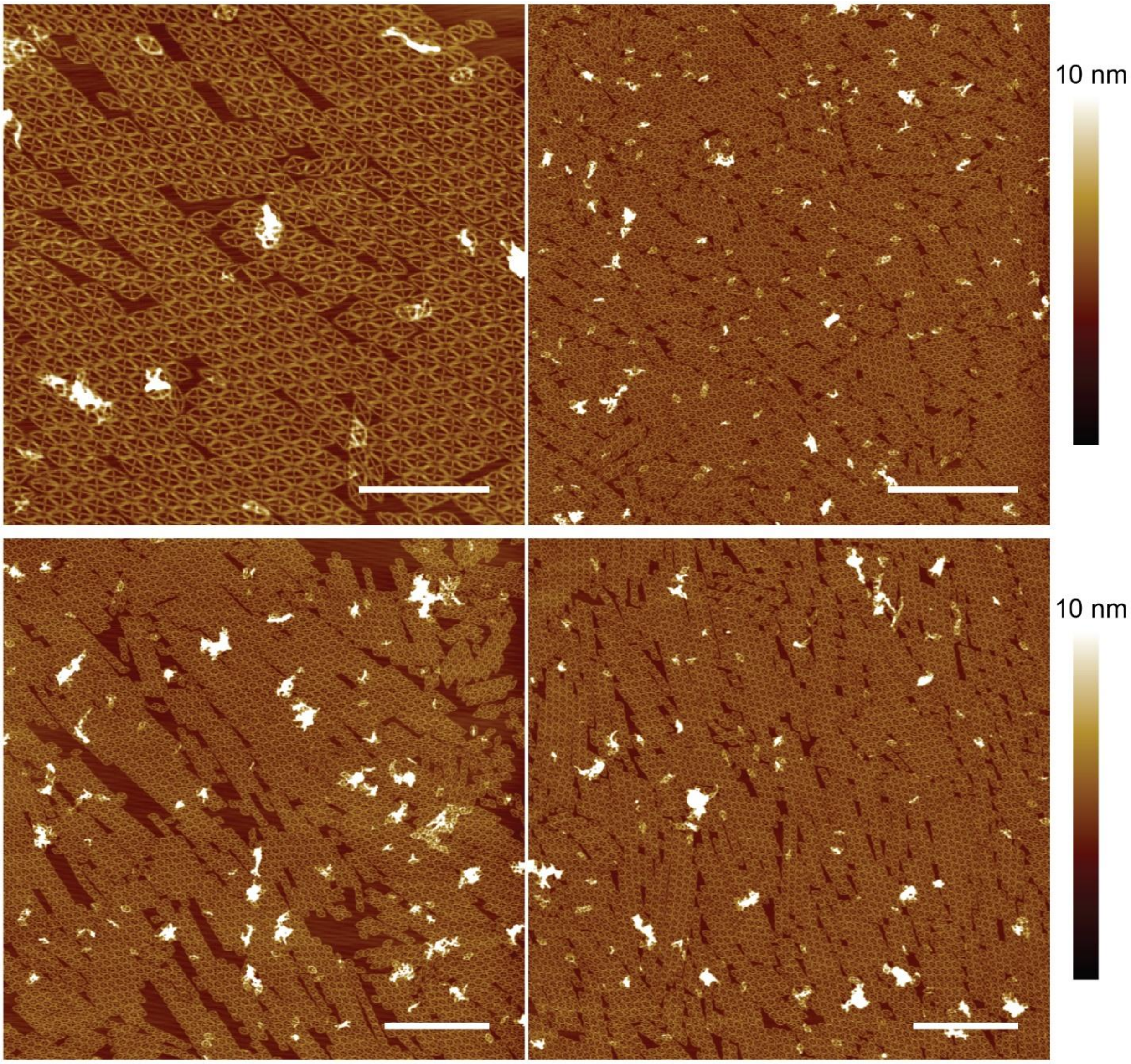


**Fig. S24.**

**AFM images of SALSA with 12 cycles of annealing.** 500 pM origami, 500 mM $Na^+$, 60-55-50°C-40 cycles-RT anneal (120 hr), with face-selecting overhangs. Scale bars: 500 nm for the top and 1 µm for the bottom.

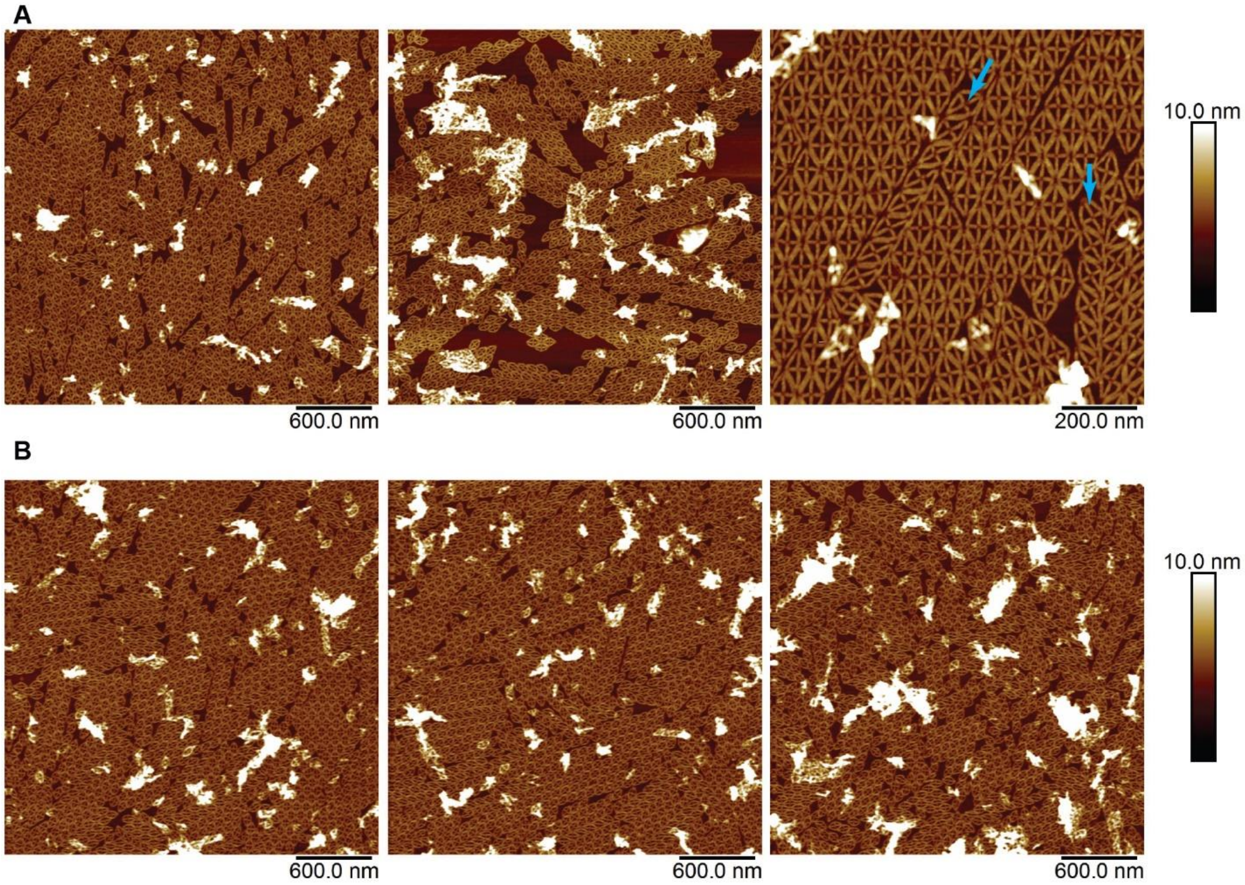


**Fig. S25.**

**Additional AFM images of SALSA with no face-selecting overhangs.** 200 pM origami, 60-55-50°C-12 cycles- RT anneal (36 hr). (**A**) With 300 mM $Na^+$. (**B**) With 500 mM $Na^+$. Arrows indicate lattices with the opposite side landing on the substrate.

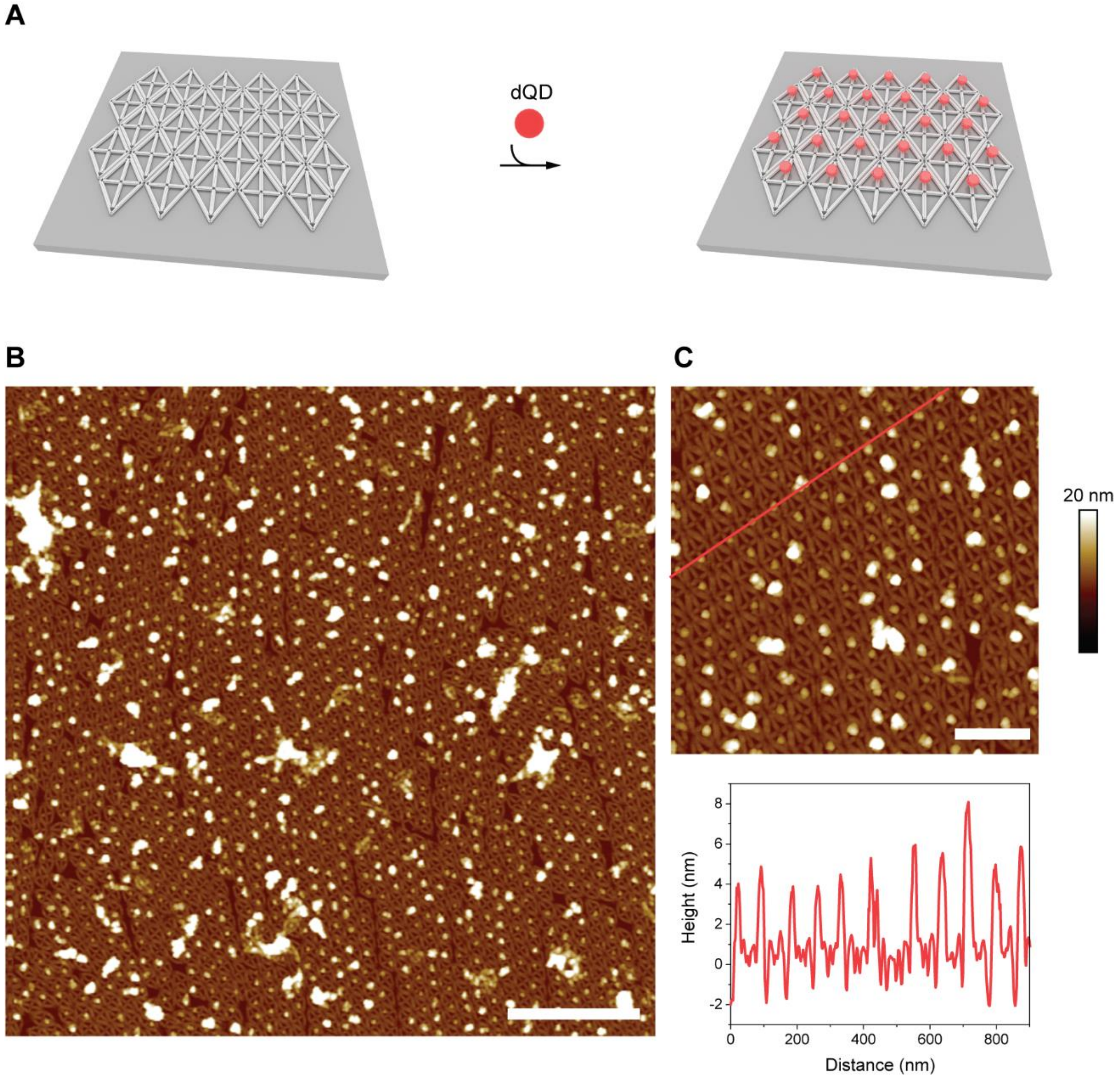


**Fig. S26.**

**Scalable 2D QD array with nanoscale precision using dQD and SALSA with face-selecting DNA origami.** (**A**) Schematic for fabrication of 2D QD600 array with nanoscale precision using dQD600 and SALSA with face-selecting DNA origami. (**B**) An overview AFM image of 2D QD600 array showing the scalable ability. (**C**) A selected zoomed area from (B) showing the nanoscale precision (top) and the height profile of the red line showing the inter-dot spacing control (bottom). Scale bars: 600 nm and 200 nm for (B) and (C), respectively.

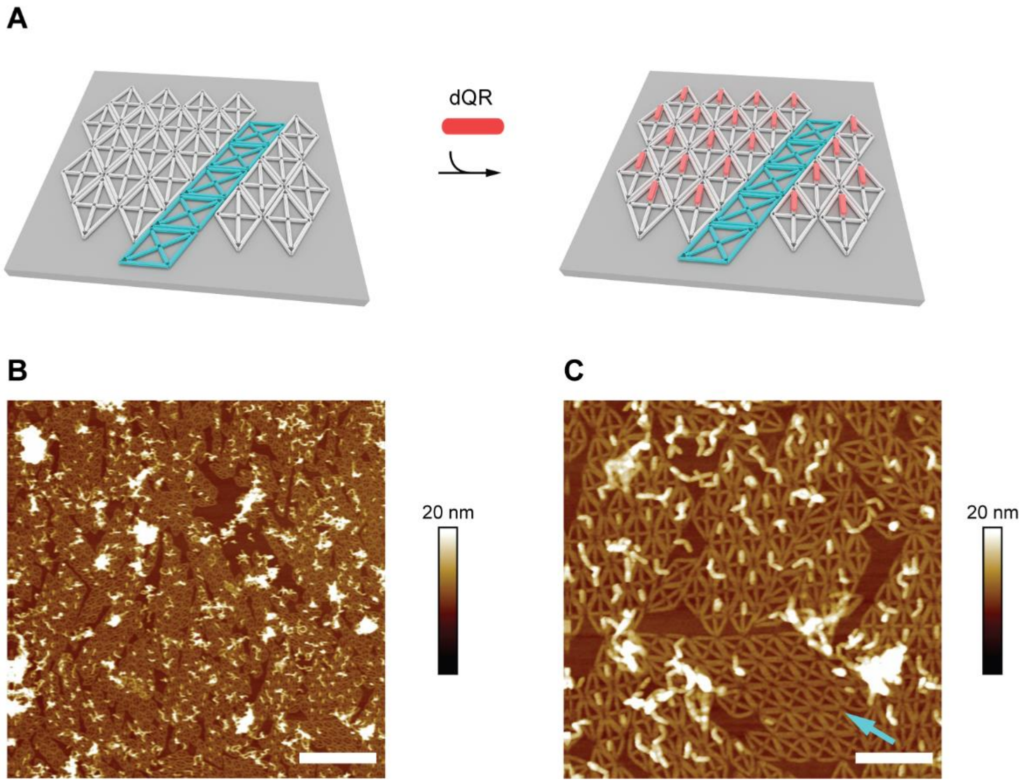


**Fig. S27.**

**Scalable 2D QR array with nanoscale precision using dQR and SALSA without face-selecting DNA origami.** (**A**) Schematic for fabrication of 2D QR620 array using dQR620 and SALSA without face-selecting DNA origami. The DNA origami can land on the mica substrate facing up (white) or down (cyan). Each species assembles into separate lattices. (**B**) An overview AFM image of 2D QR620 array showing the poor scalable ability, and (**C**) a selected zoomed area from (B) showing the poor nanoscale precision and low QR loading efficiency due to DNA origami with the random side facing up. The cyan arrow indicates the DNA origami with binding face down. Scale bars: 600 nm and 200 nm for (B) and (C), respectively.

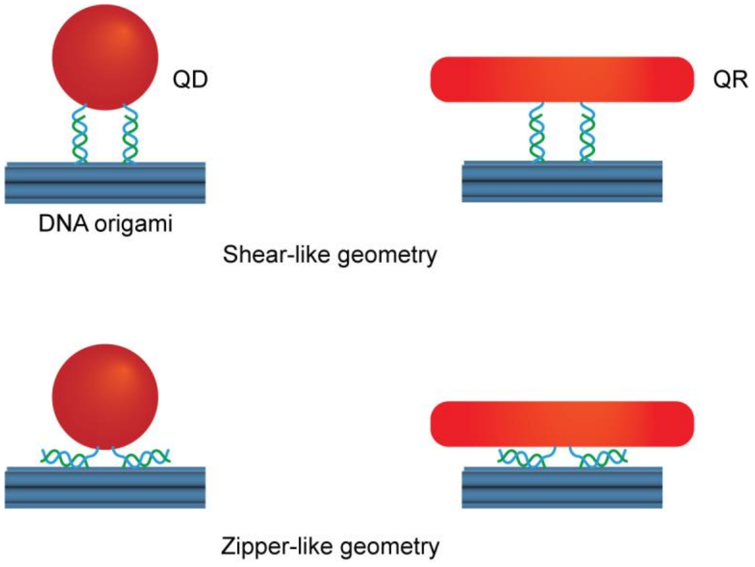


**Fig. S28.**

**Scheme for shear-like geometry and for QD/QR origami assemblies.** In the shear-like geometry, the QD/QR is conjugated to the 5’ end of DNA strand and hybridized with the 5’ end of the complementary strand from the overhang of DNA origami. In the zipper-like geometry, the QD/QR is conjugated to the 3’ end of DNA strand and hybridized with the 5’ end of the complementary strand from the overhang of DNA origami. Zipper and shear here refer to the different forces necessary to pull the DNAs apart (*69*).

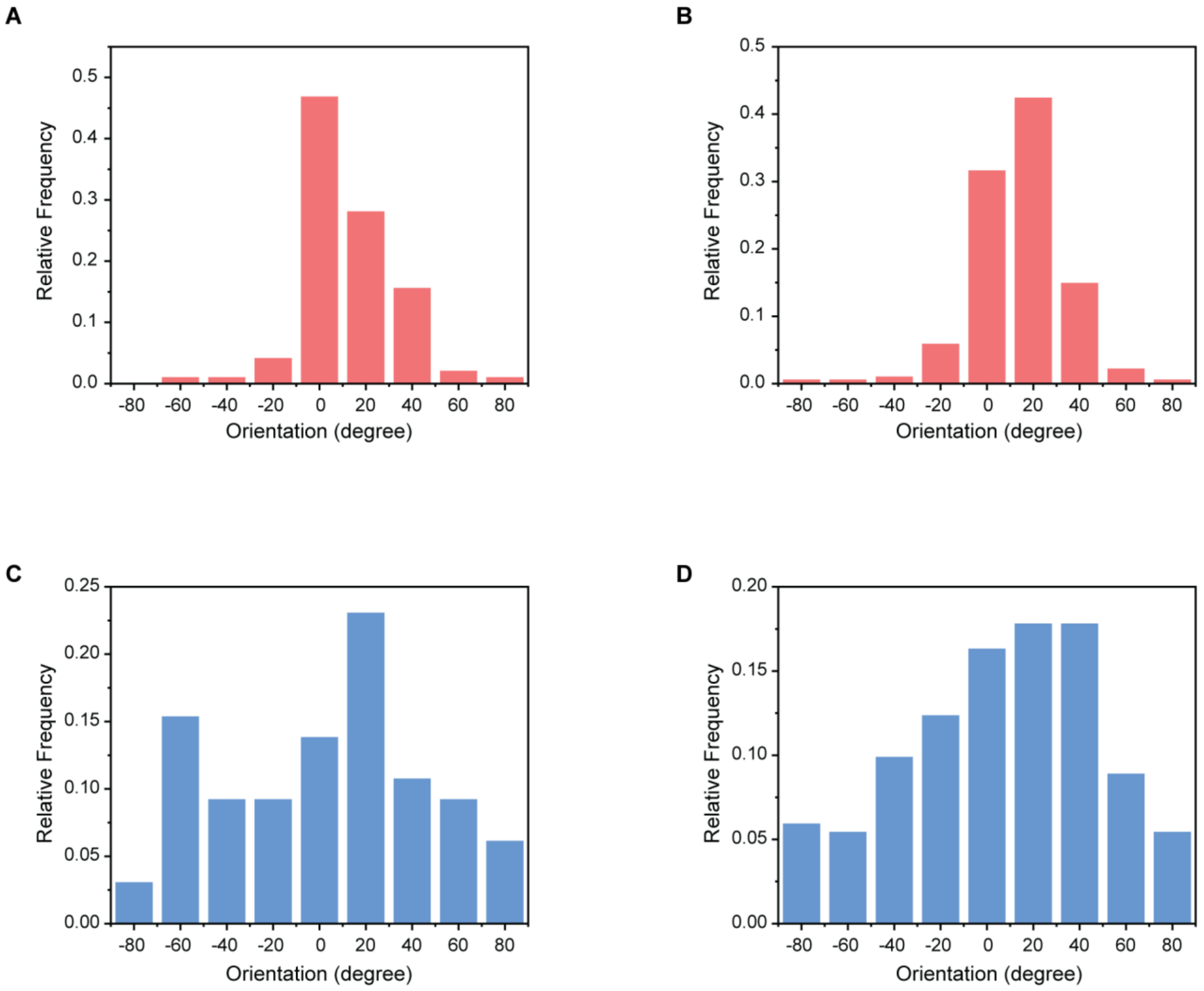


**Fig. S29.**

**Corresponding flat histogram for orientation analysis of Fig. 6B–6E.** QR orientation distributions of 2D arrays using Rh with crossovers in a 1 μm$^2$ **(A)** or 9 μm$^2$ (**B**) area. QR orientation distributions of 2D arrays using Rh without crossovers in a 1 μm$^2$ **(C)** or 9 μm$^2$ (**D**) area.

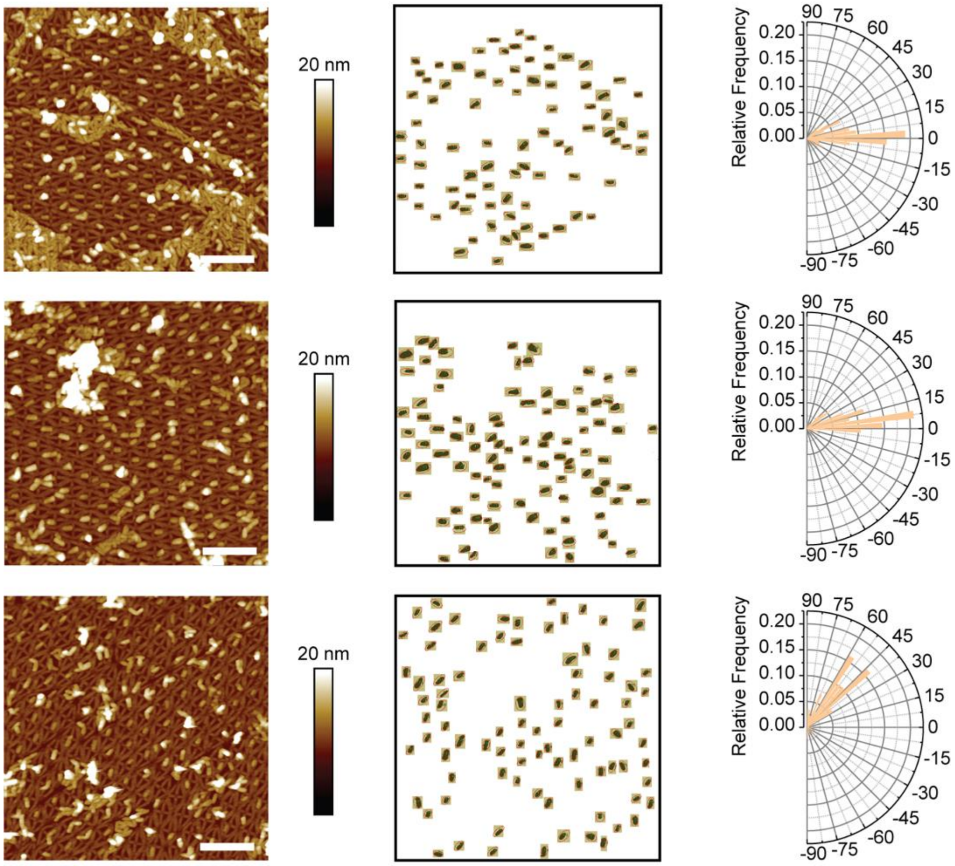


**Fig. S30.**

**Orientation analysis of 2D QR620 arrays in independent experiments using zipper-like DNA hybridization, templated by origami lattices formed with crossover-bearing origamis.** Left: AFM images of 2D QR620 array from different experiments (Scale bars: 200 nm). Middle: images of isolated QR620 monomers for orientation analysis using *Fiji* (see Methods). Right: the orientation distribution calculated using *OrientationJ* (see Methods) (from top to bottom: 84, 96, and 87 QRs).

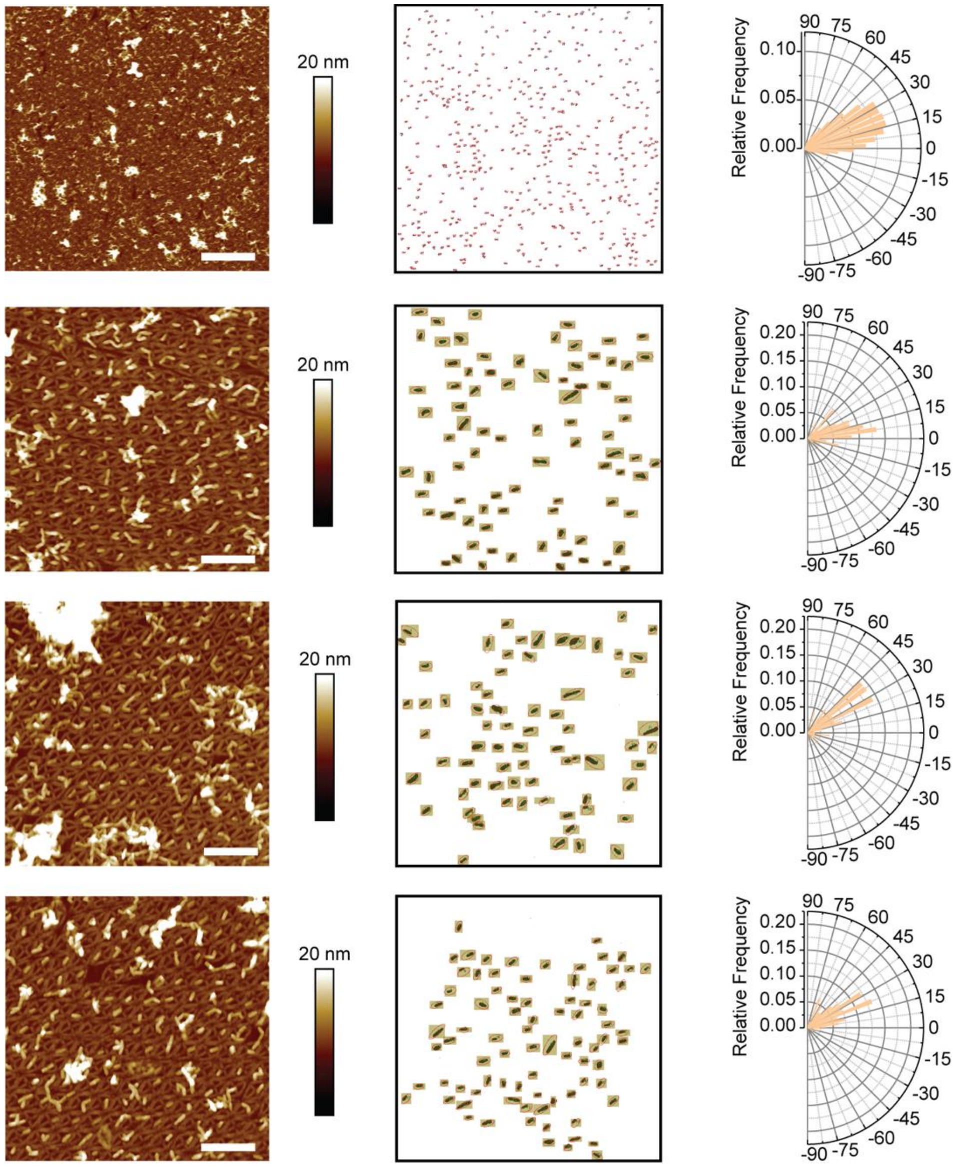


**Fig. S31.**

**Orientation analysis of 2D QR620 arrays in independent experiments using shear-like DNA hybridization, templated by origami lattices formed with crossover-bearing origamis.** Left: AFM images of 2D QR620 array from different experiments (Scale bars: 600 nm for the first image, 200 nm for the rest images). Middle: images of isolated QR620 monomers for orientation analysis using *Fiji* (see Methods). Right: the orientation distribution calculated using *OrientationJ* (see Methods) (from top to bottom: 492, 82, 71, and 82 QRs).

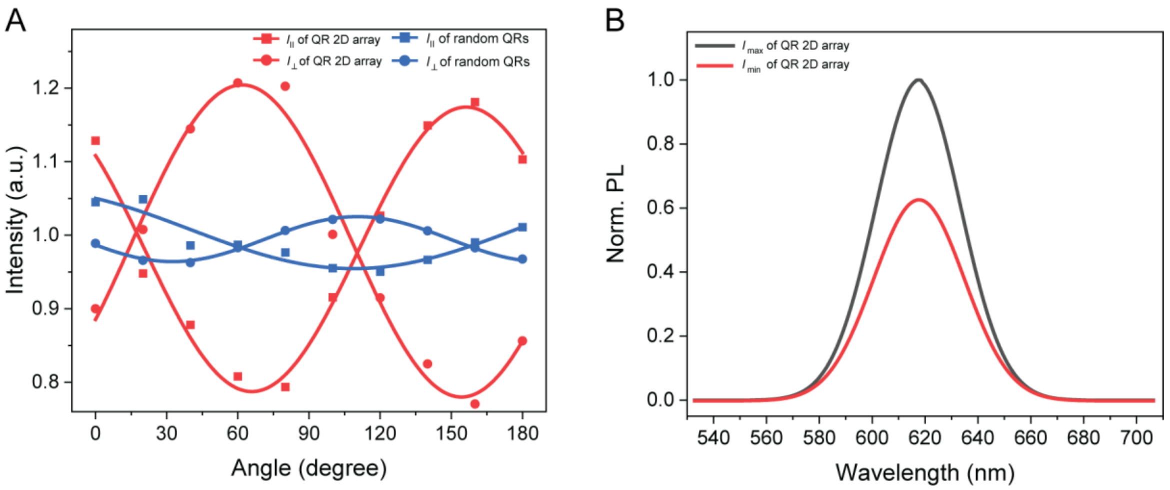


**Fig. S32.**

**Polarization measurement of QR 2D array and random QRs.** (**A**) Plot of $I_{II}$ and $I_{\perp}$ (the curve is obtained by fitting the experimental data to a sinusoidal function) as a function of the polarizer angle. (**B**) Representative normalized spectra of $I_{max}$ and $I_{min}$ for QR 2D array.

**Table S1.**

**Comparison of various thiolated DNA conjugation on QDs and QRs.**

| Reference | Method | Diameter (nm) | DNA number | Time |
|---|---|---|---|---|
| *JACS* 1999 (*32*) | Salt-aging with aqueous MPA capped QDs | N/A. | N/A | 24-40 h |
| *JPCB 2005* (*33*) | Salt-aging with aqueous MPA capped QDs | 3.8 ± 0.8 | ~6 | Overnight |
| *Chem. Commun.* 2005 (*34*) | Salt-aging with aqueous MPA capped QDs | N/A | 2 | 24-40 h |
| *ACS Appl. Mater. Interfaces* 2022 (*36*) | One-step ligand-exchange/phase-transfer from commonly available hydrophobic QDs | 7.5 ± 1 | 9.6 ± 0.7 | Several hours |
| This work | Sonication and dehydration from hydrophobic QDs and QRs | 6.3 ± 0.5<br>14.2 ± 2.3<br>4.0 ± 0.5/16.2 ± 2.4<br>5.0 ± 0.6/29.1 ± 2.9 | ~21<br>~135<br>~42<br>~105 | Several minutes |

**Table S2.**
**DNA density of dQDs/dQRs and mQDs/mQRs.**

| | Surface area ($nm^2$) | DNA per $nm^2$ | pmol/$cm^2$ |
|---|---|---|---|
| **dQD600** | 125 | 0.165 | 27.48 |
| **dQD660** | 633 | 0.213 | 35.34 |
| **dQR560** | 229 | 0.184 | 30.63 |
| **dQR620** | 496 | 0.212 | 35.15 |
| **mQD600** | 125 | 0.024 | 3.96 |
| **mQD660** | 633 | 0.015 | 2.41 |
| **mQR560** | 229 | 0.025 | 4.16 |
| **mQR620** | 496 | 0.023 | 3.87 |

**Table S3.**

**Quantum yield of DNA functionalized QDs/QRs using different conjugation methods** (*P*-values are from Student's t-test).

| | **Sample 1** | **Sample 2** | **Sample 3** | **Mean** | **SD** | ***P*-value** |
|---|---|---|---|---|---|---|
| **mQD600** | 0.129 | 0.144 | 0.117 | 0.130 | 0.014 | 0.0042 |
| **dQD600** | 0.188 | 0.220 | 0.198 | 0.202 | 0.016 | |
| **mQD660** | 0.178 | 0.142 | 0.157 | 0.159 | 0.018 | 0.1404 |
| **dQD660** | 0.189 | 0.168 | 0.217 | 0.191 | 0.025 | |
| **mQR560** | 0.119 | 0.083 | 0.072 | 0.091 | 0.025 | 0.0065 |
| **dQR560** | 0.177 | 0.233 | 0.236 | 0.215 | 0.033 | |
| **mQR560** | 0.305 | 0.253 | 0.277 | 0.278 | 0.026 | 0.0003 |
| **mQR620** | 0.602 | 0.560 | 0.532 | 0.565 | 0.035 | |

**Table S4.**

**DNA density per dQD/dQR using NaCl or NaOH.** *P*-values are from Student's t-test.

| | **Sample 1** | **Sample 2** | **Sample 3** | **Mean** | **SD** | ***P*-value** |
|---|---|---|---|---|---|---|
| **dQD600 (NaCl)** | 18.8 | 19.2 | 20.1 | 19.4 | 0.7 | 0.1964 |
| **dQD600 (NaOH)** | 19.4 | 20.6 | 21.9 | 20.6 | 1.2 | |
| **dQD660 (NaCl)** | 134.6 | 120.3 | 130.8 | 128.6 | 7.4 | 0.4729 |
| **dQD660 (NaOH)** | 134.7 | 123.9 | 144.5 | 134.4 | 10.3 | |
| **mQR560 (NaCl)** | 43.5 | 50.7 | 51.2 | 48.5 | 4.3 | 0.1845 |
| **dQR560 (NaOH)** | 38.4 | 39.9 | 48.2 | 42.2 | 5.3 | |
| **mQR560 (NaCl)** | 87.9 | 95.2 | 105.4 | 96.2 | 8.8 | 0.2627 |
| **mQR620 (NaOH)** | 96.4 | 107.1 | 111.4 | 105.0 | 7.7 | |

**Table S5.**

**Photophysical properties of the FRET compounds within the DNA hybridization experiment*.**

| **Fluorophore** | **$\varepsilon$ ($M^{-1}$ $cm^{-1}$) [$\lambda$]** | **$\Phi$** |
|---|---|---|
| QD600 | $3.14 \times 10^6$ [350 nm] | 0.20 ± 0.02 (dQD)<br>0.13 ± 0.02 (mQD) |
| QD660 | $2.90 \times 10^7$ [350 nm] | 0.19 ± 0.03 (dQD)<br>0.16 ± 0.02 (mQD) |
| QR560 | $2.28 \times 10^7$ [350 nm] | 0.22 ± 0.04 (dQR)<br>0.09 ± 0.03 (mQR) |
| QR620 | $6.45 \times 10^7$ [350 nm] | 0.56 ± 0.04 (dQR)<br>0.28 ± 0.03 (mQR) |
| Cy5 | $2.50 \times 10^5$ [648 nm] | 0.27 |
| **FRET pair (D → A)** | **$J$ ($M^{-1}$. $cm^{-1}$. $nm^4$)** | **$R_0$ (nm)** |
| dQD600 → Cy5 | $1.0 \times 10^{16}$ | 5.7 ± 0.2 |
| mQD600 → Cy5 | $1.0 \times 10^{16}$ | 5.3 ± 0.2 |
| dQD660 → Cy5 | $2.8 \times 10^{16}$ | 6.7 ± 0.2 |
| mQD660 → Cy5 | $2.8 \times 10^{16}$ | 6.6 ± 0.2 |
| dQR560 → Cy5 | $2.8 \times 10^{15}$ | 4.7 ± 0.2 |
| mQR560 → Cy5 | $2.8 \times 10^{15}$ | 4.1 ± 0.2 |
| dQR620 → Cy5 | $1.9 \times 10^{16}$ | 7.5 ± 0.1 |
| mQR620 → Cy5 | $2.0 \times 10^{16}$ | 6.7 ± 0.1 |

* $\varepsilon$ and $\Phi$ values of Cy5 were provided by the commercial vendors. $\varepsilon$ and $\Phi$ values of QD were calculated using empirical formula (*74–76*) and relative quantum yield determination method (*79*), respectively. $J$ was calculated using **Eq. (1)**. $R_0$ was calculated using **Eq. (2)**.

**Table S6.**

**Scaffold sequences for wireframe DNA origami.**

| Scaffold | Length (nt) | Sequence (5'-3') |
|---|---|---|
| **M13** | 7249 | AATGCTACTACTATTAGTAGAATTGATGCCACCTTTTCAGCTCGCGCCCCAAATGAAAATATAGCTAAACAGGTTATTGACCATTTGCGAAATGTATCT<br>AATGGTCAAACTAAATCTACTCGTTCGCAGAATTGGGAATCAACTGTTATATGGAATGAAACTTCCAGACACCGTACTTTAGTTGCATATTTAAAACAT<br>GTTGAGCTACAGCATTATATTCAGCAATTAAGCTCTAAGCCATCCGCAAAAATGACCTCTTATCAAAAGGAGCAATTAAAGGTACTCTCTAATCCTGA<br>CCTGTTGGAGTTTGCTTCCGGTCTGGTTCGCTTTGAAGCTCGAATTAAAACGCGATATTTGAAGTCTTTCGGGCTTCCTCTTAATCTTTTTGATGCAATC<br>CGCTTTGCTTCTGACTATAATAGTCAGGGTAAAGACCTGATTTTTGATTTATGGTCATTCTCGTTTTCTGAACTGTTTAAAGCATTTGAGGGGGATTCAA<br>TGAATATTTATGACGATTCCGCAGTATTGGACGCTATCCAGTCTAAACATTTTACTATTACCCCCTCTGGCAAAACTTCTTTTGCAAAAGCCTCTCGCTA<br>TTTTGGTTTTTATCGTCGTCTGGTAAACGAGGGTTATGATAGTGTTGCTCTTACTATGCCTCGTAATTCCTTTTGGCGTTATGTATCTGCATTAGTTGAAT<br>GTGGTATTCCTAAATCTCAACTGATGAATCTTTCTACCTGTAATAATGTTGTTCCGTTAGTTCGTTTTATTAACGTAGATTTTTCTTCCCAACGTCCTGAC<br>TGGTATAATGAGCCAGTTCTTAAAATCGCATAAGGTAATTCACAATGATTAAAGTTGAAATTAAACCATCTCAAGCCCAATTTACTACTCGTTCTGGTG<br>TTTCTCGTCAGGGCAAGCCTTATTCACTGAATGAGCAGCTTTGTTACGTTGATTTGGGTAATGAATATCCGGTTCTTGTCAAGATTACTCTTGATGAAGG<br>TCAGCCAGCCTATGCGCCTGGTCTGTACACCGTTCATCTGTCCTCTTTCAAAGTTGGTCAGTTCGGTTCCCTTATGATTGACCGTCTGCGCCTCGTTCCG<br>GCTAAGTAACATGGAGCAGGTCGCGGATTTCGACACAATTTATCAGGCGATGATACAAATCTCCGTTGTACTTTGTTTCGCGCTTGGTATAATCGCTGG<br>GGGTCAAAGATGAGTGTTTTAGTGTATTCTTTTGCCTCTTTCGTTTTAGGTTGGTGCCTTCGTAGTGGCATTACGTATTTTACCCGTTTAATGGAAACTTC<br>CTCATGAAAAAGTCTTTAGTCCTCAAAGCCTCTGTAGCCGTTGCTACCCTCGTTCCGATGCTGTCTTTCGCTGCTGAGGGTGACGATCCCGCAAAAGCG<br>GCCTTTAACTCCCTGCAAGCCTCAGCGACCGAATATATCGGTTATGCGTGGGCGATGGTTGTTGTCATTGTCGGCGCAACTATCGGTATCAAGCTGTTT<br>AAGAAATTCACCTCGAAAGCAAGCTGATAAACCGATACAATTAAAGGCTCCTTTTGGAGCCTTTTTTTTGGAGATTTTCAACGTGAAAAAATTATTATT<br>CGCAATTCCTTTAGTTGTTCCTTTCTATTCTCACTCCGCTGAAACTGTTGAAAGTTGTTTAGCAAAATCCCATACAGAAAATTCATTTACTAACGTCTGG<br>AAAGACGACAAAACTTTAGATCGTTACGCTAACTATGAGGGCTGTCTGTGGAATGCTACAGGCGTTGTAGTTTGTACTGGTGACGAAACTCAGTGTTA<br>CGGTACATGGGTTCCTATTGGGCTTGCTATCCCTGAAAATGAGGGTGGTGGCTCTGAGGGTGGCGGTTCTGAGGGTGGCGGTTCTGAGGGTGGCGGTA<br>CTAAACCTCCTGAGTACGGTGATACACCTATTCCGGGCTATACTTATATCAACCCTCTCGACGGCACTTATCCGCCTGGTACTGAGCAAAACCCCGCTA<br>ATCCTAATCCTTCTCTTGAGGAGTCTCAGCCTCTTAATACTTTCATGTTTCAGAATAATAGGTTCCGAAATAGGCAGGGGGCATTAACTGTTTATACGG<br>GCACTGTTACTCAAGGCACTGACCCCGTTAAAACTTATTACCAGTACACTCCTGTATCATCAAAAGCCATGTATGACGCTTACTGGAACGGTAAATTCA<br>GAGACTGCGCTTTCCATTCTGGCTTTAATGAGGATTTATTTGTTTGTGAATATCAAGGCCAATCGTCTGACCTGCCTCAACCTCCTGTCAATGCTGGCGG<br>CGGCTCTGGTGGTGGTTCTGGTGGCGGCTCTGAGGGTGGTGGCTCTGAGGGTGGCGGTTCTGAGGGTGGCGGCTCTGAGGGAGGCGGTTCCGGTGGTG<br>GCTCTGGTTCCGGTGATTTTGATTATGAAAAGATGGCAAACGCTAATAAGGGGGCTATGACCGAAAATGCCGATGAAAACGCGCTACAGTCTGACGCT<br>AAAGGCAAACTTGATTCTGTCGCTACTGATTACGGTGCTGCTATCGATGGTTTCATTGGTGACGTTTCCGGCCTTGCTAATGGTAATGGTGCTACTGGT<br>GATTTTGCTGGCTCTAATTCCCAAATGGCTCAAGTCGGTGACGGTGATAATTCACCTTTAATGAATAATTTCCGTCAATATTTACCTTCCCTCCCTCAAT<br>CGGTTGAATGTCGCCCTTTTGTCTTTGGCGCTGGTAAACCATATGAATTTTCTATTGATTGTGACAAAATAAACTTATTCCGTGGTGTCTTTGCGTTTCTT<br>TTATATGTTGCCACCTTTATGTATGTATTTTCTACGTTTGCTAACATACTGCGTAATAAGGAGTCTTAATCATGCCAGTTCTTTTGGGTATTCCGTTATTA<br>TTGCGTTTCCTCGGTTTCCTTCTGGTAACTTTGTTCGGCTATCTGCTTACTTTTCTTAAAAAGGGCTTCGGTAAGATAGCTATTGCTATTTCATTGTTTCT<br>TGCTCTTATTATTGGGCTTAACTCAATTCTTGTGGGTTATCTCTCTGATATTAGCGCTCAATTACCCTCTGACTTTGTTCAGGGTGTTCAGTTAATTCTCC<br>CGTCTAATGCGCTTCCCTGTTTTTATGTTATTCTCTCTGTAAAGGCTGCTATTTTCATTTTTGACGTTAAACAAAAAATCGTTTCTTATTTGGATTGGGAT<br>AAATAATATGGCTGTTTATTTTGTAACTGGCAAATTAGGCTCTGGAAAGACGCTCGTTAGCGTTGGTAAGATTCAGGATAAAATTGTAGCTGGGTGCA<br>AAATAGCAACTAATCTTGATTTAAGGCTTCAAAACCTCCCGCAAGTCGGGAGGTTCGCTAAAACGCCTCGCGTTCTTAGAATACCGGATAAGCCTTCT<br>ATATCTGATTTGCTTGCTATTGGGCGCGGTAATGATTCCTACGATGAAAATAAAAACGGCTTGCTTGTTCTCGATGAGTGCGGTACTTGGTTTAATACC<br>CGTTCTTGGAATGATAAGGAAAGACAGCCGATTATTGATTGGTTTCTACATGCTCGTAAATTAGGATGGGATATTATTTTTCTTGTTCAGGACTTATCTA<br>TTGTTGATAAACAGGCGCGTTCTGCATTAGCTGAACATGTTGTTTATTGTCGTCGTCTGGACAGAATTACTTTACCTTTTGTCGGTACTTTATATTCTCTT<br>ATTACTGGCTCGAAAATGCCTCTGCCTAAATTACATGTTGGCGTTGTTAAATATGGCGATTCTCAATTAAGCCCTACTGTTGAGCGTTGGCTTTATACTG<br>GTAAGAATTTGTATAACGCATATGATACTAAACAGGCTTTTTCTAGTAATTATGATTCCGGTGTTTATTCTTATTTAACGCCTTATTTATCACACGGTCG<br>GTATTTCAAACCATTAAATTTAGGTCAGAAGATGAAATTAACTAAAATATATTTGAAAAAGTTTTCTCGCGTTCTTTGTCTTGCGATTGGATTTGCATCA<br>GCATTTACATATAGTTATATAACCCAACCTAAGCCGGAGGTTAAAAAGGTAGTCTCTCAGACCTATGATTTTGATAAATTCACTATTGACTCTTCTCAG<br>CGTCTTAATCTAAGCTATCGCTATGTTTTCAAGGATTCTAAGGGAAAATTAATTAATAGCGACGATTTACAGAAGCAAGGTTATTCACTCACATATATT<br>GATTTATGTACTGTTTCCATTAAAAAAGGTAATTCAAATGAAATTGTTAAATGTAATTAATTTTGTTTTCTTGATGTTTGTTTCATCATCTTCTTTTGCTC<br>AGGTAATTGAAATGAATAATTCGCCTCTGCGCGATTTTGTAACTTGGTATTCAAAGCAATCAGGCGAATCCGTTATTGTTTCTCCCGATGTAAAAGGTA<br>CTGTTACTGTATATTCATCTGACGTTAAACCTGAAAATCTACGCAATTTCTTTATTTCTGTTTTACGTGCAAATAATTTTGATATGGTAGGTTCTAACCCT<br>TCCATTATTCAGAAGTATAATCCAAACAATCAGGATTATATTGATGAATTGCCATCATCTGATAATCAGGAATATGATGATAATTCCGCTCCTTCTGGT<br>GGTTTCTTTGTTCCGCAAAATGATAATGTTACTCAAACTTTTAAAATTAATAACGTTCGGGCAAAGGATTTAATACGAGTTGTCGAATTGTTTGTAAAG<br>TCTAATACTTCTAAATCCTCAAATGTATTATCTATTGACGGCTCTAATCTATTAGTTGTTAGTGCTCCTAAAGATATTTTAGATAACCTTCCTCAATTCCT<br>TTCAACTGTTGATTTGCCAACTGACCAGATATTGATTGAGGGTTTGATATTTGAGGTTCAGCAAGGTGATGCTTTAGATTTTTCATTTGCTGCTGGCTCT<br>CAGCGTGGCACTGTTGCAGGCGGTGTTAATACTGACCGCCTCACCTCTGTTTTATCTTCTGCTGGTGGTTCGTTCGGTATTTTTAATGGCGATGTTTTAG<br>GGCTATCAGTTCGCGCATTAAAGACTAATAGCCATTCAAAAATATTGTCTGTGCCACGTATTCTTACGCTTTCAGGTCAGAAGGGTTCTATCTCTGTTG<br>GCCAGAATGTCCCTTTTATTACTGGTCGTGTGACTGGTGAATCTGCCAATGTAAATAATCCATTTCAGACGATTGAGCGTCAAAATGTAGGTATTTCCA<br>TGAGCGTTTTTCCTGTTGCAATGGCTGGCGGTAATATTGTTCTGGATATTACCAGCAAGGCCGATAGTTTGAGTTCTTCTACTCAGGCAAGTGATGTTAT<br>TACTAATCAAAGAAGTATTGCTACAACGGTTAATTTGCGTGATGGACAGACTCTTTTACTCGGTGGCCTCACTGATTATAAAAACACTTCTCAGGATTC<br>TGGCGTACCGTTCCTGTCTAAAATCCCTTTAATCGGCCTCCTGTTTAGCTCCCGCTCTGATTCTAACGAGGAAAGCACGTTATACGTGCTCGTCAAAGC<br>AACCATAGTACGCGCCCTGTAGCGGCGCATTAAGCGCGGCGGGTGTGGTGGTTACGCGCAGCGTGACCGCTACACTTGCCAGCGCCCTAGCGCCCGCT<br>CCTTTCGCTTTCTTCCCTTCCTTTCTCGCCACGTTCGCCGGCTTTCCCCGTCAAGCTCTAAATCGGGGGCTCCCTTTAGGGTTCCGATTTAGTGCTTTACG<br>GCACCTCGACCCCAAAAAACTTGATTTGGGTGATGGTTCACGTAGTGGGCCATCGCCCTGATAGACGGTTTTTCGCCCTTTGACGTTGGAGTCCACGTT<br>CTTTAATAGTGGACTCTTGTTCCAAACTGGAACAACACTCAACCCTATCTCGGGCTATTCTTTTGATTTATAAGGGATTTTGCCGATTTCGGAACCACCA<br>TCAAACAGGATTTTCGCCTGCTGGGGCAAACCAGCGTGGACCGCTTGCTGCAACTCTCTCAGGGCCAGGCGGTGAAGGGCAATCAGCTGTTGCCCGTC<br>TCACTGGTGAAAAGAAAAACCACCCTGGCGCCCAATACGCAAACCGCCTCTCCCCGCGCGTTGGCCGATTCATTAATGCAGCTGGCACGACAGGTTTC<br>CCGACTGGAAAGCGGGCAGTGAGCGCAACGCAATTAATGTGAGTTAGCTCACTCATTAGGCACCCCAGGCTTTACACTTTATGCTTCCGGCTCGTATGT<br>TGTGTGGAATTGTGAGCGGATAACAATTTCACACAGGAAACAGCTATGACCATGATTACGAATTCGAGCTCGGTACCCGGGGATCCTCTAGAGTCGAC<br>CTGCAGGCATGCAAGCTTGGCACTGGCCGTCGTTTTACAACGTCGTGACTGGGAAAACCCTGGCGTTACCCAACTTAATCGCCTTGCAGCACATCCCCC<br>TTTCGCCAGCTGGCGTAATAGCGAAGAGGCCCGCACCGATCGCCCTTCCCAACAGTTGCGCAGCCTGAATGGCGAATGGCGCTTTGCCTGGTTTCCGG<br>CACCAGAAGCGGTGCCGGAAAGCTGGCTGGAGTGCGATCTTCCTGAGGCCGATACTGTCGTCGTCCCCTCAAACTGGCAGATGCACGGTTACGATGCG<br>CCCATCTACACCAACGTGACCTATCCCATTACGGTCAATCCGCCGTTTGTTCCCACGGAGAATCCGACGGGTTGTTACTCGCTCACATTTAATGTTGAT<br>GAAAGCTGGCTACAGGAAGGCCAGACGCGAATTATTTTTGATGGCGTTCCTATTGGTTAAAAAATGAGCTGATTTAACAAAAATTTAATGCGAATTTT<br>AACAAAATATTAACGTTTACAATTTAAATATTTGCTTATACAATCTTCCTGTTTTTGGGGCTTTTCTGATTATCAACCGGGGTACATATGATTGACATGC<br>TAGTTTTACGATTACCGTTCATCGATTCTCTTGTTTGCTCCAGACTCTCAGGCAATGACCTGATAGCCTTTGTAGATCTCTCAAAAATAGCTACCCTCTC<br>CGGCATTAATTTATCAGCTAGAACGGTTGAATATCATATTGATGGTGATTTGACTGTCTCCGGCCTTTCTCACCCTTTTGAATCTTTACCTACACATTAC<br>TCAGGCATTGCATTTAAAATATATGAGGGTTCTAAAAATTTTTATCCTTGCGTTGAAATAAAGGCTTCTCCCGCAAAAGTATTACAGGGTCATAATGTT<br>TTTGGTACAACCGATTTAGCTTTATGCTCTGAGGCTTTATTGCTTAATTTTGCTAATTCTTTGCCTTGCCTGTATGATTTATTGGATGTT |

## Table S7.

**Staple sequences for the 6HB wireframe rhombic DNA origami and the same origami with overhangs.** Blue: crossover overhangs; Red: QD/QR binding overhangs.

| Staple No. | Length (nt) | Sequences (5'--3') |
|---|---|---|
| **1** | 38 | CCTCATTAAGAAACGCTTTTTTTAAGACACCAGTTACC |
| **2** | 48 | CATATAAAAAGCCAAAAGCGTAAGAATAAGAAGAAGCGGATAAGTGCC |
| **3** | 42 | ACCCTTCCTTGCTGCGGGGTTTTGCTCACGGAATAGTGGCAA |
| **4** | 42 | TATCGGCTGACCTGGAATGGAAAGCGCACATAAAGCCCAAAA |
| **5** | 42 | AGGATTACATGATTGTAGAAAATACATAGTCTCTGAGATAGA |
| **6** | 42 | CGTTCCAAGCAAACAAGACTCCTTATTAGACTCCTCCGCCAG |
| **7** | 50 | GTGCCCCAGTATGTTGTAAGCGAAGGGACATTCTGGAATATTACAAGAGA |
| **8** | 34 | CCATTGCGTAATAATCATACATGGCTTTGTAACA |
| **9** | 42 | CTGAAACCCTGCCTGGTCAGTGCCTTGATGATGATCAGTCAC |
| **10** | 42 | TAACGGATTTCGGAAAGGCAAAAGAATCATCGATAGAAGTTT |
| **11** | 55 | ATGGATGCACCGTAATCAGTATGAAACACACTAAAACACTCACGAAGGTGGTTTT |
| **12** | 50 | TGCCACTATCTTTGCGGAAACGTCACCAAGCGACAGGGCGCCAGTCTGAA |
| **13** | 42 | TCTTTTCGCGTATTGAATCAAGTTTGCCGCAAGGCACCCCCA |
| **14** | 39 | ATTATTTAGCGGGGAGAGGCGGTTTACCAGTGTACGTAA |
| **15** | 45 | AAGCAAACTCTCGGTCATAGCCCCACCAGTAGCTTTTTTATTACC |
| **16** | 46 | GCTGGCTTTGTGTCGAGCCAGCAAAATCCTTATTAAACCAGACCGG |
| **17** | 42 | CCATCTTGGAATTAGAAATCCGCGACCTGACCAGGAGTCAGG |
| **18** | 42 | CCATTTGTTCATAATTTAATTCGAGCTTTTATACCCGCATAG |
| **19** | 42 | TTCAAATGAAGAAAATGAACGGTGTACAGCTCCATACTTGAG |
| **20** | 42 | ACGTTGGATCGCGTTCAAAATCACCGGAGTCACCGGTTACTT |
| **21** | 42 | TTGAAAGACGAGGCGGTGAATTATCACCACCAGAGGAAAGAC |
| **22** | 42 | CGGAACCCATTAAAGCAGACGGTCAATCATAAGAATGACGGA |
| **23** | 42 | AATTATTGCCTCCCAAAAAGATTAAGAGAAACGAAACCAACT |
| **24** | 42 | AACAACATTGCATCTCAGAGCCGCCACCTAAATATTCAATAT |
| **25** | 42 | TTTTTTATGAATAATGAGGGAGGGAAGGCTCAGAAAGAAGCA |
| **26** | 41 | CCTCAGAAACCGATCCTTGCTTCTGTAATCATTTGGAGATT |
| **27** | 57 | ACAAATTTTTAGGGCGACATTCGCCACCATTTACCCTGACTATATCAGTTAATTACC |
| **28** | 53 | GACCATAAATTTTTTATCAGGTCCCCTCAGATTTTTCACCAGAACCACCAAAG |
| **29** | 44 | TTTTAACCCTTGAATGGTTTACCAGCGCCACCAGATTTAGCGAA |
| **30** | 42 | CCAGCATATTCATAAACATAGCGATAGCCTGAGAGTAATATC |
| **31** | 42 | ATAGAAATGACAGGTATTCTAAGAACGCAGAAAAAACTACCT |
| **32** | 42 | GAAGGCTAATTTACTCAAAATCATAGGTTTAGATTACAATCA |
| **33** | 45 | CCATCCTTATCCGGAGGTTGAGGCAGGTTTTTGTCAAGACGCTGA |
| **34** | 39 | GAAGAGTCAAAACGGAATAAGTTTACAGACGAAGATATA |
| **35** | 57 | ATTTTTTTTTTGTTAAATCAGCTCAAAATGAAGCCTTAAATCATGCAGAATGATGCA |
| **36** | 53 | CCTCCCGACTTTTTTGAGGTTTTTATTTGCATTTTTAACAGAAATAAAATTAA |

| | | |
|---|---|---|
| **37** | 31 | GATTCGAAAATTCGCGAAATTGAACGAGAAT |
| **38** | 57 | AATATTTTGCAAGAAAAACTAATGCAGATATTCAGAACGTAGATTTTCAGGATAACG |
| **39** | 42 | GAGCAAAAAGTTACCATCGGGAGAAACATTTAACGAATGCTT |
| **40** | 42 | CAGAGGCGAAGGCCAGGCAAACAGTACCTTTTAAAAATCGCG |
| **41** | 38 | TGGGAAGCGCTTCTGGTTTTTTTGCCGGAAACATCAAT |
| **42** | 48 | CCGGCACGGCGATCCGTTGTAAAACGACGAGGTGCGGCTCCAAAAGGA |
| **43** | 42 | TTTCCCATCGGAACGTTGAAAATCTCCATAAATGCCAGCTTT |
| **44** | 42 | CACTAAAGTCACGAGGTGCGGGCCTCTTCTCCAGCAATGCCT |
| **45** | 42 | AATAATTGTGTAGGTCAGGAAGATCGCACGCTATTCCAGGGT |
| **46** | 42 | CTGGCGAATCGGCCTAAAGATTCAAAAGAAGGAATAGAGCTT |
| **47** | 55 | GAGGATTAAAGCCGGAAAGGAACAACTAGGTGAGAGGGACGACGACAGTAAGGGG |
| **48** | 50 | GATGTGGAAGTATTAGTTTGAGAAGGCCGGAGACAGGAGAATAGCGAACG |
| **49** | 42 | GCGGAGTTCAAATCCCGTGCATCTGCCAGACTTTATACATTT |
| **50** | 42 | TGGCGAGTAGATAACAAACAATTCGACAATCGTAAACCATCA |
| **51** | 42 | TGGGCGCACTCGTAATTAGAGCCGTCAAAAAGGAAAGTTTCA |
| **52** | 42 | CTAACAAAGGAGCGTTTTGCTAAACAACATTCAACGTGTAGA |
| **53** | 42 | CTTTGCCCACGTTGCGTTCTAGCTGATATATGGGAGGCGCTA |
| **54** | 42 | TTTTCTGAATTAATGTAATGGGATAGGTCGAACGTAGGAGCA |
| **55** | 42 | GGGCGCTTATCTTTTATTAATTTTAAAAATTGACCGCCGGAG |
| **56** | 39 | GCGGGTTTGAGAAGGTTATCTAAAAGGCAAGTAAATGAA |
| **57** | 45 | GCCATATTATCACCAGAAGGAGCGTCTCCGTGGTTTTTTCAAACG |
| **58** | 46 | TTGAAATAGCAAACACAACCCGTCGGATGAATTATCAAAATAAACA |
| **59** | 42 | TTCCTGAGCGAGTAAAGAGAATCGATGACTAAATTATAAGAG |
| **60** | 42 | AATGTGATTATCAGCAGAGCCTAATTTGGCCAGTATAATGGT |
| **61** | 42 | AACGAGCAGTACCGTTTCATCTTCTGACACGGTAAAACATTA |
| **62** | 42 | AATATAAGTCTTTCATGATGGCAATTCATTTCATCTCGTAAA |
| **63** | 42 | TATATTTTGTCAATTTCCTGTAGCCAGCTCAATATCAACGCT |
| **64** | 42 | ATTGTTTTCTGGCCCATATGTACCCCGGAAAACTTTCCAGAC |
| **65** | 42 | ATTCGCGGGATTATCAATTTTATCCTGAAATTCTGTTTCAAA |
| **66** | 42 | TTTGCACATAAACAACAAAGAACGCGAGTTGATAAAAAAATA |
| **67** | 42 | GACGACACCAGCTAACTTCTGAATAATGCGCCATCTCAGAAA |
| **68** | 42 | AATCCAAAAAACAGTTAACCAATAGGAAGAAGGGTTTGCTAT |
| **69** | 41 | TACCATATCATTTTGAAGATTGTATAAGTAAATGCCGCGCC |
| **70** | 58 | TGTTTAAGCCAACATGTTTTTTTTAATTTAGGCGTGTGATAAATAAGGGTCATTGCCT |
| **71** | 59 | TAACAACCGTCAAACAAATGAAAAATCTGTTAGAATTTCGTCACCAGTAGAATCATAAT |
| **72** | 42 | CTGAGAGAAACAGGCCCATGTACCGTAAGCCTGTTCCATATT |
| **73** | 42 | ATAGCAGAGAATCGTAGTATCATATGCGATAGGAAAGGCCGA |
| **74** | 42 | AAGCCCATTATACAGTAGGGCTTAATTGCCTTTACTGCCACG |
| **75** | 42 | TTAAAGGGCAACAGAGAGAGAATAACATCTCAACAAATTCTT |
| **76** | 50 | AGAGAGAAGCCAACGAAAAACATATTAACACCGCCTGATTTTAGGATAGC |
| **77** | 55 | ACCAGTATTTTCAGGACAGGAACGGTACCGGTCAGGGGAAGCGCATTAGAATATC |

| | | |
|---|---|---|
| **78** | 42 | ACCGCCATTAAGCCGGTAATTGAGCGCTACGGGAGAAACAGA |
| **79** | 42 | CTCAGAAAGAAACAACCAAGTACACAAAGTCAGAGCAATAAT |
| **80** | 42 | CGAGAACGTATTAAATGAAATAGCAATATTAGTACAGAGTCT |
| **81** | 42 | AGAACGGAAGCAAGCATCGCCATTAAAAGAGTAAACGCCACC |
| **82** | 42 | TGATAGCACGCAAACGTACTCAGGAGGTGCTATCTCATTCCA |
| **83** | 42 | GTCCATCCCTAAAACCGTTTTTATTTTCTCCTTATTACCGAA |
| **84** | 46 | AATAGCAACAATCAATATTTTTCGGCTGTCTTATCGTAGCGCGAAC |
| **85** | 58 | TTGCTCCGAATTACCTTTTTTTTTATGCGATTTATCAAGAGTAATCTTTTGTATCATC |
| **86** | 59 | TCATTGTTTTTGATATGAGTGAGCTAACTTCCGAACAGCAGCGAAAGACCCCAAATCAA |
| **87** | 42 | GCCTGGGTTATAAACTTTTGCGGGATCGTGCTCATACTTTAA |
| **88** | 42 | CATTTTTAATTTCATCAGTGAATAAGGCAAGGCCGTCAAAAG |
| **89** | 42 | GGAGTTATTGCCCTGCTTGAGATGGTTTGCGGATGGTGTAAA |
| **90** | 42 | AATAGCCGCATAAAGCTTAGAGCTTAATAAATTGGGACGAGA |
| **91** | 50 | CAGTTGACGAGTAGTTGCTGAACATACGAGCCGGAACGAGATACTTGCAG |
| **92** | 55 | AACACCAGCTGAGGGGGTTGAGTGTTGTCACACAATATAATGCTGTAGCATATAA |
| **93** | 42 | CGCCCACAGTAGATGGAAGTTTCATTCCTCAACATTCCGCTC |
| **94** | 42 | GCCGACAAGATACAGGCTTTTGCAGTACGGTGTCTTTAGTTT |
| **95** | 42 | TTTGCCAAGCGAGATTTCGCAAATGGTCATACCGAAAAGGGC |
| **96** | 42 | CCAAAATGAGGGGGTTCGTAATCATGGTCAACGTCTAGTTGC |
| **97** | 42 | GTACCGACGTCTATTCTTAAACAGCTTGAATAACCATAAAAA |
| **98** | 42 | GAAAAACGCTCGAATAATAGTAAAATGTGACGACGTGTTTAG |
| **99** | 46 | GCGGAATCTATCATAACTTTTTTCGTTTACCATTAGACTTCCCCGG |
| **100** | 50 | AGAAGGATTTTTTTAACCGAGGAAACGCAATAATAAGTACCAGCTCAAAC |
| **101** | 42 | GAACTGGGGATTAGGTAATATCCAGAACCCAACAGAATTTAC |
| **102** | 39 | ACGACCAAACAGGATATTAAGAGGCTGACGGTATAAACA |
| **103** | 48 | GTTAATGCCCATGAAAGAAAACGCTCATGGAGATTCACACAGGAGTGT |
| **104** | 46 | GCGATTAGGTAAAAAGACGGGCAACAGCACGCGCGTCAGACTGTAG |
| **105** | 42 | GAATCGGCCATGATTGCGTTTCCATTAAACGTACCAAGCGCG |
| **106** | 45 | GCCTGATAAAGACCTTCTAAGAACTGGCTCACAAAGCGGCGTTTG |
| **107** | 42 | AGCCGGAAGGACAGAATCTACGTTAATAGAAGCCCCCACCAC |
| **108** | 42 | ATGTGAGATGGAAAAGGTAGAAAGATTCTATAGTCCCGCCAC |
| **109** | 42 | TTAATTTTTTTTAACAATTATCGTCGCTTTTTATTAATTTTC |
| **110** | 44 | CCTTAGAATCTCCGGCATAAGTCCTGAACAGAGGCGTGCCGCCG |
| **111** | 42 | TGGGTTTTTTTACTATATGCAAATATTTTTTTTGTAAACGTT |
| **112** | 39 | TAAACAGCATAACGGATGAAACAAACATTTCCTGATTGC |
| **113** | 48 | TTTGAATACCAGAAGATCCAAAAGGAATTACCCCCTCATCAGATGAAT |
| **114** | 50 | TCTACTATTTTTTTATAGTAGTAGCATTATATATTTAAAAAAACGTAAAG |
| **115** | 42 | GAGTAATTTTTCACCCTAAAGGGAGCCCGGTAACGACGCCAG |
| **116** | 42 | ATATGATTTTCAACGGGAAGAAAGCGAACTAATAGTTAAATC |
| **117** | 46 | AGGGTAGCGTTAGTGTAGCGGTCACGCTATTGAGGTAACATTATCA |
| **118** | 42 | GTTGAAAGGAGCGCGTAGTCGTCTTTCCAGACTATTTTTGAG |

| | | |
|---|---|---|
| **119** | 45 | GAGAGTCTGGACCGACCAGAGGCATTTTCGACCAGTTACATCATA |
| **120** | 42 | ACTAGCATAGTTAAACAAAAGGTAAAGTATCTTACAATCCTG |
| **121** | 42 | AGCCCCATCGCAAGACATGTTCAGCTAAAGATTAGTAGAACC |
| **122** | 46 | TACTAGAAAAACACTGAGTCAGAGCGGGAGCTCCAGCAGAATGAAA |
| **123** | 39 | GGTGAGGGCCAGAAAGCCACCACCCTCATAATAACCCAC |
| **124** | 48 | AAGAATTGAGCCCTCAGTCCTGAGAAGTGTTGAAGATAAATTAACTGA |
| **125** | 42 | AAGAGCACCGCCACTCAGTGAGGCCACCATACCGACACTCAT |
| **126** | 57 | GCCCTTTGTATCACTTAACCGTTGTAGCTTTAATGGAATCATTATTTTTTGCGCCC |
| **127** | 58 | AATAGGTTTAAGAAAATTTTTTAAGCAGATAGCCGAACATAGTGAATTTAGAGCATG |
| **128** | 46 | CGTAACAAAGCTCACCCTATCGGCAAAATCCCGTGCCTAAAGAGGT |
| **129** | 39 | ACAATTCTCCAGTTCGATATATTCGGTCGAATTCCCAAT |
| **130** | 48 | TCTGCGAACGGCATAACTGGAACAAGAGTCCATTGTTAGTTTTAAATA |
| **131** | 42 | GACCATTATGACAAAAGAACGTGGACTCCATAGCTAAGAAGT |
| **132** | 57 | CTATATTGTGAATTCAGGGCGATGGCCCTAGAGGAGGATAGCGTTTTTTTAATACT |
| **133** | 58 | TTTCGAGTTCATTTGGTTTTTTTCGCGAGCTGAAAAGGTTTATTCATTTCAAGTAAGA |
| **134** | 39 | ACTGGTAATCATATTTACATTGGCAAATACCTTATTATT |
| **135** | 42 | AAAACGAAAGCCACATTTTGACGCTCAATCGGGCACCAACCT |
| **136** | 42 | AAGCGGATTATTACCAGTACATAGGAACCGAACTGCTAACGG |
| **137** | 27 | TGTTTATTTTTTCAACAATAGTTAGGT |
| **138** | 27 | TAGGAATTTTTTACCACATTCACAAAA |
| **139** | 39 | ATACAGTAAGCATATTCATTGAATCGAGGCATATTACCT |
| **140** | 42 | GACGGGGTACTGCAAGGCGATTAAGTTGCCGATTTTGCGAAT |
| **141** | 39 | ACACCCTGACGACGAACCACCAGCATTTATAACCTCAGA |
| **142** | 39 | TGCAACTAAAAGTTTCCTGTGTGAAACTATTACAACCAT |
| **143** | 57 | TTGCCTGAGTCGTGGCACAGACAATATTTTATGGCTATTAGTCAATACTTAGCCCGG |
| **144** | 50 | TAGAAACGCAAATCTTGGCCTTGATATTCACAAACTTTTTTTAAATAAAT |
| **145** | 50 | GCAACACGTCATAAGCCATTCGCCATTCAGGCTGCTTTTTTTGCAACTGT |
| **146** | 57 | TTTTGGGGTCGGCCAGTGCCAAGCTTGCATTGCAGGTCGACTCACTACGTAGCTTGC |
| **147** | 21 | TTTCCTCAAAGCATGATTTTT |
| **148** | 21 | ATGGTGGTCACATTCCTTTAA |
| **149** | 44 | GCCTTTAATTGTATTTTATCGAACCATCTTTTTCAAATCAAGTT |
| **150** | 41 | AAACACGGAGATGACAAGTTTTCATTAAGCATCGCTGTTTG |
| **151** | 39 | TTTTTCATGAGGAACCTTCACCGCCTGGGCTGCATTAAT |
| **152** | 54 | TTGCGCTGAAAATCGAACGAGGGTAGCAACGGCTACAGATTTAGGACTAAAGAC |
| **153** | 49 | CGCTGGTTTGCCCCAGCAGGCCACTGCCCGCTTTCCAGTTTTCCTGTCG |
| **154** | 26 | TGCCACCCTGAGAGAGTTGCAGCCCA |
| **155** | 34 | CGCGGGCATTTCAACAGGTTGAGAGTAAATTGCG |
| **156** | 44 | GTCGAGAGGGTTGTAAGTATCTTTGATTTTTTTAATAACATCAC |
| **157** | 41 | AGATGCTATCAGCGTTAATTAACACCGCAAACTAAACGTGC |
| **158** | 39 | CGATCTAAAGTTTTACCACCACACCCGCGCAAATCAACA |
| **159** | 54 | CTGAACCCACGTATCAACGCCTGTAGCATTCCACAGACATTTTAGTTAGCGTAA |

| | | |
|---|---|---|
| **160** | 49 | TACTATGGTTGCTTTGACGAGTCAAATATCAAACCCTCATTTTCTGGTC |
| **161** | 26 | AGTTGCGCGCTTAATGCGCCGCTGCG |
| **162** | 34 | TTTTAAGAAACTTATCCCTTAAGAAACCACCTTG |
| **5 nt anti-crossover strands** | | |
| **6_5nt** | 47 | CGTTCCAAGCAAACAAGACTCCTTATTAGACTCCTCCGCCAGGGGCG |
| **55_5nt** | 37 | CTTATCTTTTATTAATTTTAAAAATTGACCGCCGGAG |
| **13_5nt** | 37 | TCGCGTATTGAATCAAGTTTGCCGCAAGGCACCCCCA |
| **46_5nt** | 47 | CTGGCGAATCGGCCTAAAGATTCAAAAGAAGGAATAGAGCTTTCTTT |
| **80_5nt** | 47 | CGAGAACGTATTAAATGAAATAGCAATATTAGTACAGAGTCTATGGT |
| **148_5nt** | 16 | GGTCACATTCCTTTAA |
| **95_5nt** | 47 | TTTGCCAAGCGAGATTTCGCAAATGGTCATACCGAAAAGGGCTTTCC |
| **147_5nt** | 16 | TCAAAGCATGATTTTT |
| **8 nt anti-crossover strands** | | |
| **6_8nt** | 50 | CGTTCCAAGCAAACAAGACTCCTTATTAGACTCCTCCGCCAGGGGCGCTT |
| **55_8nt** | 34 | ATCTTTTATTAATTTTAAAAATTGACCGCCGGAG |
| **13_8nt** | 34 | CGTATTGAATCAAGTTTGCCGCAAGGCACCCCCA |
| **46_8nt** | 50 | CTGGCGAATCGGCCTAAAGATTCAAAAGAAGGAATAGAGCTTTCTTTTCG |
| **80_8nt** | 50 | CGAGAACGTATTAAATGAAATAGCAATATTAGTACAGAGTCTATGGTGGT |
| **148_8nt** | 13 | CACATTCCTTTAA |
| **95_8nt** | 50 | TTTGCCAAGCGAGATTTCGCAAATGGTCATACCGAAAAGGGCTTTCCTCA |
| **147_8nt** | 13 | AAGCATGATTTTT |
| **QD/QR binding staples** | | |
| **119_b** | 61 | GAGAGTCTGGACCGACCAGAGGCATTTTCGACCAGTTACATCATATTTAGAGAACCTGGG |
| **63_b** | 58 | TATATTTTGTCAATTTCCTGTAGCCAGCTCAATATCAACGCTTTTAGAGAACCTGGG |
| **120_b** | 58 | ACTAGCATAGTTAAACAAAAGGTAAAGTATCTTACAATCCTGTTTAGAGAACCTGGG |
| **68_b** | 58 | AATCCAAAAAACAGTTAACCAATAGGAAGAAGGGTTTGCTATTTTAGAGAACCTGGG |
| **121_b** | 58 | AGCCCCATCGCAAGACATGTTCAGCTAAAGATTAGTAGAACCTTTAGAGAACCTGGG |
| **face-selecting overhang staples** | | |
| **5_fs** | 62 | AGGATTACATGATTGTAGAAAATACATAGTCTCTGAGATAGATTTTTTTTTTTTTTTTTTTT |
| **9_fs** | 62 | CTGAAACCCTGCCTGGTCAGTGCCTTGATGATGATCAGTCACTTTTTTTTTTTTTTTTTTTT |
| **21_fs** | 62 | TTGAAAGACGAGGCGGTGAATTATCACCACCAGAGGAAAGACTTTTTTTTTTTTTTTTTTT T |
| **25_fs** | 62 | TTTTTTATGAATAATGAGGGAGGGAAGGCTCAGAAAGAAGCATTTTTTTTTTTTTTTTTTTT |
| **29_fs** | 64 | TTTTAACCCTTGAATGGTTTACCAGCGCCACCAGATTTAGCGAATTTTTTTTTTTTTTTTTTT TT |
| **34_fs** | 59 | GAAGAGTCAAAACGGAATAAGTTTACAGACGAAGATATATTTTTTTTTTTTTTTTTTTT |
| **37_fs** | 51 | GATTCGAAAATTCGCGAAATTGAACGAGAATTTTTTTTTTTTTTTTTTTTT |
| **39_fs** | 62 | GAGCAAAAAGTTACCATCGGGAGAAACATTTAACGAATGCTTTTTTTTTTTTTTTTTTTTTT |
| **45_fs** | 62 | AATAATTGTGTAGGTCAGGAAGATCGCACGCTATTCCAGGGTTTTTTTTTTTTTTTTTTTTT |
| **47_fs** | 75 | GAGGATTAAAGCCGGAAAGGAACAACTAGGTGAGAGGGACGACGACAGTAAGGGGTTTT TTTTTTTTTTTTTTTT |
| **49_fs** | 62 | GCGGAGTTCAAATCCCGTGCATCTGCCAGACTTTATACATTTTTTTTTTTTTTTTTTTTTTT |
| **54_fs** | 62 | TTTTCTGAATTAATGTAATGGGATAGGTCGAACGTAGGAGCATTTTTTTTTTTTTTTTTTTT |

| | | |
|---|---|---|
| **74_fs** | 62 | AAGCCCATTATACAGTAGGGCTTAATTGCCTTTACTGCCACGTTTTTTTTTTTTTTTTTTTT |
| **78_fs** | 62 | ACCGCCATTAAGCCGGTAATTGAGCGCTACGGGAGAAACAGATTTTTTTTTTTTTTTTTTT<br>T |
| **89_fs** | 62 | GGAGTTATTGCCCTGCTTGAGATGGTTTGCGGATGGTGTAAATTTTTTTTTTTTTTTTTTTT |
| **93_fs** | 62 | CGCCCACAGTAGATGGAAGTTTCATTCCTCAACATTCCGCTCTTTTTTTTTTTTTTTTTTTT |
| **101_fs** | 62 | GAACTGGGGATTAGGTAATATCCAGAACCCAACAGAATTTACTTTTTTTTTTTTTTTTTTTT |
| **103_fs** | 68 | GTTAATGCCCATGAAAGAAAACGCTCATGGAGATTCACACAGGAGTGTTTTTTTTTTTTTT<br>TTTTTTT |
| **106_fs** | 65 | GCCTGATAAAGACCTTCTAAGAACTGGCTCACAAAGCGGCGTTTGTTTTTTTTTTTTTTTTT<br>TTT |
| **107_fs** | 62 | AGCCGGAAGGACAGAATCTACGTTAATAGAAGCCCCCACCACTTTTTTTTTTTTTTTTTTT<br>T |
| **108_fs** | 62 | ATGTGAGATGGAAAAGGTAGAAAGATTCTATAGTCCCGCCACTTTTTTTTTTTTTTTTTTTT |
| **110_fs** | 64 | CCTTAGAATCTCCGGCATAAGTCCTGAACAGAGGCGTGCCGCCGTTTTTTTTTTTTTTTTTT<br>TT |
| **113_fs** | 68 | TTTGAATACCAGAAGATCCAAAAGGAATTACCCCCTCATCAGATGAATTTTTTTTTTTTTT<br>TTTTTTT |
| **115_fs** | 62 | GAGTAATTTTTCACCCTAAAGGGAGCCCGGTAACGACGCCAGTTTTTTTTTTTTTTTTTTTT |
| **116_fs** | 62 | ATATGATTTTCAACGGGAAGAAAGCGAACTAATAGTTAAATCTTTTTTTTTTTTTTTTTTTT |
| **122_fs** | 66 | TACTAGAAAAACACTGAGTCAGAGCGGGAGCTCCAGCAGAATGAAATTTTTTTTTTTTTTT<br>TTTTT |
| **124_fs** | 68 | AAGAATTGAGCCCTCAGTCCTGAGAAGTGTTGAAGATAAATTAACTGATTTTTTTTTTTTT<br>TTTTTTT |
| **125_fs** | 62 | AAGAGCACCGCCACTCAGTGAGGCCACCATACCGACACTCATTTTTTTTTTTTTTTTTTTTT |
| **128_fs** | 66 | CGTAACAAAGCTCACCCTATCGGCAAAATCCCGTGCCTAAAGAGGTTTTTTTTTTTTTTTT<br>TTTTT |
| **130_fs** | 68 | TCTGCGAACGGCATAACTGGAACAAGAGTCCATTGTTAGTTTAAATATTTTTTTTTTTTTTT<br>TTTTTT |
| **131_fs** | 62 | GACCATTATGACAAAAGAACGTGGACTCCATAGCTAAGAAGTTTTTTTTTTTTTTTTTTTTT |

## REFERENCES


1. F. P. García de Arquer, D. V. Talapin, V. I. Klimov, Y. Arakawa, M. Bayer, E. H. Sargent, Semiconductor quantum dots: Technological progress and future challenges. *Science* **373**, eaaz8541 (2021).

2. Y. Shirasaki, G. J. Supran, M. G. Bawendi, V. Bulović, Emergence of colloidal quantum-dot light-emitting technologies. *Nat. Photonics* **7**, 13–23 (2013).

3. X. Li, Y.-B. Zhao, F. Fan, L. Levina, M. Liu, R. Quintero-Bermudez, X. Gong, L. N. Quan, J. Fan, Z. Yang, S. Hoogland, O. Voznyy, Z.-H. Lu, E. H. Sargent, Bright colloidal quantum dot light-emitting diodes enabled by efficient chlorination. *Nat. Photonics* **12**, 159–164 (2018).

4. A. K. Srivastava, W. Zhang, J. Schneider, J. E. Halpert, A. L. Rogach, Luminescent down-conversion semiconductor quantum dots and aligned quantum rods for liquid crystal displays. *Adv. Sci.* **6**, 1901345 (2019).

5. K. Behrman, I. Kymissis, Micro light-emitting diodes. *Nat. Electron.* **5**, 564–573 (2022).

6. J. Hu, L. Li, W. Yang, L. Manna, L. Wang, A. P. Alivisatos, Linearly polarized emission from colloidal semiconductor quantum rods. *Science* **292**, 2060–2063 (2001).

7. M. F. Prodanov, C. Kang, S. K. Gupta, V. V. Vashchenko, Y. Li, M. Qin, X. Lu, A. K. Srivastava, Unidirectionally aligned bright quantum rods films, using T-shape ligands, for LCD application. *Nano Res.* **15**, 5392–5401 (2022).

8. K.-H. Kim, J.-J. Kim, Origin and control of orientation of phosphorescent and TADF dyes for high-efficiency OLEDs. *Adv. Mater.* **30**, 1705600 (2018).

9. S. Nam, N. Oh, Y. Zhai, M. Shim, High efficiency and optical anisotropy in double-heterojunction nanorod light-emitting diodes. *ACS Nano* **9**, 878–885 (2015).

10. S. Rhee, J. H. Chang, D. Hahm, B. G. Jeong, J. Kim, H. Lee, J. Lim, E. Hwang, J. Kwak, W. K. Bae, Tailoring the electronic landscape of quantum dot light-emitting diodes for high brightness and stable operation. *ACS Nano* **14**, 17496–17504 (2020).

11. A. K. Srivastava, W. Zhang, J. Schneider, A. L. Rogach, V. G. Chigrinov, H.-S. Kwok, Photoaligned nanorod enhancement films with polarized emission for liquid-crystal-display applications. *Adv. Mater.* **29**, 1701091 (2017).

12. J. Kim, J. Peretti, K. Lahlil, J.-P. Boilot, T. Gacoin, Optically anisotropic thin films by shear-oriented assembly of colloidal nanorods. *Adv. Mater.* **25**, 3295–3300 (2013).

13. Z. Hu, M. D. Fischbein, C. Querner, M. Drndić, Electric-field-driven accumulation and alignment of CdSe and CdTe nanorods in nanoscale devices. *Nano Lett.* **6**, 2585–2591 (2006).

14. F. Pietra, F. T. Rabouw, P. G. van Rhee, J. van Rijssel, A. V. Petukhov, B. H. Erné, P. C. M. Christianen, C. de Mello Donegá, D. Vanmaekelbergh, Self-assembled CdSe/CdS nanorod sheets studied in the bulk suspension by magnetic alignment. *ACS Nano* **8**, 10486–10495 (2014).

15. A. Rizzo, C. Nobile, M. Mazzeo, M. De Giorgi, A. Fiore, L. Carbone, R. Cingolani, L. Manna, G. Gigli, Polarized light emitting diode by long-range nanorod self-assembling on a water surface. *ACS Nano* **3**, 1506–1512 (2009).

16. S. Rhee, D. Jung, D. Kim, D. C. Lee, C. Lee, J. Roh, Polarized electroluminescence emission in high-performance quantum rod light-emitting diodes via the langmuir-blodgett technique. *Small* **17**, 2101204 (2021).

17. P. D. Cunningham, J. B. Souza, I. Fedin, C. She, B. Lee, D. V. Talapin, Assessment of anisotropic semiconductor nanorod and nanoplatelet heterostructures with polarized emission for liquid crystal display technology. *ACS Nano* **10**, 5769–5781 (2016).

18. T. Du, J. Schneider, A. K. Srivastava, A. S. Susha, V. G. Chigrinov, H. S. Kwok, A. L. Rogach, Combination of photoinduced alignment and self-assembly to realize polarized emission from ordered semiconductor nanorods. *ACS Nano* **9**, 11049–11055 (2015).

19. J. Schneider, W. Zhang, A. K. Srivastava, V. G. Chigrinov, H.-S. Kwok, A. L. Rogach, Photoinduced micropattern alignment of semiconductor nanorods with polarized emission in a liquid crystal polymer matrix. *Nano Lett.* **17**, 3133–3138 (2017).

20. L. Sun, J. J. Choi, D. Stachnik, A. C. Bartnik, B.-R. Hyun, G. G. Malliaras, T. Hanrath, F. W. Wise, Bright infrared quantum-dot light-emitting diodes through inter-dot spacing control. *Nat. Nanotechnol.* **7**, 369–373 (2012).

21. C. Kang, M. F. Prodanov, Y. Gao, K. Mallem, Z. Yuan, V. V. Vashchenko, A. K. Srivastava, Quantum-rod on-chip LEDs for display backlights with efficacy of 149 lm $W^{-1}$: A step toward 200 lm $W^{-1}$. *Adv. Mater.* **33**, 2104685 (2021).

22. Z. Liu, C.-H. Lin, B.-R. Hyun, C.-W. Sher, Z. Lv, B. Luo, F. Jiang, T. Wu, C.-H. Ho, H.-C. Kuo, J.-H. He, Micro-light-emitting diodes with quantum dots in display technology. *Light Sci. Appl.* **9**, 83 (2020).

23. S. Hepp, M. Jetter, S. L. Portalupi, P. Michler, Semiconductor quantum dots for integrated quantum photonics. *Adv. Quantum Tech.* **2**, 1900020 (2019).

24. A. W. Elshaari, W. Pernice, K. Srinivasan, O. Benson, V. Zwiller, Hybrid integrated quantum photonic circuits. *Nat. Photonics* **14**, 285–298 (2020).

25. G. Rainò, M. A. Becker, M. I. Bodnarchuk, R. F. Mahrt, M. V. Kovalenko, T. Stöferle, Superfluorescence from lead halide perovskite quantum dot superlattices. *Nature* **563**, 671–675 (2018).

26. T. Tørring, N. V. Voigt, J. Nangreave, H. Yan, K. V. Gothelf, DNA origami: A quantum leap for self-assembly of complex structures. *Chem. Soc. Rev.* **40**, 5636–5646 (2011).

27. N. Liu, T. Liedl, DNA-assembled advanced plasmonic architectures. *Chem. Rev.* **118**, 3032–3053 (2018).

28. A. Gopinath, E. Miyazono, A. Faraon, P. W. K. Rothemund, Engineering and mapping nanocavity emission via precision placement of DNA origami. *Nature* **535**, 401–405 (2016).

29. A. Gopinath, C. Thachuk, A. Mitskovets, H. A. Atwater, D. Kirkpatrick, P. W. K. Rothemund, Absolute and arbitrary orientation of single-molecule shapes. *Science* **371**, eabd6179 (2021).

30. C. Chen, X. Wei, M. F. Parsons, J. Guo, J. L. Banal, Y. Zhao, M. N. Scott, G. S. Schlau-Cohen, R. Hernandez, M. Bathe, Nanoscale 3D spatial addressing and valence control of quantum dots using wireframe DNA origami. *Nat. Commun.* **13**, 4935 (2022).

31. A. Banerjee, T. Pons, N. Lequeux, B. Dubertret, Quantum dots–DNA bioconjugates: Synthesis to applications. *Interface Focus* **6**, 20160064 (2016).

32. G. P. Mitchell, C. A. Mirkin, R. L. Letsinger, Programmed assembly of DNA functionalized quantum dots. *J. Am. Chem. Soc.* **121**, 8122–8123 (1999).

33. R. Gill, I. Willner, I. Shweky, U. Banin, Fluorescence resonance energy transfer in CdSe/ZnS−DNA Conjugates: Probing hybridization and DNA cleavage. *J. Phys. Chem. B* **109**, 23715–23719 (2005).

34. D. Zhou, J. D. Piper, C. Abell, D. Klenerman, D.-J. Kang, L. Ying, Fluorescence resonance energy transfer between a quantum dot donor and a dye acceptor attached to DNA. *Chem. Commun.*, 4807–4809 (2005).

35. I. L. Medintz, H. T. Uyeda, E. R. Goldman, H. Mattoussi, Quantum dot bioconjugates for imaging, labelling and sensing. *Nat. Mater.* **4**, 435–446 (2005).

36. P. Rahmani, M. Goodlad, Y. Zhang, Y. Li, T. Ye, One-step ligand-exchange method to produce quantum dot–DNA conjugates for DNA-directed self-assembly. *ACS Appl. Mater. Interfaces* **14**, 47359–47368 (2022).

37. L. M. Demers, C. A. Mirkin, R. C. Mucic, R. A. Reynolds, R. L. Letsinger, R. Elghanian, G. Viswanadham, A fluorescence-based method for determining the surface coverage and hybridization efficiency of thiol-capped oligonucleotides bound to gold thin films and nanoparticles. *Anal. Chem.* **72**, 5535–5541 (2000).

38. Z. Deng, A. Samanta, J. Nangreave, H. Yan, Y. Liu, Robust DNA-functionalized core/shell quantum dots with fluorescent emission spanning from UV–vis to Near-IR and compatible with DNA-directed self-assembly. *J. Am. Chem. Soc.* **134**, 17424–17427 (2012).

39. T. Zhang, T. Liedl, DNA-based assembly of quantum dots into dimers and helices. *Nanomaterials* **9**, 339 (2019).

40. G. Tikhomirov, S. Hoogland, P. E. Lee, A. Fischer, E. H. Sargent, S. O. Kelley, DNA-based programming of quantum dot valency, self-assembly and luminescence. *Nat. Nanotechnol.* **6**, 485–490 (2011).

41. Z. Cao, Y. Shu, H. Qin, B. Su, X. Peng, Quantum dots with highly efficient, stable, and multicolor electrochemiluminescence. *ACS Cent. Sci.* **6**, 1129–1137 (2020).

42. H. Jun, X. Wang, W. P. Bricker, M. Bathe, Automated sequence design of 2D wireframe DNA origami with honeycomb edges. *Nat. Commun.* **10**, 5419 (2019).

43. X. Wang, S. Li, H. Jun, T. John, K. Zhang, H. Fowler, J. P. K. Doye, W. Chiu, M. Bathe, Planar 2D wireframe DNA origami. *Sci. Adv.* **8**, eabn0039 (2022).

44. X. Wang, H. Jun, M. Bathe, Programming 2D supramolecular assemblies with wireframe DNA origami. *J. Am. Chem. Soc.* **144**, 4403–4409 (2022).

45. N. Avakyan, J. W. Conway, H. F. Sleiman, Long-range ordering of blunt-ended DNA tiles on supported lipid bilayers. *J. Am. Chem. Soc.* **139**, 12027–12034 (2017).

46. Y. Suzuki, M. Endo, H. Sugiyama, Lipid-bilayer-assisted two-dimensional self-assembly of DNA origami nanostructures. *Nat. Commun.* **6**, 8052 (2015).

47. Y. Suzuki, H. Sugiyama, M. Endo, Complexing DNA origami frameworks through sequential self-assembly based on directed docking. *Angew. Chem. Int. Ed.* **57**, 7061–7065 (2018).

48. S. Kocabey, S. Kempter, J. List, Y. Xing, W. Bae, D. Schiffels, W. M. Shih, F. C. Simmel, T. Liedl, Membrane-assisted growth of dna origami nanostructure arrays. *ACS Nano* **9**, 3530–3539 (2015).

49. S. Kempter, A. Khmelinskaia, M. T. Strauss, P. Schwille, R. Jungmann, T. Liedl, W. Bae, Single particle tracking and super-resolution imaging of membrane-assisted stop-and-go diffusion and lattice assembly of DNA origami. *ACS Nano* **13**, 996–1002 (2019).

50. S. Woo, P. W. K. Rothemund, Self-assembly of two-dimensional DNA origami lattices using cation-controlled surface diffusion. *Nat. Commun.* **5**, 4889 (2014).

51. A. Aghebat Rafat, T. Pirzer, M. B. Scheible, A. Kostina, F. C. Simmel, Surface-assisted large-scale ordering of DNA origami tiles. *Angew. Chem. Int. Ed.* **53**, 7665–7668 (2014).

52. Y. Xin, S. Martinez Rivadeneira, G. Grundmeier, M. Castro, A. Keller, Self-assembly of highly ordered DNA origami lattices at solid-liquid interfaces by controlling cation binding and exchange. *Nano Res.* **13**, 3142–3150 (2020).

53. C. Kielar, S. Ramakrishnan, S. Fricke, G. Grundmeier, A. Keller, Dynamics of DNA origami lattice formation at solid–liquid interfaces. *ACS Appl. Mater. Interfaces* **10**, 44844–44853 (2018).

54. Y. Xin, B. Shen, M. A. Kostiainen, G. Grundmeier, M. Castro, V. Linko, A. Keller, Scaling up DNA origami lattice assembly. *Chem. A Eur. J.* **27**, 8564–8571 (2021).

55. K. Tapio, C. Kielar, J. M. Parikka, A. Keller, H. Järvinen, K. Fahmy, J. J. Toppari, Large-scale formation of DNA origami lattices on silicon. *Chem. Mater.* **35**, 1961–1971 (2023).

56. Y. Hao, Y. Li, L. Song, Z. Deng, Flash synthesis of spherical nucleic acids with record DNA density. *J. Am. Chem. Soc.* **143**, 3065–3069 (2021).

57. S. F. Wuister, I. Swart, F. van Driel, S. G. Hickey, C. de Mello Donegá, Highly luminescent water-soluble CdTe quantum dots. *Nano Lett.* **3**, 503–507 (2003).

58. J. Farlow, D. Seo, K. E. Broaders, M. J. Taylor, Z. J. Gartner, Y. Jun, Formation of targeted monovalent quantum dots by steric exclusion. *Nat. Methods* **10**, 1203–1205 (2013).

59. S. Tamang, G. Beaune, I. Texier, P. Reiss, Aqueous phase transfer of InP/ZnS nanocrystals conserving fluorescence and high colloidal stability. *ACS Nano* **5**, 9392–9402 (2011).

60. C. Zhang, R. J. Macfarlane, K. L. Young, C. H. J. Choi, L. Hao, E. Auyeung, G. Liu, X. Zhou, C. A. Mirkin, A general approach to DNA-programmable atom equivalents. *Nat. Mater.* **12**, 741–746 (2013).

61. C. Chen, B. Corry, L. Huang, N. Hildebrandt, FRET-modulated multihybrid nanoparticles for brightness-equalized single-wavelength barcoding. *J. Am. Chem. Soc.* **141**, 11123–11141 (2019).

62. A. R. Clapp, I. L. Medintz, J. M. Mauro, B. R. Fisher, M. G. Bawendi, H. Mattoussi, fluorescence resonance energy transfer between quantum dot donors and dye-labeled protein acceptors. *J. Am. Chem. Soc.* **126**, 301–310 (2004).

63. O. Kratky, G. Porod, Röntgenuntersuchung gelöster Fadenmoleküle. *Recl. Trav. Chim. Pays-Bas.* **68**, 1106–1122 (1949).

64. S. B. Smith, Y. Cui, C. Bustamante, Overstretching B-DNA: The elastic response of individual double-stranded and single-stranded DNA molecules. *Science* **271**, 795–799 (1996).

65. H. Jun, X. Wang, M. F. Parsons, W. P. Bricker, T. John, S. Li, S. Jackson, W. Chiu, M. Bathe, Rapid prototyping of arbitrary 2D and 3D wireframe DNA origami. *Nucleic Acids Res.* **49**, 10265–10274 (2021).

66. G. Tikhomirov, P. Petersen, L. Qian, Triangular DNA origami tilings. *J. Am. Chem. Soc.* **140**, 17361–17364 (2018).

67. G. Tikhomirov, P. Petersen, L. Qian, Fractal assembly of micrometre-scale DNA origami arrays with arbitrary patterns. *Nature* **552**, 67–71 (2017).

68. Y. Liu, Y. Ke, H. Yan, Self-assembly of symmetric finite-size DNA nanoarrays. *J. Am. Chem. Soc.* **127**, 17140–17141 (2005).

69. C. Vietz, B. Lalkens, G. P. Acuna, P. Tinnefeld, Functionalizing large nanoparticles for small gaps in dimer nanoantennas. *New J. Phys.* **18**, 045012 (2016).

70. K. Trofymchuk, V. Glembockyte, L. Grabenhorst, F. Steiner, C. Vietz, C. Close, M. Pfeiffer, L. Richter, M. L. Schütte, F. Selbach, R. Yaadav, J. Zähringer, Q. Wei, A. Ozcan, B. Lalkens, G. P. Acuna, P. Tinnefeld, Addressable nanoantennas with cleared hotspots for single-molecule detection on a portable smartphone microscope. *Nat. Commun.* **12**, 950 (2021).

71. C. Hartl, K. Frank, H. Amenitsch, S. Fischer, T. Liedl, B. Nickel, Position accuracy of gold nanoparticles on DNA origami structures studied with small-angle x-ray scattering. *Nano Lett.* **18**, 2609–2615 (2018).

72. A. Sitt, A. Salant, G. Menagen, U. Banin, Highly emissive nano rod-in-rod heterostructures with strong linear polarization. *Nano Lett.* **11**, 2054–2060 (2011).

73. I. Hadar, G. B. Hitin, A. Sitt, A. Faust, U. Banin, Polarization properties of semiconductor nanorod heterostructures: From single particles to the ensemble. *J. Phys. Chem. Lett.* **4**, 502–507 (2013).

74. W. W. Yu, L. Qu, W. Guo, X. Peng, Experimental determination of the extinction coefficient of CdTe, CdSe, and CdS nanocrystals. *Chem. Mater.* **15**, 2854–2860 (2003).

75. E. Shaviv, A. Salant, U. Banin, Size dependence of molar absorption coefficients of CdSe semiconductor quantum rods. *ChemPhysChem* **10**, 1028–1031 (2009).

76. P. Adel, J. Bloh, D. Hinrichs, T. Kodanek, D. Dorfs, Determination of all dimensions of CdSe Seeded CdS nanorods solely via their UV/Vis spectra. *Z. Phys. Chem.* **231**, 93–106 (2017).

77. R. Rezakhaniha, A. Agianniotis, J. T. C. Schrauwen, A. Griffa, D. Sage, C. V. C. Bouten, F. N. Van De Vosse, M. Unser, N. Stergiopulos, Experimental investigation of collagen waviness and orientation in the arterial adventitia using confocal laser scanning microscopy. *Biomech. Model. Mechanobiol.* **11**, 461–473 (2012).

78. E. Fonck, G. G. Feigl, J. Fasel, D. Sage, M. Unser, D. A. Rüfenacht, N. Stergiopulos, Effect of aging on elastin functionality in human cerebral arteries. *Stroke* **40**, 2552–2556 (2009).

79. C. Würth, M. Grabolle, J. Pauli, M. Spieles, U. Resch-Genger, Relative and absolute determination of fluorescence quantum yields of transparent samples. *Nat. Protoc.* **8**, 1535–1550 (2013).

80. W. R. Algar, N. Hildebrandt, S. S. Vogel, I. L. Medintz, FRET as a biomolecular research tool — Understanding its potential while avoiding pitfalls. *Nat. Methods* **16**, 815–829 (2019).

81. H. Li, B. Zhang, X. Lu, X. Tan, F. Jia, Y. Xiao, Z. Cheng, Y. Li, D. O. Silva, H. S. Schrekker, K. Zhang, C. A. Mirkin, Molecular spherical nucleic acids. *Proc. Natl. Acad. Sci. U.S.A.* **115**, 4340–4344 (2018).

82. X. Wei, C. Chen, Y. Zhao, E. Harazinska, M. Bathe, R. Hernandez, Molecular structure of single-stranded DNA on the ZnS surface of quantum dots. *ACS Nano* **16**, 6666–6675 (2022).
